# Synthesis of Biotin-Tagged Chitosan Oligosaccharides and Assessment of Their Immunomodulatory Activity

**DOI:** 10.3389/fchem.2020.554732

**Published:** 2020-12-01

**Authors:** Yury E. Tsvetkov, Ema Paulovičová, Lucia Paulovičová, Pavol Farkaš, Nikolay E. Nifantiev

**Affiliations:** ^1^Laboratory of Glycoconjugate Chemistry, N.D. Zelinsky Institute of Organic Chemistry, Russian Academy of Sciences, Moscow, Russia; ^2^Cell Culture & Immunology Laboratory, Department of Immunochemistry of Glycoconjugates, Center for Glycomics, Institute of Chemistry, Slovak Academy of Sciences, Bratislava, Slovakia

**Keywords:** chitin, chitosan, chitosan oligosaccharides, synthesis, glycosylation, aglycon transfer, cytokines, RAW 264.7

## Abstract

Chitin, a polymer of β-(1→4)-linked *N*-acetyl-d-glucosamine, is one of the main polysaccharide components of the fungal cell wall. Its N-deacetylated form, chitosan, is enzymatically produced in the cell wall by chitin deacetylases. It exerts immunomodulative, anti-inflammatory, anti-cancer, anti-bacterial, and anti-fungal activities with various medical applications. To study the immunobiological properties of chitosan oligosaccharides, we synthesized a series of β-(1→4)-linked *N*-acetyl-d-glucosamine oligomers comprising 3, 5, and 7 monosaccharide units equipped with biotin tags. The key synthetic intermediate employed for oligosaccharide chain elongation, a disaccharide thioglycoside, was prepared by orthogonal glycosylation of a 4-OH thioglycoside acceptor with a glycosyl trichloroacetimidate bearing the temporary 4-*O*-*tert*-butyldimethylsilyl group. The use of silyl protection suppressed aglycon transfer and provided a high yield for the target disaccharide donor. Using synthesized chitosan oligomers, as well as previously obtained chitin counterparts, the immunobiological relationship between these synthetic oligosaccharides and RAW 264.7 cells was studied *in vitro*. Evaluation of cell proliferation, phagocytosis, respiratory burst, and Th1, Th2, Th17, and Treg polarized cytokine expression demonstrated effective immune responsiveness and immunomodulation in RAW 264.7 cells exposed to chitin- and chitosan-derived oligosaccharides. Macrophage reactivity was accompanied by significant inductive dose- and structure-dependent protective Th1 and Th17 polarization, which was greater with exposure to chitosan- rather than chitin-derived oligosaccharides. Moreover, no antiproliferative or cytotoxic effects were observed, even following prolonged 48 h exposure. The obtained results demonstrate the potent immunobiological activity of these synthetically prepared chito-oligosaccharides.

## Introduction

The fungal cell wall protects the cell from environmental stresses and is essential for cell morphogenesis and pathogenicity (Bowman and Free, 2006; Gow and Hube, 2012; Erwig and Gow, 2016; Gow et al., 2017). It comprises mostly polysaccharides of different types (Latgé, 2010) that account for ~90% of the cell wall, each carrying out a specific biological function. Chitin, a homopolymer of β-(1→4)-linked *N*-acetyl-d-glucosamine, is an important constituent of the innermost layer of the fungal cell wall and forms a covalent complex with β-(1→3)-glucan, which is responsible for the mechanical strength and integrity of the cell wall (Free, 2013). Chitin can undergo enzymatic N-deacetylation in the fungal cell wall by chitin deacetylases to form chitosan, a polymer of β-(1→4)-d-glucosamine or its copolymer with *N*-acetyl-d-glucosamine. The biological role of chitosan in the cell wall is not completely clear; however, research suggests that chitosan is important for morphogenesis (Geoghegan and Gurr, 2016; Upadhya et al., 2018), cell integrity (Baker et al., 2007), and virulence (Baker et al., 2011; Geoghegan and Gurr, 2016; Upadhya et al., 2018). Chitosan can be either prepared by chemical deacetylation of chitin or isolated from the cell walls of Zygomycota or other fungi, such as Basidiomycota, e.g., *Cryptococcus neoformans*. Cell wall polysaccharides are also engaged in the pathogen-associated molecular patterns associated with interactive crosstalk between immunocytes and immune sensing of the host organism, triggering various immune responses—innate and/or adaptive.

FIBCD1, NKR-P1, and RegIIIc have been identified as chitin-binding pattern recognition receptors in mammals. Other receptors that participate in the mediation of immune responses to chitin are Toll-like receptor (TLR) 2, dectin-1, and mannose receptor CD 206 (Bueter et al., 2013). Recently, Wagener et al. (2014) identified NOD2 and TLR9 as essential fungal chitin-recognition receptors engaged in chitin-induced selective secretion of IL-10. Studies by Da Silva and Kogiso (Da Silva et al., 2009; Kogiso et al., 2011) suggested the importance of chitin particle size on the character of the elicited immune response; large particles (>40 μm) reportedly induced a classical Th2 response, whereas small particles (1–10 μm) induced both protective Th1, and anti-inflammatory responses.

Chitosan has been shown to exert immunomodulative, anti-inflammatory, anti-cancer, anti-bacterial, and anti-fungal activities (Tsai et al., 2004; Kato et al., 2005; Yang et al., 2010; Tavaria et al., 2013; Bastiaens et al., 2019; de Carvalho et al., 2019). Chitosan has also been suggested as a promising adjuvant in new delivery systems due to its biocompatibility, biodegradability, and non-toxicity (Felt et al., 1998; Panos et al., 2008; Moran et al., 2018; Cunha et al., 2019). Chitosan-based hydrogels have been used for active drug delivery, gene delivery, and in various cancer therapies (Moran et al., 2018; Rahmani et al., 2019). The antimicrobial activity of chitin and chitin derivatives against bacteria, yeast, and fungi has been considered for various applications (Khoushab and Yamabhai, 2010).

Generally, immune sensing and recognition of fungi is cooperating with the interactive system of microbial-, pathogen-, and danger-associated molecular patterns (MAMPs, PAMPs, and DAMPs) engaged in interactions with soluble- and cell-associated pattern recognition receptors (PRRs). The synthetic glycans and oligosaccharides with well-defined composition are appropriate structures to characterize the minimal acquired immunobiologically active structures—referred as antigenic determinants or epitopes engaged in immune responses. Several synthetic immunogenic oligosaccharides partially mimicking the structure of fungal cell wall PAMP molecules, sensed by germ-line encoded PRRs and engaged in innate immune system cells signaling, have been designed and constructed (Xin et al., 2008; Costello and Bundle, 2012; Dang et al., 2012; Cartmell et al., 2015; Colombo et al., 2018 etc.). The immunobiological effectivity of the synthetically prepared oligosaccharides mimicking the natural fungal cell wall glycans demonstrated *in vitro* and *in vivo* the induction of effective cell proliferation, cell phagocytosis, T- and B-cell responses, macrophage secretion of Th1, Th2, Th17, and Treg inflammatory and anti-inflammatory interleukins, and growth factors (Paulovičová et al., 2013, 2014, 2015, 2016, 2017; Karelin et al., 2016).

Difficulties associated with isolation and purification, poor solubility, and possible irregularity of fungal polysaccharides themselves contribute to the inconvenience of studying their functions, immunological properties, and biological activities. Chemically synthesized regular polysaccharides might provide useful substitutes; however, polysaccharide synthesis is rather complex and time-consuming (Kochetkov et al., 1987). For this reason, synthetic oligosaccharides that are structurally related to fungal polysaccharides take on special significance for investigation of their immunological and other biological activities.

In particular, over the past few years, we have synthesized an array of biotinylated synthetic oligosaccharides representing fragments of fungal cell wall polysaccharides on streptavidin-coated surfaces (Krylov and Nifantiev, 2020). Such arrays have been applied to assess carbohydrate specificity of monoclonal antibodies (Matveev et al., 2018, 2019; Krylov et al., 2019; Schubert et al., 2019; Kazakova et al., 2020) and polyclonal antibodies in model sera induced by immunization of animals with cell wall preparations of various fungal species (Komarova et al., 2015; Paulovičová et al., 2016; Krylov et al., 2018a,b), as well as in sera obtained from patients with fungal infections (Komarova et al., 2018; Kazakova et al., 2020; Wong et al., 2020).

Here, we report the efficient synthesis of biotin-tagged chitosan oligomers composed of 3, 5, and 7 glucosamine units. Using synthesized chitosan oligomers, as well as previously prepared chitin counterparts (Yudina et al., 2016), we demonstrate the immunomodulative activities of synthetically prepared chitin- and chitosan-derived oligosaccharide formulas of various lengths and evaluate the structure–immunomodulation relationship.

## Materials and Methods

### Chemistry

#### General

Chemicals were purchased from Acros Organics (Geel, Belgium) and Sigma-Aldrich (St. Louis, MO, USA) and used without further purification. All moisture-sensitive reactions were carried out using dry solvents under dry argon. Solutions were concentrated under reduced pressure using a rotatory evaporator at 40°C (bath temperature).

NMR spectra were obtained using a Bruker Avance 600 MHz NMR spectrometer (Bruker, Billerica, MA, USA). Protected oligosaccharides were measured in chloroform-d (CDCl_3_), and ^1^H NMR chemical shifts were referenced to the solvent residual signal (δ_H_ 7.27). ^13^C chemical shifts were referenced to the central resonance of CDCl_3_ (δ_C_ 77.0). Free oligosaccharides were measured in deuterium oxide (D_2_O) using acetone (δ_H_ 2.225, δ_C_ 31.45) as an internal standard. Signal assignment was made using COZY, TOCSY, and HSQC experiments. Unit A refers to the reducing end monosaccharide in the description of NMR data. NMR spectra of synthesized compounds are presented in the **Supplementary Material**.

High-resolution mass spectrometry (HRMS) with electrospray ionization (ESI) was performed on a MicrOTOF II (Bruker Daltonics, Billerica, MA, USA) instrument.

Optical rotations were measured using a JASCO DIP-360 polarimeter at 18–22°C in the specified solvents.

TLC was performed on Silica Gel 60 F254 plates (Merck Millipore, Darmstadt, Germany), and visualization was accomplished using UV light or by charring at ~150°C with 10% (v/v) phosphoric acid in ethanol or Mostain reagent (ceric sulfate (1% w/v) and ammonium molybdate (2.5% w/v) in 10% (v/v) aqueous sulfuric acid).

Column chromatography was performed using Silica Gel 60 (40–63 μm; Merck Millipore). Gel-permeation chromatography of protected oligosaccharides was performed on a Bio-Beads S-X1 column (3.5 × 70 cm, column A; Bio-Rad Laboratories, Hercules, CA, USA) in toluene. Gel-permeation chromatography of free oligosaccharides and biotin-tagged derivatives was performed on Toyopearl TSK HW-40(s) columns (2.8 × 80 cm and 2.0 × 35 cm; columns B and C, respectively) in 0.1 M acetic acid. A K-2401 refractive index detector (Knauer, Berlin, Germany) was used to monitor gel-permeation chromatography.

#### Synthesis of Biotinylated Chitosan Oligosaccharides

##### Ethyl 3, 6-di-O-benzyl-4-O-(tert-butyldimethylsilyl)-2-deoxy-2-phthalimido-1-thio-β-d-glucopyranoside(**4**)

2,4,6-Collidine (0.945 ml, 7.18 mmol) and *tert*-butyldimethylsilyl triflate (1.19 ml, 5.17 mmol) were added to a solution of thioglycoside 2 (1.53 g, 2.87 mmol) in dichloromethane (30 ml), and the resulting mixture was stirred at room temperature (RT) for 2 h. Methanol (100 μl) was added, and the mixture was diluted with chloroform (70 ml) and washed successively with aqueous 1 M hydrochloric acid, water, and saturated aqueous sodium bicarbonate. The solvents were then evaporated, and the residue was purified by column chromatography (petroleum ether/ethyl acetate 9:1) to produce silyl ether **4** (1.837 g, 99%) as a colorless foam, [α]_D_ +76 (*c* 2, chloroform). ^1^H NMR (600 MHz, CDCl_3_): δ 7.74–7.63 (m, 4 H, Ar), 7.40–7.27 (m, 5 H, Ar), 7.02–6.83 (m, 5 H, Ar), 5.31–5.26 (m, 1 H, H-1), 4.79 (d, 1 H, *J* = 12.3 Hz, PhC*Ha*Hb), 4.69 (d, 1 H, *J* = 12.3 Hz, PhC*Ha*Hb′), 4.57 (d, 1 H, *J* = 12.3 Hz, PhCHa*Hb*), 4.32 (d, 1 H, *J* = 12.3 Hz, PhCHa*Hb*′), 4.30–4.24 (m, 2 H, H-2, H-3), 3.81 (br. d, 1 H, *J*_6a,6b_ = 9.7 Hz, H-6a), 3.76–3.72 (m, 1 H, H-4), 3.69–3.62 (m, 2 H, H-5, H-6b), 2.74–2.67 (m, 1 H, SC*Ha*HbCH_3_), 2.65–2.58 (m, 1 H, SCHa*Hb*CH_3_), 1.20 (t, 3 H, *J* = 7.6 Hz, SCH_2_C*H*_3_), 0.89 (s, 9 H, SiC(CH_3_)_3_), 0.09, 0.07 (2 s, 6 H, 2 SiCH_3_). ^13^C NMR (150.9 MHz, CDCl_3_): δ 168.3, 167.4 (2 CO), 138.4, 138.2, 133.7, 133.6, 131.6, 128.3, 127.9, 127.5, 127.4, 127.2, 127.0, 123.4, 123.1 (Ar), 82.0 (C-3), 81.0 (C-1), 80.7 (C-5), 75.4 (Ph*C*H_2_), 73.3 (Ph*C*H_2_), 72.6 (C-4), 69.3 (C-6), 55.1 (C-2), 24.9 (SiC(*C*H_3_)_3_), 23.9 (S*C*H_2_CH_3_), 15.0 (SCH_2_*C*H_3_), −3.8, −4.5 (2 SiCH_3_). HRMS (ESI) *m/z* found: 670.2626 [M + Na]^+^; calcd. for C_36_H_45_NO_6_SSi: 670.2629.

##### 3, 6-Di-O-benzyl-4-O-(tert-butyldimethylsilyl)-2-deoxy-2-phthalimido-β-d-glucopyranose(**5**)

N-bromosuccinimide (2.46 g, 13.8 mmol) was added to a solution of thioglycoside **4** (1.79 g, 2.76 mmol) in a mixture of acetone (40 ml) and water (3.5 ml) at −15°C. The mixture was stirred at that temperature for 20 min, diluted with chloroform (250 ml), and washed with a 1:1 mixture of aqueous 1 M sodium thiosulfate and saturated aqueous sodium bicarbonate. The organic layer was separated and taken to dryness. The residue was then subjected to column chromatography (toluene/ethyl acetate 4:1) to produce hemiacetal **5** (1.50 g, 90%) as a colorless foam, [α]_D_ +88 (*c* 2, chloroform). ^1^H NMR (600 MHz, CDCl_3_):δ 7.69–7.58 (m, 4 H, Ar), 7.36–7.15 (m, 5 H, Ar), 7.01–6.79 (m, 5 H, Ar), 5.40 (dd, 1 H, *J*_1,2_ = 8.4 Hz, *J*_1,OH_ = 7.2 Hz, H-1β), 5.30 (t, 0.04 H, *J* = 3.9 Hz, H-1α), 4.77 (d, 1 H, *J* = 12.3 Hz, PhC*Ha*Hb), 4.66 (d, 1 H, *J* = 12.3 Hz, PhC*Ha*Hb′), 4.49 (d, 1 H, *J* = 12.3 Hz, PhCHa*Hb*′), 4.30 (d, 1 H, *J* = 12.3 Hz, PhCHa*Hb*), 4.26 (dd, 1 H, *J*_3,4_ = 7.9 Hz, H-3), 4.10 (dd, 1 H, *J*_2,3_ = 10.8 Hz, H-2), 3.74 (br. d, 1 H, *J*_6a,6b_ = 10.3 Hz, H-6a), 3.72–3.66 (m, 2 H, H-4, H-5), 3.58–3.53 (m, 1 H, H-6b), 0.85 (s, 9 H, SiC(CH_3_)_3_), 0.07, and 0.00 (2 s, 6 H, 2 SiCH_3_). ^13^C NMR (150.9 MHz, CDCl_3_): δ 168.2 (CO), 138.2, 137.9, 134.0, 133.6, 131.6, 128.4, 127.9, 127.8, 127.6, 127.2, 127.0, 123.2 (Ar), 92.7 (C-1), 80.6 C-3), 76.0 (C-5), 75.3 (Ph*C*H_2_), 73.3 (Ph*C*H_2_), 72.7 (C-4), 69.1 (C-6), 57.5 (C-2), 25.8 (SiC(*C*H_3_)_3_), −3.8, −4.6 (2 SiCH_3_). HRMS (ESI) *m/z* found: 626.2542 [M + Na]^+^; calcd. for C_34_H_41_NO_7_Si: 626.2545.

##### O-[3, 6-Di-O-benzyl-4-O-(tert-butyldimethylsilyl)-2-deoxy-2-phthalimido-β-d-glucopyranosyl]trichloroacetimidate (**6**)

Trichloroacetonitrile (2.33 ml, 23.2 mmol) and 2,3,4,6,7,8,9,10-octahydropyrimido[1,2-a]azepine (DBU; 34 μl, 0.23 mmol) were added to a solution of hemiacetal **5** (1.40 g, 2.32 mmol) in dichloromethane (20 ml), and the mixture was stirred for 1.5 h at RT. The solvent was evaporated, and the residue was purified by column chromatography (petroleum ether/ethyl acetate 85:15 + 0.2% (v/v) triethylamine) to give imidate **6** (1.58 g, 91%) as a colorless foam, [α]_D_ +89 (*c* 1, chloroform). ^1^H NMR (600 MHz, CDCl_3_): δ 8.54 (s, 1 H, NH), 7.68–7.60 (m, 4 H, Ar), 7.40–7.25 (m, 5 H, Ar), 7.03–6.80 (m, 5 H, Ar), 6.44 (d, 1 H, *J*_1,2_ = 8.9 Hz, H-1), 4.83 (d, 1 H, *J* = 12.4 Hz, PhC*Ha*Hb), 4.72 (d, 1 H *J* = 12.4 Hz, PhC*Ha*Hb′), 4.59 (d, 1 H, *J* = 12.4 Hz, PhCHa*Hb*), 4.49 (dd, 1 H, *J*_2,3_ = 10.8 Hz, H-2), 4.40–4.34 (m, 2 H, PhCHa*Hb*′, H-3), 3.91–3.81 (m, 3 H, H-4, H-5, H-6a), 3.72 (dd, 1 H, *J*_6b,5_ = 5.6 Hz, *J*_6a,6b_ = 11.6 Hz), 0.91 (s, 9 H, SiC(CH_3_)_3_), 0.12, 0.10 (2 s, 6 H, 2 SiCH_3_). ^13^C NMR (150.9 MHz, CDCl_3_): δ 167.7 (CO), 160.8 (CNH), 138.3, 138.2, 133.8131.5, 128.3, 128.0, 127.7, 123.2 (Ar), 94.1 (C-1), 80.8 (C-3), 77.4 (C-5), 75.5 (Ph*C*H_2_), 73.2 (Ph*C*H_2_), 72.2 (C-4), 68.4 (C-6), 55.0 (C-2), 25.9 (SiC(*C*H_3_)_3_), −3.8, −4.5 (2 SiCH_3_). HRMS (ESI) *m/z* found: 769.1630 [M + Na]^+^; calcd. for C_36_H_41_Cl_3_N_2_O_7_Si: 769.1641.

##### Ethyl3, 6-di-O-benzyl-4-O-(tert-butyldimethylsilyl) -2-deoxy-2-phthalimido-β-d-glucopyranosyl-(1→4)-3,6-di-O-benzyl-2-deoxy-2-phthalimido-1-thio-β-d-glucopyranoside (**7**)

A mixture of imidate **6** (1.42 g, 1.89 mmol) and thioglycoside acceptor **2** (840 mg, 1.57 mmol) was dried by co-evaporation with anhydrous toluene (2 × 15 ml), dried under vacuum for 2 h, and dissolved in dichloromethane (30 ml). Powdered molecular sieve 4 Å (3 g) was added to the solution, and the mixture was stirred for 1.5 h at RT and then cooled to −78°C. Trimethylsilyl trifluoromethanesulfonate (TMSOTf; 34 μl, 0.19 mmol) was added, and the mixture was stirred at −78°C for 45 min. The reaction was then quenched with triethylamine (50 μl), and the solids were filtered off through Celite and washed with chloroform. The filtrate was then washed with water and concentrated. Column chromatography of the residue (petroleum ether/ethyl acetate 4:1→3:1) produced disaccharide **7** (1.55 g, 88%) as a colorless foam, [α]_D_ +33 (*c* 2, chloroform). ^1^H NMR (600 MHz, CDCl_3_): δ 7.83–7.55 (m, 8 H, Ar), 7.39–7.25 (m, 10 H, Ar), 7.03–6.79 (m, 10 H. Ar), 5.33 (d, 1 H, *J*_1,2_ = 7.8 Hz, H-1^B^), 5.08 (d, 1 H, *J*_1,2_ = 10.2 Hz, H-1^A^), 4.88 (d, 1 H, *J* = 12.7 Hz, PhC*Ha*Hb), 4.83 (d, 1 H, *J* = 12.2 Hz, PhC*Ha*Hb′), 4.70 (d, 1 H, *J* = 12.4 Hz, PhC*Ha*Hb″), 4.56 (d, 1 H, *J* = 12.7 Hz, PhCHa*Hb*), 4.55 (d, 1 H, *J* = 11.8 Hz, PhC*Ha*Hb^‴^), 4.51 (d, 1 H, *J* = 12.4 Hz, PhCHa*Hb*″), 4.50 (d, 1 H, *J* = 11.8 Hz, PhCHa*Hb*^‴^), 4.33 (d, 1 H, *J* = 12.4 Hz, PhCHa*Hb*″), 4.28–4.18 (m, 5 H, H-2^A^, H-3^A^, H-4^A^, H-2^B^, H-3^B^), 3.82 (dd, 1 H, *J*_4,3_ = 7.7 Hz, *J*_4,5_ = 9.6 Hz, H-4^B^), 3.72 (dd, 1 H, *J*_6a,5_ = 2.0 Hz, *J*_6a,6b_ = 11.0, H-6a^B^), 3.57 (br. d, 1 H, *J*_6a,6b_ = 11.1 Hz, H-6a^A^), 3.51 (dd, 1 H, *J*_6b,5_ = 4.8 Hz, H-6b^B^), 3.44 (dd, 1 H, *J*_6a,6b_ = 4.1 Hz, H-6b^A^), 3.37–3.33 (m, 2 H, H-5^A^, H-5^B^), 2.63–2.49 (m, 2 H, SC*H*_2_CH_3_), 1.12 (t, 3 H, *J* = 7.5 Hz, SCH_2_C*H*_3_), 0.93 (s, 9 H, SiC(CH_3_)_3_), 0.10, 0.09 (2 s, 6 H, 2 SiCH_3_). ^13^C NMR (150.9 MHz, CDCl_3_): δ 168.8, 167.8, 167.4 (CO), 138.7, 138.4, 133.8, 133.6, 133.5, 128.3, 128.2, 127.9, 127.7, 127.5, 127.4, 127.3, 127.2, 127.0, 126.8, 123.6, 123.3, 123.1 (Ar), 96.7 (C-1^B^), 81.22 (C-3^B^), 80.8 (C-1^A^), 79.0 (C-5^A^), 77.6 (C-3^A^), 76.5 (C-5^B^), 75.4, 75.3 (C-4^A^, Ph*C*H_2_), 74.4 (Ph*C*H_2_), 73.1 (Ph*C*H_2_), 72.8 (Ph*C*H_2_), 72.4 (C-4^B^), 68.5 (C-6^A^), 68.2 (C-6^B^), 56.8 (C-2^B^), 54.8 (C-2^A^), 26.0 (SiC(*C*H_3_)_3_), 23.7 (S*C*H_2_CH_3_), 14.9 (SCH_2_*C*H_3_), −3.8, −4.6 (2 SiCH_3_). HRMS (ESI) *m/z* found: 1,136.4741 [M + NH_4_]^+^; calcd. for C_64_H_70_N_2_O_12_SSi: 1,136.4757.

##### 2-Azidoethyl3, 6-di-O-benzyl-4-O-(tert-butyldimethylsilyl) -2-deoxy-2-phthalimido-β-d-glucopyranosyl-(1→4)-3,6-di-O-benzyl-2-deoxy-2-phthalimido-β-d-glucopyranosyl-(1→4)-3,6-di-O-benzyl-2-deoxy-2-phthalimido-β-d-glucopyranoside (**9**)

A mixture of thioglycoside **7** (970 mg, 0.87 mmol) and acceptor **8** (440 mg, 0.79 mmol) was dried by co-evaporation with anhydrous toluene (2 × 15 ml) and dried under vacuum for 1 h. After dissolution in dichloromethane (20 ml), powdered molecular sieve 4 Å (1.5 g) was added, and the mixture was stirred for 2 h at RT, followed by cooling to −40°C. N-iodosuccinimide (254 mg, 1.13 mmol) was added, and the mixture was stirred for 15 min at that temperature. After cooling to −50°C, trifluoromethanesulfonic acid (TfOH) (10 μl, 0.11 mmol) was added, and the mixture was stirred for 1 h. The reaction was quenched with pyridine (25 μl), and the solids were filtered off through Celite and washed with chloroform. The filtrate was then washed with aqueous 1 M sodium thiosulfate and water and then concentrated. Column chromatography (toluene/ethyl acetate 93:7→88:12) of the residue produced trisaccharide **9** (1.128 g, 89%) as a white foam, [α]_D_ +29 (*c* 2, chloroform). ^1^H NMR (600 MHz, CDCl_3_): δ 7.88–7.57 (m, 12 H, Ar), 7.34–6.70 (m, 30 H, Ar), 5.31 (d, 1 H, *J*_1,2_ = 7.8 Hz, H-1^C^), 5.14–5.09 (m, 1 H, H-1^B^), 5.03–4.99 (m, 1 H, H-1^A^), 4.92 (d, 1 H, *J* = 12.8 Hz, benzylic H), 4.82 (d, 1 H, *J* = 12.2 Hz, benzylic H), 4.79 (d, 1 H, *J* = 12.8 Hz, benzylic H), 4.64 (d, 1 H, *J* = 12.2 Hz, benzylic H), 4.55 (d, 1 H, *J* = 12.2 Hz, benzylic H), 4.54 (d, 1 H, *J* = 12.8 Hz, benzylic H), 4.49–4.38 (m, 5 H, benzylic H), 4.32 (d, 1 H, *J* = 12.2 Hz, benzylic H), 4.28–4.21 (m, 3 H, H-4^B^, H-2^C^, H-3^C^), 4.19–4.14 (m, 2 H, H-2^B^, H-3^B^), 4.13–4.03 (m, 3 H, H-2^A^, H-3^A^, H-4^A^), 3.83–3.79 (m, 2 H, H-4^C^, OC*Ha*CHbCH_2_N_3_), 3.69 (dd, 1 H, *J*_6a,5_ = 1.8 Hz, *J*_6a,6b_ = 11.0 Hz, H-6a^C^), 3.49–3.42 (m, 4 H, H-6a^A^, H-6a^B^, H-6b^C^, OCHaC*Hb*CH_2_N_3_), 3.37–3.23 (m, 4 H, H-5^A^, H-5^C^, H-6b^A^, OCH_2_C*Ha*HbN_3_), 3.20 (dd, 1 H, *J*_6b,5_ = 3.3 Hz, *J*_6a,6b_ = 11.1 Hz, H-6b^B^), 3.05 (m, 1 H, OCH_2_CHa*Hb*N_3_), 2.96 (br. d, 1 H, *J* = 10.1 Hz, H-5^B^), 0.92 (s, 9 H, SiC(CH_3_)_3_), 0.08 (s, 6 H, 2 SiCH_3_). ^13^C NMR (150.9 MHz, CDCl_3_): δ 168.7, 168.2, 167.5 (CO), 139.0, 138.6, 138.5, 138.4, 138.2, 133.8, 133.6, 133.4, 128.3, 128.2, 128.1, 128.0, 127.9, 127.7, 127.4, 127.2, 127.1, 127.0, 126.8, 123.6, 123.5, 123.1 (Ar), 98.2 (C-1^A^), 96.9 (C-1^B^), 96.7 (C-1^C^), 81.2 (C-3^C^), 76.8 (C-3^B^), 76.7 (C-3^A^), 76.4 (C-5^C^), 75.7 (C-4^A^), 75.0 (C-4^B^), 75.3 (Ph*C*H_2_), 74.6 (C-5^A^), 74.5 (C-5^B^), 74.4 (2 C, 2 Ph*C*H_2_), 73.1 (Ph*C*H_2_), 72.6 (Ph*C*H_2_), 72.4 (Ph*C*H_2_), 72.3 (C-4^C^), 68.2 (2 C, C-6^A^, C-6^C^), 67.9 (O*C*H_2_CH_2_N_3_), 67.2 (C-6^B^), 56.8 (C-2^C^), 56.6 (C-2^B^), 55.5 (C-2^A^), 50.3 (OCH_2_*C*H_2_N_3_), 26.0 (SiC(*C*H_3_)_3_), −3.8, −4.6 (2 SiCH_3_). HRMS (ESI) *m/z* found: 1,632.6662 [M + NH_4_]^+^; calcd. for C_92_H_92_N_6_O_19_Si: 1,632.6681.

##### 2-Azidoethyl3, 6-di-O-benzyl-2-deoxy-2-phthalimido-β-d-glucopyranosyl-(1→4)-3,6-di-O-benzyl-2-deoxy-2-phthalimido-β-d-glucopyranosyl-(1→4)-3,6-di-O-benzyl-2-deoxy-2-phthalimido-β-d-glucopyranoside (**10**)

A solution of silyl ether **9** (1.066 g, 0.66 mmol) in acetonitrile (15 ml) was placed in a polypropylene screw-cap centrifuge tube. Aqueous 40% hydrofluoric acid (1.5 ml) was added, and the resulting solution was stirred for 48 h at RT. The mixture was diluted with chloroform (150 ml) and washed with water and saturated aqueous sodium bicarbonate, and the solvent was evaporated. Column chromatography of the residue (toluene/ethyl acetate 9:1→85:15) produced 4-OH derivative **10** (892 mg, 90%) as a colorless foam, [α]_D_ +13 (*c* 1, chloroform). ^1^H NMR (600 MHz, CDCl_3_): δ 7.96–6.67 (m, 42 H, Ar), 5.30 (d, 1 H, *J*_1,2_ = 8.4 Hz, H-1^C^), 5.13–5.08 (m, 1 H, H-1^B^), 5.01–4.98 (m, 1 H, H-1^A^), 4.83 (d, 1 H, *J* = 12.5 Hz, benzylic H), 4.80 (d, 1 H, *J* = 12.1 Hz, benzylic H), 4.78 (d, 1 H, *J* = 12.8 Hz, benzylic H), 4.57–4.38 (m, 9 H, benzylic H), 4.27 (dd, 1 H, *J*_2,3_ = 10.8 Hz, H-3^C^), 4.23–4.19 (m, 1 H, H-4^B^), 4.18–4.13 (m, 3 H, H-2^B^, H-3^B^, H-2^C^), 4.10–4.06 (m, 3 H, H-2^A^, H-3^A^, H-4^A^), 3.83–3.78 (m, 2 H, H-4^C^, OC*Ha*CHbCH_2_N_3_), 3.68 (dd, 1 H, *J*_6a,5_ = 4.2 Hz, *J*_6a,6b_ = 9.9 Hz, H-6a^C^), 3.52–3.46 (m, 2 H, H-6b^C^, H-6a^A^), 3.45–3.40 (m, 2 H, H-6a^B^, OCHaC*Hb*CH_2_N_3_), 3.40–3.37 (m, 1 H, H-5^C^), 3.34 (dd, 1 H, *J*_6b,5_ = 3.9 Hz, *J*_6a,6b_ = 10.8 Hz, H-6b^A^), 3.30–3.22 (m, 2 H, H-5^A^, OCH_2_C*Ha*HbN_3_), 3.18 (dd, 1 H, *J*_6b,5_ = 2.8 Hz, *J*_6a,6b_ = 11.0 Hz, H-6b^B^), 3.14 (br. s, 1 H, OH), 3.06–3.01 (m, 1 H, OCH_2_CHa*Hb*N_3_), 2.91 (br. d, 1 H, *J* = 10.1 Hz, H-5^B^). ^13^C NMR (150.9 MHz, CDCl_3_): δ 168.5, 167.9, 167.8 (CO), 139.0, 138.8, 138.6, 138.5, 138.4, 137.7, 134.3, 134.1, 134.0, 133.7, 132.0, 131.8, 128.7, 128.5, 128.4, 128.3, 128.2, 128.0, 127.9, 127.6, 127.5, 127.4, 127.3, 127.1, 127.0, 123.8, 123.5, 123.3 (Ar), 98.1 (C-1^A^), 96.9 (C-1^C^), 96.7 (C-1^B^), 78.3 (C-3^C^), 76.7 (C-3^B^), 76.6 (C-3^A^), 75.6 (C-4^C^), 75.5, 75.4 (C-4^A^, C-4^B^), 74.6 (C-5^A^), 74.3 (4 C, C-5^B^, 3 Ph*C*H_2_), 73.6 (Ph*C*H_2_), 72.6, 72.5 (C-5^C^, Ph*C*H_2_), 72.3 (Ph*C*H_2_), 71.7 (C-6^C^), 68.1 (C-6^A^), 67.9 (O*C*H_2_CH_2_N_3_), 67.1 (C-6^B^), 56.5 (C-2^B^), 56.0 (C-2^C^), 55.4 (C-2^A^), 50.2 (OCH_2_*C*H_2_N_3_). HRMS (ESI) *m/z* found: 1,523.5368 [M + Na]^+^; calcd. for C_86_H_80_N_6_O_19_: 1,523.5370.

##### 2-Azidoethyl3, 6-di-O-benzyl-4-O-(tert-butyldimethylsilyl)-2-deoxy-2-phthalimido-β-d-glucopyranosyl-(1→4)-3,6-di-O-benzyl-2-deoxy-2-phthalimido-β-d-glucopyranosyl-(1→4)-3,6-di-O-benzyl-2-deoxy-2-phthalimido-β-d-glucopyranosyl-(1→4)-3,6-di-O-benzyl-2-deoxy-2-phthalimido-β-d-glucopyranosyl-(1→4)-3,6-di-O-benzyl-2-deoxy-2-phthalimido-β-d-glucopyranoside (**11**)

A mixture of thioglycoside **7** (453 mg, 0.405 mmol) and trisaccharide acceptor **10** (508 mg, 0.338 mmol) was dried by co-evaporation with anhydrous toluene (2 × 15 ml) and dried under vacuum for 2 h. After dissolution in dichloromethane (12 ml), powdered molecular sieve 4 Å (1 g) was added, and the mixture was stirred for 1 h at RT and then cooled to −40°C. N-iodosuccinimide (137 mg, 0.61 mmol) was added, and the mixture was stirred for 15 min at that temperature. After cooling to −50°C, TfOH (5 μl, 0.057 mmol) was added, and the mixture was stirred for 1 h while the temperature was gradually increased to −40°C. The reaction was quenched with pyridine (100 μl); the solids were filtered off through Celite, followed by washing with chloroform. The filtrate was washed with aqueous 1 M sodium thiosulfate and water and then concentrated. The residue was subjected to gel chromatography on column A in toluene. Fractions containing pentasaccharide **11** were pooled and concentrated, and the residue was further purified by column chromatography (toluene/ethyl acetate 9:1→85:15) to give pentasaccharide **11** (648 mg, 75%) as a colorless foam, [α]_D_ +24 (*c* 1, chloroform). ^1^H NMR (600 MHz, CDCl_3_): δ 7.88–6.64 (m, 70 H, Ar), 5.26 (d, 1 H, *J*_1,2_ = 7.9 Hz, H-1^E^), 5.07–5.01 (m, 3 H, 3 H-1), 4.98–4.95 (m, 1 H, H-1^A^), 4.89 (d, 1 H, *J* = 13.0 Hz, benzylic H), 4.81 (d, 1 H, *J* = 12.8 Hz, benzylic H), 4.79 (d, 1 H, *J* = 12.3 Hz, benzylic H), 4.76 (d, 1 H, *J* = 13.0 Hz, benzylic H), 4.71 (d, 1 H, *J* = 12.8 Hz, benzylic H), 4.61 (d, 1 H, *J* = 12.3 Hz, benzylic H), 4.50 (d, 1 H, *J* = 13.0 Hz, benzylic H), 4.49 (d, 1 H, *J* = 11.7 Hz, benzylic H), 4.45–3.99 (m, 26 H, 5 H-2, 5 H-3, 4 H-4, 12 benzylic H), 3.81–3.76 (m, 2 H, H-4^E^, OC*Ha*CHbCH_2_N_3_), 3.65 (br. d, *J*_6a,6b_ = 10.6 Hz, H-6a^E^), 3.46–3.20 (m, 10 H, H-5^A^, H-5^E^, 6 H-6, OCHaC*Hb*CH_2_N_3_, OCH_2_C*Ha*HbN_3_), 3.12 (dd, 1 H, *J*_6a,6b_ = 10.8 Hz, *J*_6,5_ = 2.9 Hz, H-6), 3.06–2.99 (m, 3 H, 2 H-6, OCH_2_CHa*Hb*N_3_), 2.86 (br. d, 1 H, *J* = 9.9 Hz, H-5), 2.80 (m, 2 H, 2 H-5), 0.89 (s, 9 H, SiC(CH_3_)_3_), 0.06 (s, 6 H, 2 SiCH_3_). ^13^C NMR (150.9 MHz, CDCl_3_): δ 168.6, 168.0, 167.6, 167.5 (CO), 138.9, 138.8, 138.6, 138.5, 138.4, 138.3, 138.2, 138.1, 133.8, 133.7, 133.5, 133.4, 131.8, 131.6, 131.5, 128.2, 128.0, 127.9, 127.8, 127.7, 127.6, 127.3, 127.2, 127.1, 126.9, 126.8, 126.7, 126.5, 123.5, 123.4, 123.3, 123.1, 123.0 (Ar), 98.1 (C-1^A^), 96.7, 96.6 (2 C), 96.5 (4 C-1), 81.2 (C-3^E^), 76.9, 76.7, 76.5, 76.3 (4 C-3, C-5^E^), 75.4, 75.3 (3 C), 75.1 (4 C-4, Ph*C*H_2_), 74.6, 74.5, 74.4 (2 C), 74.2 (2 C), 74.1 (4 C-5, 4 Ph*C*H_2_), 73.0 (Ph*C*H_2_), 72.5, 72.3, 72.2, 72.1 (2 C) (C-4^E^, 4 Ph*C*H_2_), 68.0 (2 C), 67.9, 67.2, 67.0 (2 C) (5 C-6, O*C*H_2_CH_2_N_3_), 56.7, 56.6 (3 C), 55.4 (5 C-2), 50.2 (OCH_2_*C*H_2_N_3_), 25.9 (SiC(*C*H_3_)_3_), −3.9, −4.6 (2 SiCH_3_). HRMS (ESI) *m/z* found: 2,579.9595 [M + Na]^+^; calcd. for C_148_H_144_N_8_O_31_Si: 2,579.9599.

##### 2-Azidoethyl3, 6-di-O-benzyl-2-deoxy-2-phthalimido-β -d-glucopyranosyl-(1→4)-3,6-di-O-benzyl-2-deoxy-2-phthalimido-β-d-glucopyranosyl-(1→4)-3,6-di-O-benzyl-2-deoxy-2-phthalimido-β-d-glucopyranosyl-(1→4)-3,6-di-O-benzyl-2-deoxy-2-phthalimido-β-d-glucopyranosyl-(1→4)-3,6-di-O-benzyl-2-deoxy-2-phthalimido-β-d-glucopyranoside (**12**)

Aqueous 40% hydrofluoric acid (1 ml) was added to a solution of silyl ether **11** (648 mg, 0.253 mmol) in acetonitrile (10 ml). Dichloromethane was added until the turbid solution became clear (~1 ml). The mixture was stirred for 72 h and worked up as described above for trisaccharide **10**. Purification by column chromatography (toluene/ethyl acetate 9:1→3:1) produced 4-OH derivative **12** (582 mg, 94%) as a colorless foam, [α]_D_ +12 (*c* 1, chloroform). ^1^H NMR (600 MHz, CDCl_3_): δ 7.93–6.64 (m, 70 H, Ar), 5.28 (d, 1 H, *J*_1,2_ = 8.5 Hz, H-1^E^), 5.07 (d, 1 H, *J*_1,2_ = 7.6 Hz, H-1), 5.05–5.02 (m, 2 H, 2 H-1), 4.98–4.94 (m, 1 H, H-1^A^), 4.82 (d, 2 H, *J* = 12.7 Hz, 2 benzylic H), 4.79 (d, 1 H, *J* = 12.5 Hz, benzylic H), 4.77 (d, 1 H, *J* = 12.9 Hz, benzylic H), 4.72 (d, H, *J* = 13.1 Hz, benzylic H), 4.52 (d, H, *J* = 12.3 Hz, benzylic H), 4.50 (d, H, *J* = 11.8 Hz, benzylic H), 4.46–4.33 (m, 11 H, 11 benzylic H), 4.29–4.23 (m, 3 H, H-3^E^, 2 benzylic H), 4.21–3.99 (m, 13 H, 5 H-2, 4 H-3, 4 H-4), 3.81–3.76 (m, 2 H, H-4^E^, OC*Ha*HbCH_2_N_3_), 3.67 (dd, 1 H, *J*_6a,5_ = 4.2 Hz, *J*_6a,6b_ = 9.7 Hz, H-6a^E^), 4.48 (dd, 1 H, *J*_6b,5_ = 6.3 Hz, H-6b^E^), 3.45–3.28 (m, 7 H, H-5^E^, 5 H-6, OCHa*Hb*CH_2_N_3_), 3.26–3.20 (m, 2 H, H-5, OCH_2_C*Ha*HbN_3_), 3.14 (dd, 1 H, *J*_6,5_ = 2.5 Hz, *J*_6a,6b_ = 11.0 Hz, H-6), 3.11 (d, 1 H, *J*_OH,4_ = 1.5 Hz, OH), 3.06–3.00 (m, 3 H, 2 H-6, OCH_2_CHa*Hb*N_3_), 2.87–2.79 (m, 3 H, 3 H-5). ^13^C NMR (150.9 MHz, CDCl_3_): δ 168.2, 168.1, 168.0, 167.6, 167.5 (CO), 138.8, 138.5, 138.4, 138.3, 138.2, 135.7, 133.9, 133.8, 133.7, 133.6, 133.5, 133.4, 131.8, 131.6, 131.5, 128.4, 128.2, 128.0, 127.9, 127.8, 127.7, 127.6, 127.3, 127.2, 127.1, 127.0, 126.9, 126.8, 126.7, 123.5, 123.3, 123.1, 123.0 (Ar), 98.1 (C-1^A^), 96.8, 96.7, 96.6 (2 C) (4 C-1), 78.3 (C-3^E^), 76.9, 76.8 (2 C), 76.6 (4 C-3), 75.6 (C-4^E^), 75.5, 75.4 75.3 (2 C) (4 C-4), 74.6 (C-5^A^), 74.5, 74.4, 74.3 (4 C), 74.2 (2 C) (3 C-5, 5 Ph*C*H_2_), 73.7 (Ph*C*H_2_), 72.6, 72.5, 72.3, 72.2 (2 C) (C-5^E^, 4 Ph*C*H_2_), 71.1 (C-6^E^), 68.1, 67.8, 67.2 (2 C), 67.1 (4 C-6, O*C*H_2_CH_2_N_3_), 56.6 (3 C), 56.1, 55.4 (5 C-2), 50.3 (OCH_2_*C*H_2_N_3_). HRMS (ESI) *m/z* found: 2,460.9193 [M + NH_4_]^+^; calcd. for C_142_H_130_N_8_O_31_: 2,460.9180.

##### 2-Azidoethyl3, 6-di-O-benzyl-4-O-(tert-butyldimethylsilyl) -2-deoxy-2-phthalimido-β-d-glucopyranosyl-(1→4)-3,6-di-O-benzyl-2-deoxy-2-phthalimido-β-d-glucopyranosyl-(1→4)-3,6-di-O-benzyl-2-deoxy-2-phthalimido-β-d-glucopyranosyl-(1→4)-3,6-di-O-benzyl-2-deoxy-2-phthalimido-β-d-glucopyranosyl-(1→4)-3,6-di-O-benzyl-2-deoxy-2-phthalimido-β-d-glucopyranosyl-(1→4)-3,6-di-O-benzyl-2-deoxy-2-phthalimido-β-d-glucopyranosyl-(1→4)-3,6-di-O-benzyl-2-deoxy-2-phthalimido-β-d-glucopyranoside (**13**)

A mixture of disaccharide thioglycoside **7** (148 mg, 0.132 mmol) and pentasaccharide acceptor **12** (269 mg, 0.11 mmol) was dried by co-evaporation with anhydrous toluene (2 × 10 ml) and dried under vacuum for 2 h. After dissolution in dichloromethane (12 ml), powdered molecular sieve 4 Å (500 g) was added, and the mixture was stirred for 2 h at RT and cooled to −40°C. N-iodosuccinimide (45 mg, 0.20 mmol) was added, and the mixture was stirred for 20 min at that temperature. After cooling to −50°C, TfOH (1.8 μl, 0.02 mmol) was added, and the mixture was stirred for 2 h while the temperature was gradually increased to −40°C. The reaction was quenched with pyridine (10 μl), and the solids were filtered off through Celite and washed with chloroform. The filtrate was washed with aqueous 1 M sodium thiosulfate and water and then concentrated. The residue was subjected to gel chromatography on column A in toluene. Fractions containing the heptasaccharide were pooled and concentrated, and the residue was further purified by column chromatography (toluene/ethyl acetate 85:15→4:1) to give heptasaccharide **13** (250 mg, 65%) as a colorless foam, [α]_D_ +20 (*c* 1, chloroform). ^1^H NMR (600 MHz, CDCl_3_): δ 7.87–6.63 (m, 98 H, Ar), 5.26 (d, 1 H, *J*_1,2_ = 7.8 Hz, H-1^G^), 5.05 (d, 1 H, *J*_1,2_ = 7.6 Hz, H-1), 5.03–4.98 (m, 4 H, 4 H-1), 4.97–4.95 (m, 1 H, H-1^A^), 4.89 (d, 1 H, *J* = 12.9 Hz, benzylic H), 4.81–4.69 (m, 7 H, 7 benzylic H), 4.61 (d, 1 H, *J* = 12.3 Hz, benzylic H), 4.49 (d, 1 H, *J* = 12.9 Hz, benzylic H), 4.48 (d, 1 H, *J* = 11.6 Hz, benzylic H), 4.43–3.98 (m, 37 H, 7 H-2, 7 H-3, 6 H-4, 17 benzylic H), 3.80–3.75 (m, 2 H, H-4^G^, OC*Ha*CHbCH_2_N_3_), 3.64 (br. d, 1 H, *J*_6a,6b_ = 10.1 Hz, H-6a^G^), 3.46–3.39 (m, 3 H, H-6b^G^, H-6a, OCHaC*Hb*CH_2_N_3_), 3.36 (br. d, 1 H, *J*_6a,6b_ = 10.1 Hz, H-6a), 3.32–3.20 (m, 8 H, 2 H-5, 5 H-6, OCH_2_C*Ha*HbN_3_), 3.12 (br. d, 1 H, *J*_6a,6b_ = 10.1 Hz, H-6b), 3.04–2.97 (m, 5 H, 4 H-6, OCH_2_CHa*Hb*N_3_), 2.86 (br. d, 1 H, *J* = 9.5 Hz, H-5), 2.82–2.72 (m, 4 H, 4 H-5), 0.89 (s, 9 H, SiC(CH_3_)_3_), 0.06 (s, 6 H, 2 SiCH_3_). ^13^C NMR (150.9 MHz, CDCl_3_): δ 168.0, 167.9, 167.5 (CO), 139.0, 138.8, 138.6, 138.5, 138.4, 138.3, 138.2, 133.7, 133.5, 133.4, 131.9, 131.8, 131.6, 131.5, 128.2, 128.0, 127.9, 127.8, 127.7, 127.6, 127.3, 127.2, 127.1, 127.0, 126.8, 126.7, 126.6, 123.3, 123.0 (Ar), 98.1 (C-1^A^), 96.7 (3 C), 96.6, 96.5 (2 C) (6 C-1), 81.2 (C-3^G^), 77.0, 76.85 (2 C), 76.8 (2 C), 76.6 (6 C-3), 76.3 (C-5^G^), 75.45, 75.4, 75.3, 75.25, 75.2, 75.15, 75.1 (6 C-4, Ph*C*H_2_), 74.6 (2 C), 74.4 (5 C), 74.3 (2 C), 74.1 (2 C) (6 C-5, 5 Ph*C*H_2_), 73.0 (Ph*C*H_2_), 72.5, 72.4, 72.3 72.1 (5 C) (C-4^G^, 7 Ph*C*H_2_), 68.1 (2 C, 2 C-6), 67.8 (O*C*H_2_CH_2_N_3_), 67.3 (C-6), 67.1 (4 C, 4 C-6), 56.8 (C-2), 56.6 (5 C, 5 C-2), 55.4 (C-2), 50.3 (OCH_2_*C*H_2_N_3_), 25.9 (SiC(*C*H_3_)_3_), −3.8, −4.6 (2 SiCH_3_). HRMS (ESI) *m/z* found: 1,767.6854 [M + 2NH_4_]^2+^; calcd. for C_204_H_194_N_10_O_43_Si: 1,767.6874.

##### 2-Azidoethyl3, 6-di-O-benzyl-2-deoxy-2-phthalimido-β-d-glucopyranosyl-(1→4)-3,6-di-O-benzyl-2-deoxy-2-phthalimido-β-d-glucopyranosyl-(1→4)-3,6-di-O-benzyl-2-deoxy-2-phthalimido-β-d-glucopyranosyl-(1→4)-3,6-di-O-benzyl-2-deoxy-2-phthalimido-β-d-glucopyranosyl-(1→4)-3,6-di-O-benzyl-2-deoxy-2-phthalimido-β-d-glucopyranosyl-(1→4)-3,6-di-O-benzyl-2-deoxy-2-phthalimido-β-d-glucopyranosyl-(1→4)-3,6-di-O-benzyl-2-deoxy-2-phthalimido-β-d-glucopyranoside (**14**)

Aqueous 40% hydrofluoric acid (0.6 ml) was added to a solution of silyl ether **13** (296 mg, 84.5 μmol) in acetonitrile (6 ml). Dichloromethane was added until the turbid solution became clear (~1 ml). The mixture was stirred for 78 h, followed by the method described above for trisaccharide **10**. Purification by column chromatography (toluene/ethyl acetate 85:15→3:1) produced 4-OH heptasaccharide **14** (265 mg, 93%) as a colorless foam, [α]_D_ +11 (*c* 1, chloroform). ^1^H NMR (600 MHz, CDCl_3_): δ 7.90–6.60 (m, 98 H, Ar), 5.24 (d, 1 H, *J*_1,2_ = 8.5 Hz, H-1^G^), 5.04 (d, 1 H, *J*_1,2_ = 7.6 Hz, H-1), 5.01–4.96 (m, 4 H, 4 H-1), 4.94–4.92 (m, 1 H, H-1^A^), 4.81–4.66 (m, 8 H, 8 benzylic H), 4.49 (d, 1 H, *J* = 12.1 Hz, benzylic H), 4.47 (d, 1 H, *J* = 11.4 Hz, benzylic H), 4.43–3.95 (m, 40 H, 7 H-2, 7 H-3, 6 H-4, 20 benzylic H), 3.78–3.72 (m, 2 H, H-4^G^, OC*Ha*CHbCH_2_N_3_), 3.63 (dd, 1 H, *J*_6a,5_ = 4.0 Hz, *J*_6a,6b_ = 9.7 Hz, H-6a^G^), 3.45 (dd, 1 H, *J*_6b,5_ = 6.3 Hz, H-6b^G^), 3.42–3.17 (m, 11 H, 2 H-5, 7 H-6, OCHaC*Hb*CH_2_N_3_, OCH_2_C*Ha*HbN_3_), 3.10 (br. d, 1 H, *J*_6a,6b_ = 10.8 Hz, H-6), 3.07 (br. s, 1 H, OH), 3.02–2.94 (m, 5 H, 4 H-6, OCH_2_CHa*Hb*N_3_), 2.81 (br. d, 1 H, *J* = 9.7 Hz, H-5), 2.79–2.69 (m, 4 H, 4 H-5). ^13^C NMR (150.9 MHz, CDCl_3_): δ 168.1, 167.9, 167.5 (CO), 138.8, 138.5, 138.4, 138.3, 138.2, 137.5, 133.8, 133.7, 133.5, 133.4, 131.9, 131.6, 128.4, 128.2, 128.0, 127.9, 127.8, 127.7, 127.6, 127.3, 127.2, 127.1, 127.0, 126.8, 126.7, 126.6, 123.3, 123.0 (Ar), 98.1 (C-1^A^), 96.8, 96.7, 96.5 (4C) (6 C-1), 78.3 (C-3^G^), 76.9, 76.8 (4 C), 76.6 (6 C-3), 75.6 (C-4^G^), 75.4 (2 C), 75.2 (2 C), 75.1 (2 C) (6 C-4), 74.6, 74.5, 74.4 (3 C), 74.3 (4 C), 74.1 (3 C) (6 C-5, 6 Ph*C*H_2_), 73.6 (Ph*C*H_2_), 72.6 (2 C), 72.5, 72.3, 72.1 (4 C) (C-5^G^, 7 Ph*C*H_2_), 71.1 (C-6^G^), 68.1, 67.8, 67.2, 67.1 (4 C) (6 C-6, O*C*H_2_CH_2_N_3_), 56.6 (5 C), 56.1, 55.4 (7 C-2), 50.3 (OCH_2_*C*H_2_N_3_). HRMS (ESI) *m/z* found: 1,710.6410 [M + 2NH_4_]^2+^; calcd. for C_198_H_180_N_10_O_43_: 1,710.6441.

##### 2-Azidoethyl3, 6-di-O-benzyl-2-deoxy-2-trifluoroacetamido-β-d-glucopyranosyl-(1→4)-3,6-di-O-benzyl-2-deoxy-2-trifluoroacetamido-β-d-glucopyranosyl-(1→4)-3,6-di-O-benzyl-2-deoxy-2-trifluoroacetamido-β-d-glucopyranoside (**15**)

Hydrazine hydrate (0.3 ml) was added to a suspension of trisaccharide **10** (78 mg, 52 μmol) in ethanol (3 ml), and the mixture was heated at 80°C with stirring for 4 h. The solvent was evaporated, and the residue was dried under vacuum for 1 h to remove traces of hydrazine hydrate. The product was dissolved in dichloromethane (4 ml), insoluble phthalohydrazide was filtered off and thoroughly washed with dichloromethane, and the filtrate was concentrated. The residue was dissolved in dry dichloromethane (3 ml), and then triethylamine (87 μl, 0.63 mmol) and pentafluorophenyl trifluoroacetate (55 μl, 0.31 mmol) were added. Methanol (50 μl) was added after 2 h, followed by dilution with chloroform after 30 min. The mixture was then washed successively with aqueous 1 M hydrochloric acid, water, and saturated aqueous sodium bicarbonate and then concentrated. The residue was purified by column chromatography (toluene/ethyl acetate 4:1) to produce N-trifluoroacetylated trisaccharide **15** (60 mg, 82%) as a colorless foam, [α]_D_ −36 (*c* 1, chloroform). ^1^H NMR (600 MHz, CDCl_3_): δ 7.41–7.22 (m, 30 H, Ar), 6.72 (d, 1 H, *J*_NH,2_ = 8.5 Hz, NH^A^), 5.99 (d, 1 H, *J*_NH,2_ = 8.9 Hz, NH^B^), 5.77 (d, 1 H, *J*_NH,2_ = 9.1 Hz, NH^C^), 4.85 (d, 1 H, *J* = 11.8 Hz, benzylic H), 4.80 (d, 1 H, *J* = 11.8 Hz, benzylic H), 4.78 (d, 1 H, *J* = 12.1 Hz, benzylic H), 4.69 (d, 1 H, *J* = 12.1 Hz, benzylic H), 4.67 (d, 1 H, *J* = 12.1 Hz, benzylic H), 4.65 (d, 1 H, *J*_1,2_ = 7.2 Hz, H-1^A^), 4.60 (d, 1 H, *J* = 11.8 Hz, benzylic H), 4.54 (d, 1 H, *J* = 11.8 Hz, benzylic H), 4.51 (d, 1 H, *J* = 12.1 Hz, benzylic H), 4.45 (s, 2 H, 2 benzylic H), 4.39 (d, 1 H, *J* = 12.1 Hz, benzylic H), 4.29 (d, 1 H, *J*_1,2_ = 8.5 Hz, H-1^B^), 4.22 (d, 1 H, *J* = 12.1 Hz, benzylic H), 4.21 (d, 1 H, *J*_1,2_ = 8.0 Hz, H-1^C^), 4.00–3.96 (m, 1 H, OC*Ha*HbCH_2_N_3_), 3.95 (t, 1 H, *J* = 7.4 Hz, H-4^A^), 3.90 (t, 1 H, *J* = 8.5 Hz, H-4^B^), 3.83–3.77 (m, 3 H, H-2^A^, H-2^B^, H-2^C^), 3.74 (t, 1 H, *J* = 7.8 Hz, H-3^A^), 3.71 (t, 1 H, *J* = 8.9 Hz, H-4^C^), 3.68–3.66 (m, 2 H, H-6a^A^, H-6a^B^), 3.64–3.58 (m, 2 H, H-6a^C^, OCHa*Hb*CH_2_N_3_), 3.49–3.40 (m, 5 H, H-5^A^, H-6b^A^, H-6b^B^, H-6b^C^, OCH_2_C*Ha*HbN_3_), 3.31–3.26 (m, 1 H, OCH_2_CHa*Hb*N_3_), 3.26–3.22 (m, 1 H, H-5^C^), 3.21 (br. s, 1 H, OH), 3.17–3.09 (m, 3 H, H-3^B^, H-3^C^, H-5^B^). ^13^C NMR (150.9 MHz, CDCl_3_): δ 157.2 (cluster of q, ^2^*J*_C,F_ = 37.6 Hz, CF_3_*C*O), 138.1, 138.0, 137.4, 137.1, 129.0, 128.8, 128.7, 128.6, 128.5, 128.4, 128.3, 128.0, 127.9, 127.7, 127.6 (Ar), 115.6 (cluster of q, ^1^*J*_C,F_ = 289 Hz, *C*F_3_CO), 99.7 (C-1^A^), 99.5 (C-1^B^), 99.2 (C-1^C^), 79.8 (C-3^C^), 78.2 (C-3^B^), 77.6 (C-3^A^), 76.4 (C-4^B^), 76.0 (C-4^A^), 74.7 (2 C, C-4^C^, C-5^A^), 74.1 (2 C, C-5^B^, Ph*C*H_2_), 73.9, 73.85, 73.8 (3 Ph*C*H_2_), 73.6 (2 C, 2 Ph*C*H_2_), 72.7 (C-5^C^), 71.0 (C-6^C^), 68.3 (2 C, C-6^A^, C-6^B^), 68.2 (O*C*H_2_CH_2_N_3_), 55.3 (2 C, 2 C-2), 55.1 (C-2), 50.5 (OCH_2_*C*H_2_N_3_). HRMS (ESI) *m/z* found: 1,421.4656 [M + Na]^+^; calcd. for C_68_H_71_F_9_N_6_O_16_: 1,421.4675.

##### 2-Azidoethyl3, 6-di-O-benzyl-2-deoxy-2-trifluoroacetamido-β-d-glucopyranosyl-(1→4)-3,6-di-O-benzyl-2-deoxy-2-trifluoroacetamido-β-d-glucopyranosyl-(1→4)-3,6-di-O-benzyl-2-deoxy-2-trifluoroacetamido-β-d-glucopyranosyl-(1→4)-3,6-di-O-benzyl-2-deoxy-2-trifluoroacetamido-β-d-glucopyranosyl-(1→4)-3,6-di-O-benzyl-2-deoxy-2-trifluoroacetamido-β-d-glucopyranoside (**16**)

Pentasaccharide **12** (87 mg, 35.6 μmol) was treated with hydrazine hydrate (0.3 ml) in ethanol (3 ml) at 80°C for 5 h, followed by the procedure described above for trisaccharide **15**.The product was N-trifluoroacetylated with pentafluorophenyl trifluoroacetate (62 ml, 0.36 mmol) in the presence of triethylamine (100 μl, 0.71 mmol) in dichloromethane (3 ml). Purification by column chromatography (toluene/ethyl acetate 85:15→4:1) produced pentasaccharide **16** (65 mg, 80%) as a colorless foam, [α]_D_ −52 (*c* 1, chloroform). ^1^H NMR (600 MHz, CDCl_3_): δ 7.38–7.17 (m, 50 H, Ar), 6.64 (d, 1 H, *J*_NH,2_ = 7.8 Hz, NH), 5.77 (d, 1 H, *J*_NH,2_ = 8.9 Hz, NH), 5.65–5.56 (m, 3 H, 3 NH), 4.88–4.74 (m, 5 H, 5 benzylic H), 4.67–4.60 (m, 3 H, H-1^A^, 2 benzylic H), 4.57 (d, 1 H, *J* = 11.8 Hz, benzylic H), 4.53 (d, 1 H, *J* = 12.3 Hz, benzylic H), 4.51–4.37 (m, 7 H, 7 benzylic H), 4.33 (d, 1 H, *J* = 11.8 Hz, benzylic H), 4.23 (d, 1 H, *J*_1,2_ = 8.5 Hz, H-1), 4.16–4.11 (m, 3 H, 3 benzylic H), 4.09 (d, 1 H, *J*_1,2_ = 8.5 Hz, H-1), 4.06 (d, 1 H, *J*_1,2_ = 8.2 Hz, H-1), 4.02 (d, 1 H, *J*_1,2_ = 8.5 Hz, H-1), 3.97–3.93 (m, 1 H, OC*Ha*HbCH_2_N_3_), 3.90 (t, 1 H, *J* = 7.2 Hz, H-4^A^), 3.87–3.70 (m, 9 H, 5 H-2, H-3^A^, 3 H-4), 3.67 (t, 1 H, *J* = 8.9 Hz, H-3^E^), 3.65–3.54 (m, 4 H, 3 H-6, OCHa*Hb*CH_2_N_3_), 3.46–3.37 (m, 9 H, H-5^A^, 7 H-6, OCH_2_C*Ha*HbN_3_), 3.27–3.23 (m, 1 H, OCH_2_CHa*Hb*N_3_), 3.23–3.18 (m, 1 H, H-5^E^), 3.17 (br. s, 1 H, OH), 3.09–2.96 (m, 5 H, 2 H-3, 3 H-5), 2.90–2.84 (m, 2 H, 2 H-3). ^13^C NMR (150.9 MHz, CDCl_3_): δ 157.1 (cluster of q, ^2^*J*_C,F_ = 37.6 Hz, CF_3_*C*O), 138.4, 138.2, 138.1, 138.0, 137.3, 137.1, 136.9, 129.2, 129.1, 128.9, 128.8, 128.7, 128.6, 128.5, 128.4, 128.3, 128.0, 127.9, 127.8, 127.7, 127.6 (Ar), 115.6 (cluster of q, ^1^*J*_C,F_ = 289 Hz, *C*F_3_CO), 99.7, 99.5, 99.4, 99.3, 99.2 (5 C-1), 79.9 (C-3^E^), 78.6, 78.5, 78.0 (3 C-3), 77.5 (C-3^A^), 76.8, 76.7, 76.6 (3 C-4), 75.9 (C-4^A^), 74.8 (C-4^E^), 74.6 (C-5^A^), 74.2, 74.1, 74.0 (2 C), 73.9, 73.8 (3 C), 73.6 (5 C) (10 Ph*C*H_2_, 3 C-5), 72.6 (C-5^E^), 71.2 (C-6^E^), 68.3, 68.2, 68.1, 68.0 (2 C) (4 C-6, O*C*H_2_CH_2_N_3_), 55.2 (2 C), 55.1 (2 C), 55.0 (5 C-2), 50.5 (OCH_2_*C*H_2_N_3_). HRMS (ESI) *m/z* found: 2,290.7993 [M + NH_4_]^+^; calcd. for C_112_H_115_F_15_N_8_O_26_: 2,290.8021.

##### 2-Azidoethyl3, 6-di-O-benzyl-2-deoxy-2-trifluoroacetamido-β-d-glucopyranosyl-(1→4)-3,6-di-O-benzyl-2-deoxy-2-trifluoroacetamido-β-d-glucopyranosyl-(1→4)-3,6-di-O-benzyl-2-deoxy-2-trifluoroacetamido-β-d-glucopyranosyl-(1→4)-3,6-di-O-benzyl-2-deoxy-2-trifluoroacetamido-β-d-glucopyranosyl-(1→4)-3,6-di-O-benzyl-2-deoxy-2-trifluoroacetamido-β-d-glucopyranosyl-(1→4)-3,6-di-O-benzyl-2-deoxy-2-trifluoroacetamido-β-d-glucopyranosyl-(1→4)-3,6-di-O-benzyl-2-deoxy-2-trifluoroacetamido-β-d-glucopyranoside (**17**)

Heptasaccharide **14** (85 mg, 25.1 μmol) was treated with hydrazine hydrate (0.3 ml) in ethanol (3 ml) at 85°C for 5 h, followed by the method described above for trisaccharide **15**. The product was N-trifluoroacetylated with pentafluorophenyl trifluoroacetate (60 μl, 0.35 mmol) in the presence of triethylamine (98 μl, 0.70 mmol) in dichloromethane (3 ml). Purification by column chromatography (toluene/ethyl acetate 85:15→4:1) produced heptasaccharide **17** (61 mg, 77%) as a colorless foam, [α]_D_ −71 (*c* 1, chloroform). ^1^H NMR (600 MHz, CDCl_3_): δ 7.38–7.14 (m, 70 H, Ar), 6.60 (d, 1 H, *J*_NH,2_ = 7.8 Hz, NH), 5.77 (d, 1 H, *J*_NH,2_ = 9.1 Hz, NH), 5.64 (d, 1 H, *J*_NH,2_ = 9.5 Hz, NH), 5.63–5.57 (m, 4 H, 4 NH), 4.78–4.74 (m, 7 H, 7 benzylic H), 4.65–4.61 (m, 3 H, H-1^A^, 2 benzylic H), 4.58 (d, 1 H, *J* = 11.8 Hz, benzylic H), 4.53 (d, 1 H, *J* = 12.1 Hz, benzylic H), 4.51–4.40 (m, 12 H, 12 benzylic H), 4.34 (d, 1 H, *J* = 12.1 Hz, benzylic H), 4.26 (d, 1 H, *J*_1,2_ = 8.7 Hz, H-1), 4.17–4.04 (m, 9 H, 5 H-1, 4 benzylic H), 3.97–3.92 (m, 1 H, OC*Ha*HbCH_2_N_3_), 3.91 (t, 1 H, *J* = 7.0 Hz, H-4^A^), 3.88–3.71 (m, 13 H, 7 H-2, 5 H-4, H-3^A^), 3.68 (t, 1 H, *J* = 8.9 Hz, H-3^G^), 3.65–3.55 (m, 4 H, 3 H-6, OCHa*Hb*CH_2_N_3_), 3.46–3.37 (m, 13 H, H-5^A^, 11 H-6, OCH_2_C*Ha*HbN_3_), 3.28–3.24 (m, 1 H, OCH_2_CHa*Hb*N_3_), 3.23–3.18 (m, 1 H, H-5^G^), 3.13–2.97 (m, 8 H, 2 H-3, 5 H-5, OH), 2.94–2.87 (m, 4 H, 4 H-3). ^13^C NMR (150.9 MHz, CDCl_3_): δ 157.1 (cluster of q, ^2^*J*_C,F_ = 37.6 Hz, CF_3_*C*O), 138.4, 138.3, 138.2, 138.1, 138.0, 137.5, 137.2, 137.1, 137.0, 129.1, 129.0, 128.9, 128.8, 128.7, 128.6, 128.5, 128.4, 128.3, 128.0, 127.9, 127.8, 127.7, 127.6 (Ar), 115.7 (cluster of q, ^1^*J*_C,F_ = 289 Hz, *C*F_3_CO), 99.7, 99.6, 99.5 (4 C), 99.3 (7 C-1), 80.0 (C-3^G^), 78.7 (3 C), 78.6, 78.1 (5 C-3), 77.5 (C-3^A^), 76.9 (2 C), 76.8, 76.7, 76.5 (5 C-3), 75.9 (C-4^A^), 74.8 (2 C, C-4^G^, C-5^A^), 74.2 (5 C), 74.0 (5 C), 73.9, 73.8, 73.7 (7 C) (14 Ph*C*H_2_, 5 C-5), 72.8 (C-5^G^), 71.2 (C-6^G^), 68.5, 68.4, 68.3 (2 C), 68.2 (3 C) (6 C-6, O*C*H_2_CH_2_N_3_), 55.4, 55.3, 55.2 (4 C), 55.0 (7 C-2), 50.5 (OCH_2_*C*H_2_N_3_). HRMS (ESI) *m/z* found: 1,591.5591 [M + 2NH_4_]^2+^; calcd. for C_156_H_159_F_21_N_10_O_36_: 1,591.5630.

##### 2-Aminoethyl2-deoxy-2-trifluoroacetamido-β-d-glucopyranosyl-(1→4)-2-deoxy-2-trifluoroacetamido-β-d-glucopyranosyl-(1→4)-2-deoxy-2-trifluoroacetamido-β-d-glucopyranoside (**18**)

Palladium hydroxide on carbon (Pd(OH)_2_/C) (65 mg) was added to a solution of benzylated trisaccharide **15** (57 mg, 40.7 μmol) in a mixture of methanol/water/acetic acid (3:1:0.5; 3 ml), and the mixture was vigorously stirred under hydrogen for 4 h. The catalyst was filtered off through Celite and thoroughly washed with aqueous 60% methanol, and the filtrate was concentrated. The residue was subjected to gel chromatography on column B. The obtained product was further purified on a reversed-phase C-18 column (10 × 40 mm) and washed with a gradient of methanol in water (0→50%). The appropriate fractions were pooled, concentrated, and freeze-dried to give pure trisaccharide **18** (27 mg, 79%) as an amorphous glassy solid, [α]_D_ −22 (*c* 1, water). ^1^H NMR (600 MHz, D_2_O): δ 4.72 (d, 1 H, *J*_1,2_ = 8.1 Hz, H-1), 4.71 (d, 1 H, *J*_1,2_ = 7.7 Hz, H-1), 4.66 (d, 1 H, *J*_1,2_ = 8.4 Hz, H-1), 4.07–4.03 (m, 1 H, OC*Ha*HbCH_2_NH_2_), 3.94 (dd, 1 H, *J*_6a,5_ = 1.8 Hz, *J*_6a,6b_ = 12.3 Hz, H-6a^C^), 3.92–3.80 (m, 8 H, H-2^A,B,C^, H-3^A,B^, H-6a^A,B^, OCHa*Hb*CH_2_NH_2_), 3.76 (dd, 1 H, *J*_6b,5_ = 5.5 Hz, H-6b^C^), 3.74–3.64 (m, 5 H, H-3^C^, H-4^A,B^, H-6b^A,B^), 3.60–3.48 (m, 4 H, H-4^C^, H-5^A, B, C^), 3.24–3.14 (m, 2 H, OCH_2_C*H*_2_N). ^13^C NMR (150.9 MHz, D_2_O): δ 101.6, 101.4 (2 C) (C-1^A, B, C^), 79.9, 79.6 (C-4^A,B^), 77.3 (C-5^C^), 75.9, 75.8 (C-5^A,B^), 74.1 (C-3^C^), 72.9, 72.7 (C-3^A,B^), 71.0 (C-4^C^), 67.5 (O*C*H_2_CH_2_NH_2_), 61.8 (C-6^C^), 61.2 (2 C) (C-6^A,B^), 57.5, 57.1, 56.8 (C-2^A, B, C^), 40.7 (OCH_2_*C*H_2_NH_2_). HRMS (ESI) *m/z* found: 833.2143 [M + H]^+^; calcd. for C_26_H_37_F_9_N_4_O_16_: 833.2134.

##### 2-Aminoethyl2-deoxy-2-trifluoroacetamido-β-d-glucopyranosyl-(1→4)-2-deoxy-2-trifluoroacetamido-β-d-glucopyranosyl-(1→4)-2-deoxy-2-trifluoroacetamido-β-d-glucopyranosyl-(1→4)-2-deoxy-2-trifluoroacetamido-β-d-glucopyranosyl-(1→4)-2-deoxy-2-trifluoroacetamido-β-d-glucopyranoside (**19**)

Acetic acid (0.4 ml), water (0.8 ml), and Pd(OH)_2_/C (70 mg) were added to a solution of protected pentasaccharide **16** (65 mg, 28.6 μmol) in methanol (2.4 ml). The resulting mixture was stirred under hydrogen for 4 h, followed by the method described above for trisaccharide **18**. Pure pentasaccharide **19** (25 mg, 65%) was obtained as a fluffy amorphous solid, [α]_D_ −20 (*c* 1, water). ^1^H NMR (600 MHz, D_2_O): δ 4.72–4.68 (m, 3 H, 3 H-1), 4.66 (d, 2 H, *J*_1,2_ = 8.4 Hz, 2 H-1), 4.07–4.03 (m, 1 H, OC*Ha*HbCH_2_NH_2_), 3.94 (dd, 1 H, *J*_6a,5_ = 2.0 Hz, *J*_6a,6b_ = 12.3 Hz, H-6a^E^), 3.92–3.81 (m, 14 H, 5 H-2, 4 H-3, 4 H-6a, OCHa*Hb*CH_2_NH_2_), 3.76 (dd, 1 H, *J*_6b,5_ = 5.5 Hz, H-6b^E^), 3.74–3.63 (m, 9 H, H-3^E^, 4 H-4, 4 H-6b), 3.60–3.48 (m, 6 H, H-4^E^, 5 H-5), 3.26–3.16 (m, 2 H, OCH_2_C*H*_2_N). ^13^C NMR (150.9 MHz, D_2_O): δ 160.8 (cluster of q, ^2^*J*_C,F_ = 37.6 Hz, CF_3_*C*O), 117.0 (cluster of q, ^1^*J*_C,F_ = 287 Hz, *C*F_3_CO), 101.7, 101.3 (4 C) (5 C-1), 79.8, 79.3 (3 C) (4 C-4), 77.3 (C-5^E^), 75.8 (3 C), 75.7 (4 C-5), 74.0 (C-3^E^), 72.8, 72.7, 72.6 (2 C) (4 C-3), 70.9 (C-4^E^), 67.2 (O*C*H_2_CH_2_NH_2_), 61.8 (C-6^E^), 61.2, 61.1 (3 C) (4 C-6), 57.5, 57.0 (3 C), 56.7 (5 C-2), 40.6 (OCH_2_*C*H_2_NH_2_). HRMS (ESI) *m/z* found: 1,347.3146 [M + H]^+^; calcd. for C_24_H_57_F_15_N_6_O_26_: 1,347.3156.

##### 2-Aminoethyl2-deoxy-2-trifluoroacetamido-β-d-glucopyranosyl-(1→4)-2-deoxy-2-trifluoroacetamido-β-d-glucopyranosyl-(1→4)-2-deoxy-2-trifluoroacetamido-β-d-glucopyranosyl-(1→4)-2-deoxy-2-trifluoroacetamido-β-d-glucopyranosyl-(1→4)-2-deoxy-2-trifluoroacetamido-β-d-glucopyranosyl-(1→4)-2-deoxy-2-trifluoroacetamido-β-d-glucopyranosyl-(1→4)-2-deoxy-2-trifluoroacetamido-β-d-glucopyranoside (**20**)

Acetic acid (0.4 ml), water (0.8 ml), and Pd(OH)_2_/C (63 mg) were added to a solution of protected heptasaccharide **17** (61 mg, 19.4 μmol) in methanol (3 ml), and the mixture was vigorously stirred under hydrogen for 5 h, followed by the same method described above for trisaccharide **18**. Pure heptasaccharide **20** (30 mg, 83%) was obtained as a fluffy amorphous solid, [α]_D_ −17 (*c* 1, water). ^1^H NMR (600 MHz, D_2_O): δ 4.73–4.68 (m, 6 H, 6 H-1), 4.65 (d, 1 H, *J*_1,2_ = 7.9 Hz, H-1), 4.05–4.01 (m, 1 H, OC*Ha*HbCH_2_NH_2_), 3.92 (br. d, 1 H, *J*_6a,6b_ = 12.3 Hz, H-6a^G^), 3.91–3.79 (m, 20 H, 7 H-2, 6 H-3, 6 H-6a, OCHa*Hb*CH_2_NH_2_), 3.74 (dd, 1 H, *J*_6b,5_ = 5.6 Hz, H-6b^G^), 3.71–3.60 (m, 13 H, H-3^G^, 6 H-4, 6 H-6b), 3.58–3.47 (m, 8 H, H-4^G^, 7 H-5), 3.26–3.14 (m, 2 H, OCH_2_C*H*_2_N), 1.90 (s, 3 H, CH_3_COO^−^). ^13^C NMR (150.9 MHz, D_2_O): δ 160.7 (cluster of q, ^2^*J*_C,F_ = 37.6 Hz, CF_3_*C*O), 117.0 (cluster of q, ^1^*J*_C,F_ = 287 Hz, *C*F_3_CO), 101.6, 101.4 (6 C) (7 C-1), 80.1, 79.9 (5 C) (6 C-4), 77.4 (C-5^G^), 75.9 (6 C, 6 C-5), 74.2 (C-3^G^), 72.9, 72.8, 72.7 (4 C) (6 C-3), 71.2 (C-4^G^), 67.3 (O*C*H_2_CH_2_NH_2_), 61.9 (C-6^G^), 61.3 (6 C, 6 C-6), 57.6, 57.2 (5 C), 56.9 (7 C-2), 40.8 (OCH_2_*C*H_2_NH_2_), 24.6 (*C*H_3_OO^−^). HRMS (ESI) *m/z* found: 1,861.4188 [M + H]^+^; calcd. for C_58_H_77_F_21_N_8_O_36_: 1,861.4178.

##### Biotin-Tagged Trisaccharide (**25**)

Solutions of triethylamine in dimethylformamide (DMF) (20/500 μl; 24 μl, 6.6 μmol) and activated ester **21** in DMF (64 mM; 106 μl, 6.6 μmol) were added to a solution of trisaccharide **18** (4.6 mg, 5.5 μmol) in DMF (0.4 ml), and the resulting mixture was stirred for 2 h. The solvent was removed under vacuum, and the residue was dissolved in water (1 ml). The solution was repeatedly extracted with ethyl acetate (4 × 2 ml). The organic extracts were discarded; the aqueous solution was applied on a Sep Pak C-18 cartridge eluted with a gradient of methanol in water (0→50%) to produce biotinylated product **22** (HRMS (ESI) *m/z* found: 1,416.4654 [M + Na] ^+^; calculated for C_51_H_80_F_9_N_7_O_25_S: 1,416.4673). Sodium hydroxide (1 M, 0.2 ml) was added to a solution of product **22** in aqueous 80% methanol (1 ml); the mixture was stirred for 16 h at RT, neutralized with acetic acid, and then concentrated. Gel chromatography of the residue on column C produced title product **25** (4.0 mg, 66%) as a fluffy amorphous solid. ^1^H NMR (600 MHz, D_2_O; selected signals) oligosaccharide moiety: δ 4.78 (d, 1 H, *J*_1,2_ = 8.4 Hz, H-1), 4.76 (d, 1 H, *J*_1,2_ = 8.4 Hz, H-1), 4.74 (d, 1 H, *J*_1,2_ = 8.4 Hz, H-1), 3.05 (dd, 3 H, *J*_2,1_ = 8.4 Hz, *J*_2,3_ = 10.0 Hz, 3 H-2), 1.93 (s, 3 H, CH_3_COO^−^); biotin moiety: δ 4.62 (dd, 1 H, *J*_6a,3a_ = 7.9 Hz, *J*_6a,6_ = 4.8 Hz, H-6a), 4.44 (dd, 1 H, *J*_3a,6a_ = 7.9 Hz, *J*_3a,4_ = 4.6 Hz, H-3a), 3.35 (m, 1 H, H-4), 3.01 (dd, 1 H, *J*_6cis,6*trans*_ = 13.0 Hz, *J*_6cis,6*a*_ = 5.1 Hz, H-6*cis*), 2.80 (d, 1 H, *J*_6trans,6*cis*_ = 13.0 Hz, H-6*trans*), 2.29 (t, 2 H, *J* = 7.3 Hz, 2 H-2′), 1.78–1.56 (m, 4 H, 2 H-3′, 2 H-5′), 1.47–1.40 (m, 2 H, 2 H-4′); linker moiety: δ 3.40 (t, 2 H, *J* = 5.5 Hz, OCH_2_C*H*_2_N), 2.57 (t, 2 H, *J* = 6.2 Hz, OCH_2_C*H*_2_CON). ^13^C NMR (150.9 MHz, D_2_O) oligosaccharide moiety: δ 100.3 (2 C), 100.0 (3 C-1), 61.4, 61.2 (2 C) (3 C-6), 57.2, 57.1, 56.8 (3 C-2), 24.2 (*C*H_3_COO^−^); biotin moiety: δ 178.0 (C-1′), 166.4 (C-2), 63.2 (C-3a), 61.5 (C-6a), 56.4 (C-4), 40.8 (C-6), 36.6 (C-2′), 29.0, 28.8, 26.2 (C-3′, C-4′, C-5′); linker moiety: δ 175.4 (CO), 37.1 (OCH_2_*C*H_2_CON). HRMS (ESI) *m/z* found: 1,128.5187 [M + Na]^+^; calcd. for C_45_H_83_N_7_O_22_S: 1,128.5204.

##### Biotin-Tagged Pentasaccharide (**26**)

Solutions of triethylamine in DMF (20/500 μl; 16 μl, 4.4 μmol) and activated ester **21** in DMF (64 mM; 71 μl, 4.4 μmol) were added to a solution of pentasaccharide **19** (5.0 mg, 3.7 μmol) in DMF (0.5 ml), and the resulting mixture was stirred for 1 h. The solvent was evaporated under vacuum, and the residue was dissolved in water (2 ml). The solution was repeatedly extracted with ethyl acetate (4 × 2.5 ml). The organic extracts were discarded; the aqueous solution was applied on a Sep Pak C-18 cartridge eluted with a gradient of methanol in water (0→50%) to produce biotinylated product **23** (HRMS (ESI) *m/z* found 1,930.5668 [M + Na] ^+^; calculated for C_67_H_100_F_15_N_9_O_35_S: 1,930.5695). Sodium hydroxide (1 M, 0.2 ml) was added to a solution of product **23** in aqueous 70% methanol (1 ml); the mixture was stirred for 16 h, neutralized with acetic acid, and then concentrated. Gel chromatography of the residue on column C produced title product **26** (3.2 mg, 60%) as a fluffy amorphous solid. ^1^H NMR (600 MHz, D_2_O; selected signals) oligosaccharide moiety: δ 4.77–4.73 (m, 5 H, 5 H-1), 3.06–3.00 (m, 5 H, 5 H-2), 1.92 (s, 5.8 H CH_3_COO^−^); biotin moiety: δ 4.61 (dd, 1 H, *J*_6a,3a_ = 8.1 Hz, *J*_6a,6_ = 5.1 Hz, H-6a), 4.43 (dd, 1 H, *J*_3a,6a_ = 8.1 Hz, *J*_3a,4_ = 4.6 Hz, H-3a), 3.34 (m, 1 H, H-4), 3.00 (dd, 1 H, *J*_6cis,6*trans*_ = 13.0 Hz, *J*_6cis,6*a*_ = 4.8 Hz, H-6*cis*), 2.79 (d, 1 H, *J*_6trans,6*cis*_ = 13.0 Hz, H-6*trans*), 2.28 (t, 2 H, *J* = 7.3 Hz, 2 H-2′), 1.77–1.55 (m, 4 H, 2 H-3′, 2 H-5′), 1.46–1.39 (m, 2 H, 2 H-4′); linker moiety: δ 3.39 (t, 2 H, *J* = 5.3 Hz, OCH_2_C*H*_2_N), 2.56 (t, 2 H, *J* = 6.2 Hz, OCH_2_C*H*_2_CON). ^13^C NMR (150.9 MHz, D_2_O) oligosaccharide moiety: δ 100.5 (2 C), 100.4 (2 C), 100.1 (5 C-1), 61.5, 61.4, 61.3 (3 C) (5 C-6), 57.3 (3 C), 57.2, 56.9 (5 C-2), 24.3 (*C*H_3_COO^−^); biotin moiety: δ 63.3 (C-3a), 61.6 (C-6a), 56.6 (C-4), 40.9 (C-6), 36.7 (C-2′), 29.1, 28.9, 26.4 (C-3′, C-4′, C-5′); linker moiety: δ 37.2 (OCH_2_*C*H_2_CON). HRMS (ESI) *m/z* found: 1,428.6728 [M + H]^+^; calcd. for C_57_H_105_N_9_O_30_S: 1,428.6761.

##### Biotin-Tagged Heptasaccharide (**27**)

Solutions of triethylamine in DMF (20/500 μl; 15 μl, 4.0 μmol) and activated ester **21** in DMF (64 mM; 65 μl, 4.0 μmol) were added to a solution of heptasaccharide **20** (6.2 mg, 3.3 μmol) in DMF (0.6 ml), and the resulting mixture was stirred for 1 h. The solvent was evaporated under vacuum, and the residue was dissolved in water (2 ml). The solution was repeatedly extracted with ethyl acetate (4 × 2.5 ml). The organic extracts were discarded; the aqueous solution was subjected to gel chromatography on column C to give product **24** (HRMS (ESI) *m/z* found 2,444.6729 [M + Na] ^+^; calculated for C_83_H_120_F_21_N_11_O_45_S: 2,444.6717). Sodium hydroxide (1 M, 0.2 ml) was added to an aqueous solution of product **24** (0.8 ml); the mixture was stirred for 16 h, neutralized with acetic acid, and concentrated. Gel chromatography of the residue on column C produced biotinylated heptasaccharide **27** (3.4 mg, 59%) as a fluffy amorphous solid. ^1^H NMR (600 MHz, D_2_O; selected signals) oligosaccharide moiety: δ 4.71 (d, 1 H, *J*_1,2_ = 8.1 Hz, H-1), 4.70–4.65 (m, 6 H, 6 H-1), 3.02–2.92 (m, 7 H, 7 H-2), 1.93 (s, 5.4 H, CH_3_COO^−^); biotin moiety: δ 4.63 (dd, 1 H, *J*_6a,3a_ = 8.1 Hz, *J*_6a,6_ = 4.8 Hz, H-6a), 4.44 (dd, 1 H, *J*_3a,6a_ = 8.1 Hz, *J*_3a,4_ = 4.6 Hz, H-3a), 3.36 (m, 1 H, H-4), 3.02 (dd, 1 H, *J*_6cis,6*trans*_ = 13.0 Hz, *J*_6cis,6*a*_ = 5.0 Hz, H-6*cis*), 2.81 (d, 1 H, *J*_6trans,6*cis*_ = 13.0 Hz, H-6*trans*), 2.30 (t, 2 H, *J* = 7.3 Hz, 2 H-2′), 1.79–1.57 (m, 4 H, 2 H-3′, 2 H-5′), 1.48–1.41 (m, 2 H, 2 H-4′); linker moiety: δ 3.41 (t, 2 H, *J* = 5.5 Hz, OCH_2_C*H*_2_N), 2.57 (t, 2 H, *J* = 6.2 Hz, OCH_2_C*H*_2_CON). ^13^C NMR (150.9 MHz, D_2_O) oligosaccharide moiety: δ 101.8 (4 C), 101.6, 101.2, 101.1 (7 C-1), 61.9, 61.5 (6 C) (7 C-6), 57.6 (5 C), 57.5, 57.2 (7 C-2), 24.4 (*C*H_3_COO^−^); biotin moiety: δ 63.4 (C-3a), 61.6 (C-6a), 56.6 (C-4), 40.9 (C-6), 36.8 (C-2′), 29.1, 29.0, 26.4 (C-3′, C-4′, C-5′); linker moiety: δ 37.3 (OCH_2_*C*H_2_CON). HRMS (ESI) *m/z* found: 875.9106 [M + 2H]^2+^; calcd. for C_69_H_127_N_11_O_38_S: 875.9105.

### Immunobiology

#### Preparation of Chitin- and Chitosan-Derived Oligosaccharides

Stock solutions and partial dilutions of chitosan (**25**–**27**, **Scheme 3**) and chitin (**28**–**30**, **Scheme 3**) oligosaccharide formulas were prepared aseptically using pre-sterilized disposable plastic wares (Eppendorf, Hamburg, Germany) and sterile water for injection (Fresenius Kabi Italia S.r.l., Verona, Italy). Solutions were sterilized using a 0.2-μm filter (Q-Max® Frisenette, Knebel, Denmark) in a laminar flow hood. The laminar flow cabinet was pre-sterilized with 70% ethanol and UV irradiation for 30 min prior to each experiment. Stock solutions were assayed with the EndoLISA® endotoxin determination kit (Hyglos, Bernried, Germany) to ascertain endotoxin-free content.

#### Cell Maintenance, Culture, and Cell Culture Exposure

Murine macrophage cell line RAW 264.7 cells (European Collection of Authenticated Cell Cultures, Salisbury, UK) were cultured in complete Dulbecco's Modified Eagle Medium and a high-glucose medium supplemented with 10% fetal bovine serum, 2 mM l-glutamine, 10,000 U/ml penicillin, and 10 mg/ml streptomycin (Sigma-Aldrich) at 37°C in a humidified atmosphere with 5% carbon dioxide until cells reached ~80% confluence. Cell exposure experiments were performed within 24–48 h. Cell viability was assessed using Trypan blue dye exclusion assay (TC20™ automated cell counter (Bio-Rad Laboratories). The starting inoculum of 1.33 × 10^6^ cells/ml/well (92% viable cells) was cultured in a 24-well cell culture plate (Sigma-Aldrich), and cells were exposed to 10 and 100 μg/well of oligosaccharides **25**–**27** and **28**–**30** for 24 and 48 h, respectively. Cell mitogens concanavalin A (Con A, 10 μg/ml), phytohemagglutinin (PHA, 10 μg/ml), lipopolysaccharide (LPS, 1 μg/ml), and poke weed mitogen (PWM, 1 μg/ml) (all Sigma-Aldrich) were used as positive controls. Cell morphology and viability were assayed prior to cell immunophenotyping and evaluation of phagocytosis and cytotoxicity. The cell culture media were separated and frozen at −20°C until further use.

#### Determination of Cell Proliferation and Cytotoxicity

The influence of oligosaccharides **25**–**27** and **28**–**30** on cytotoxicity and proliferation of RAW 264.7 cells was evaluated using the ViaLight™ Plus bioassay kit (Lonza, Walkersville, MD, USA) according to the manufacturer's recommendations. The impact of oligosaccharide exposure on cellular adenosine triphosphate (ATP) was determined by luciferase-based luminescence quantification. Briefly, the intensity of emitted light was measured using the Cytation 5 Cell Imaging Multi-Mode Reader (BioTek Instruments, Inc., Winooski, VT, USA). Light emission was recorded continuously for 1 s, and peak values were evaluated and expressed as relative light units (RLU). Values of unexposed cells were considered the control baseline. The proliferation index was calculated as the ratio of stimulated cell proliferation, i.e., cells treated with oligosaccharides **25**–**27** and **28**–**30**, to that of unexposed cells. The proliferation index of the negative control, i.e., baseline unexposed cells, was equal to one.

#### *In vitro* Quantification of Interleukins and Growth Factors

Cytokine levels in cell culture supernatants induced by exposure to oligosaccharides were assayed using Platinum ELISA® kits: Mouse IL-12 p70 [Minimum Detectable Dose [MDD] 4 pg/ml], Mouse GM-CSF (MDD 2 pg/ml), Mouse IL-17 (MDD 1.6 pg/ml), Mouse IL-6 (MDD 6.5 pg/ml), and Mouse IL-2 (MDD 5.3 pg/ml) and Instant ELISA® kits: Mouse IFNγ (MDD 4 pg/ml), Mouse tumor necrosis factor (TNF)-α (MDD 4 pg/ml), Mouse IL-10 (MDD 5.28 pg/ml), Mouse IL-1β (MDD 3 pg/ml), and Mouse IL-4 (MDD 0.6 pg/ml), all from Thermo-Fisher Scientific (Waltham, MA, USA) according to the manufacturer's instructions.

#### Immunocytometric Evaluation of Simultaneous Phagocytosis and Oxidative Burst

RAW 264.7 macrophage phagocytosis accompanied by respiratory burst was evaluated by flow cytometry (CytoFLEX; Beckman Coulter Life Sciences, Inc., Indianapolis, IN, USA). For each sample, a fluorescence histogram of 10,000 cells was generated and analyzed. Gates were set around the macrophage population to exclude debris. Measurements of phagocytosis, i.e., ingestion of bacteria, took place under controlled conditions using fluorescein-labeled opsonized *Staphylococcus aureus* particles (SPA-FITC) (Molecular Probes, Leiden, The Netherlands). Metabolic activity was determined via oxidative burst induced by the stimulated transformation of non-fluorescent hydroxyethidine (HE) (Polysciences Inc., Warrington, PA, USA) to fluorescent ethidium, which exhibited fluorescence (excitation of 488 nm) upon DNA intercalation following SPA-FITC ingestion. Aliquots of post-exposure cells in culture media (30 μl) were incubated with HE [15.75 mg in 5 ml of dimethylformamide (Merck)] and SPA-FITC for 15 min at 37°C. The reaction was terminated with ice.

The mean percentage of phagocytic cells was represented by the percentage of cells that ingested at least one SPA-FITC particle. The mean percentage of respiratory burst was represented by the percentage of cells tagged by ethidium. The mean percentage of metabolic activity was represented by the percentage of cells that ingested at least one SPA-FITC and was tagged by ethidium.

#### Fluorescence Quenching Cytometric Assay

Extracellular fluorescein isothiocyanate (FITC) fluorescence was quenched by 0.4% Trypan blue dye (Sigma-Aldrich). Immunocytometric analysis of Trypan blue-treated RAW 264.7 cells was performed following cell incubation for 15 min in the dark at 37°C. The amount of membrane-attached FITC-conjugated SPA was expressed as the difference between whole-cell phagocytosis, i.e., cells with cell-bound and internalized SPA-FITC, and the Trypan blue-quenched cell population. Metabolic activity was expressed as the percentage of cells simultaneously undergoing phagocytosis and oxidative burst.

#### Macrophage Immunophenotyping

For immunocytometric assays, RAW 264.7 cells were stained directly with fluorescein FITC-conjugated rat anti-mouse monoclonal antibodies: F4/80, CD11b, and CD14 (Thermo Fisher Scientific). The appropriate antibody isotype-negative controls were used for setting gates. The FITC-conjugated monoclonal antibodies (5 μl) and RAW 264.7 cells (50 μl) were added to the 96-well microtiter plate and incubated for 30 min in the dark at 4°C. Then, the samples were evaluated by immunoflow cytometry (CytoFLEX, Beckman Coulter Life Sciences, Inc.).

### Chitin and Chitosan

#### Preparation

Approximately 60 ml of wet *Candida albicans* CCY 29-3-100 cells was extracted according to Ferreira et al. (2006) to obtain a pellet with a sufficient amount of insoluble β-1,3-glucans (and chitin). The obtained mass of 3.80 g was extracted with 5 mass% potassium hydroxide (80 ml) for 30 min at 90°C. The sediment was washed 3× with water. Next, 30 mass% peroxide (40 ml) and acetic acid (40 ml) were added. The suspension was stirred with heating for 20 min at 100°C and then washed 3× with ultrapure water. The obtained crude chitin was washed 3× with 5 mass% potassium hydroxide at 100°C for 30 min, with three subsequent water washing steps. Finally, the sediment was suspended in 1.0 M hydrochloric acid and stirred for 30 min at RT, followed by washing 5× with ultrapure water. After lyophilization, 0.213 g of chitin was obtained (5.6% of the glucan mass).

Chitin (0.113 g) and 50 mass% sodium hydroxide (1.50 ml) were incubated at 120°C for 6 h with occasional shaking. The suspension was cooled at 5°C overnight. The next day, the suspension was centrifuged (2,000×*g*, 10 min, Hettich® Universal 320R centrifuge), the sediment was dissolved in water, and 1 M hydrochloric acid was added. The insoluble fraction was dialyzed against ultrapure water and then lyophilized. Then, 45 mg of chitosan was obtained (40% yield).

#### FTIR

Fourier-transform infrared (FTIR) spectra were measured using the Nicolet iS50 FT-IR spectrometer (Thermo Fisher Scientific) equipped with a deuterated-triglycine sulfate detector and the Omnic 9.0 software (Thermo Fisher Scientific). For each sample, 64 scans were averaged with a spectral resolution of 4 cm^−1^ in the middle region from 4,000 to 400 cm^−1^. A diamond attenuated total reflectance sampling accessory was employed for solid-state measurements. The FTIR spectra of chitin and chitosan are shown in Supplementary Figure 1.

#### NMR

Prior to NMR spectroscopy, protons in the samples were exchanged with heavy water. ^1^H NMR spectra were acquired in deuterium oxide (99.97% D) plus acetic acid (1 μl) on a Bruker AVANCE III HD 400 MHz spectrometer (Bruker, Germany) equipped with a 5-mm broad band BB-(H-F)-D-05-Z liquid N_2_ Prodigy probe with an automatic chemical shift calibration and processed using the MestReNova 14.0.1 software. The ^1^H signal of acetic acid (1.950 ppm) was used as a reference for chemical shifts. The ^1^H NMR spectrum of chitosan is shown in Supplementary Figure 2.

#### Determination of the Degree of Acetylation

Degree of acetylation (DA) was measured as previously reported by Czechowska-Biskup et al. (2012).

### Statistical Analysis

Results were expressed as mean ± SD. Normality of data distribution was determined according to the Shapiro–Wilk test at the 0.05 level of significance. Statistical evaluation was performed via one-way ANOVA and *post-hoc* Bonferroni test. Results were considered significant when differences equaled or exceeded the 95% confidence level (*P* < 0.05). Statistical analysis was performed using the ORIGIN 2018 software (OriginLab Corporation, Northampton, MA, USA). Pearson's correlation coefficients were used to compare the strength of the relationship between immunobiological variables.

## Results

### Chemistry

**Scheme 1:**
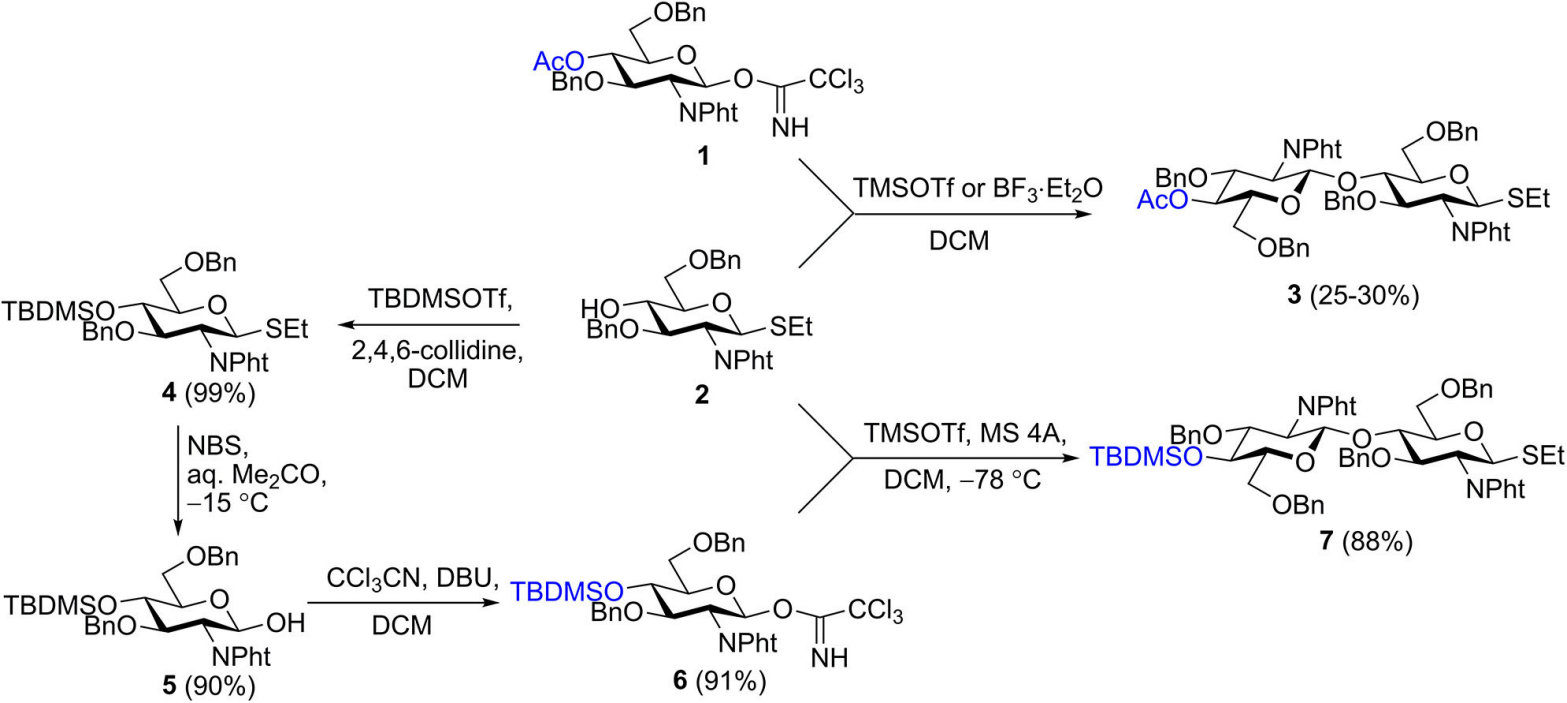
Synthesis of disaccharide donor **7**.

We recently described the synthesis of 2-aminoethyl glycosides of chito-oligomers containing 3, 5, and 7 *N*-acetyl-d-glucosamine residues (Yudina et al., 2015) and their biotinylated derivatives **28**–**30** (**Scheme 3**) (Yudina et al., 2016). Azide and phthaloyl groups were employed as a precursor of the aglycon amino group and for N-protection of glucosamine, respectively. Elongation of the oligosaccharide chain was accomplished using a disaccharide donor containing acetyl or chloroacetyl groups for temporary protection of 4-OH (Yudina et al., 2015). An attempt to prepare donor **3** directly by orthogonal glycosylation of thioglycoside **2** with imidate **1** was not overly successful (Scheme 1) due to predominant transfer of ethylthio aglycon from acceptor **2** onto donor **1** (Yudina et al., 2015). For this reason, we employed an indirect approach for the disaccharide donor, consisting of initial glycosylation of an acceptor with *p*-methoxyphenyl anomeric protection, followed by two-step transformation of *p*-methoxyphenyl glycoside into the corresponding trichloroacetimidate (Yudina et al., 2015).

The same protecting group pattern was used in the present work, but we improved considerably the preparation of the key disaccharide donor. Glycosylation of acceptor **2** with various types of donors (glycosyl bromides, sulfoxides, and trichloroacetimidates) bearing different protecting groups at O-4 (acyl, silyl) was examined. The results of this examination, together with published data (Barry et al., 2013), demonstrated that electron-withdrawing acyl protection at O-4 facilitated aglycon transfer and should thus be excluded.

The optimal result was achieved with 4-*O*-TBDMS imidate **6**, which was efficiently prepared from **2** by silylation of 4-OH (→**4**), hydrolysis of the anomeric SEt-group (Lay et al., 1998) (→ **5**), and trichloroacetimidate formation, for a total yield of 81% in three steps (Scheme 1). As 4-*O*-silylated donor **6** must possess higher reactivity than 4-*O*-acylated counterparts (Tanaka et al., 2017), one could anticipate a decrease of the proportion of aglycon transfer in its reaction with **2** (Li and Gildersleeve, 2006, 2007). Indeed, TMSOTf-promoted glycosylation of acceptor **2** with imidate **6** at low temperature provided the requisite disaccharide donor **7** in high yield and was practically unaccompanied by aglycon transfer. Thus, thioglycoside **7** was synthesized in four steps from single precursor **2** with an overall yield of 71%.

**Scheme 2:**
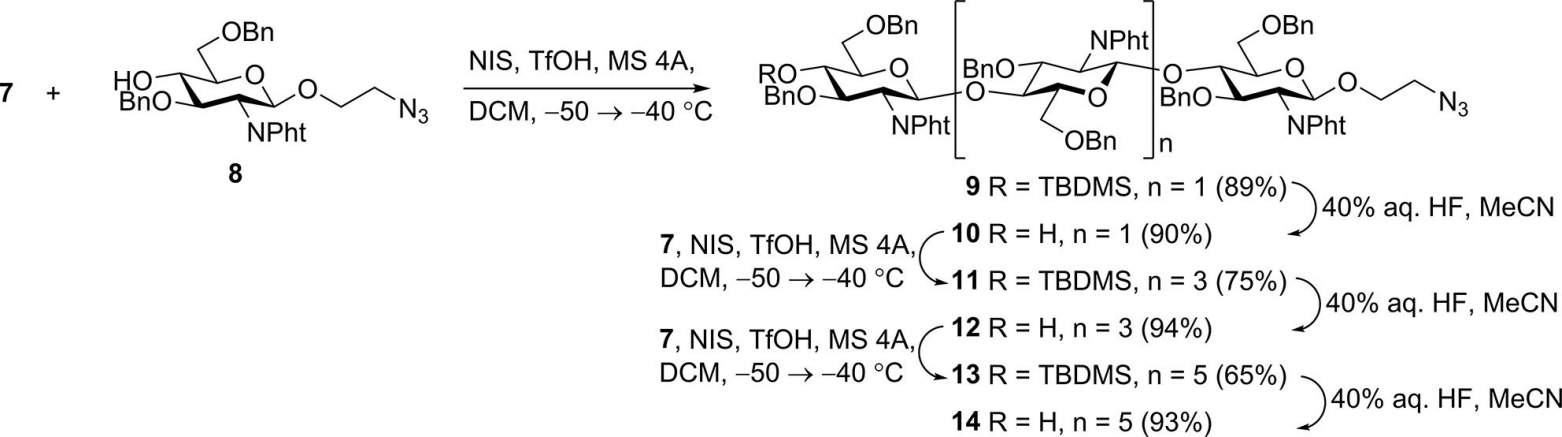
Assembly of protected chito-oligosaccharides.

N-iodosuccinimide-TfOH-promoted glycosylation of 2-azidoethyl glycoside **8** (Yudina et al., 2015) with thioglycoside **7** to smoothly produce trisaccharide **9**; from which removal of the silyl group with aqueous hydrofluoric acid in acetonitrile resulted in new glycosyl acceptor **10** (Scheme 2). Desilylation with hydrofluoric acid proceeded slowly (48–72 h) but smoothly, with minimal formation of side products. Desilylation with tetrabutylammonium fluoride in tetrahydrofuran was much faster (2–3 h), but yields of **10** were noticeably lower, apparently due to insufficient stability of *N*-phthaloyl groups under basic conditions. Glycosylation with **7** and desilylation of the glycosylation product was reiterated twice to give derivatives of chitopentaose (**11** and **12**) and chitoheptaose (**13** and **14**). Further chain elongation was not performed, as it was previously demonstrated that pentamers and hexamers possess the highest affinity for antichitosan polyclonal antibodies (Kim et al., 2000).

**Scheme 3:**
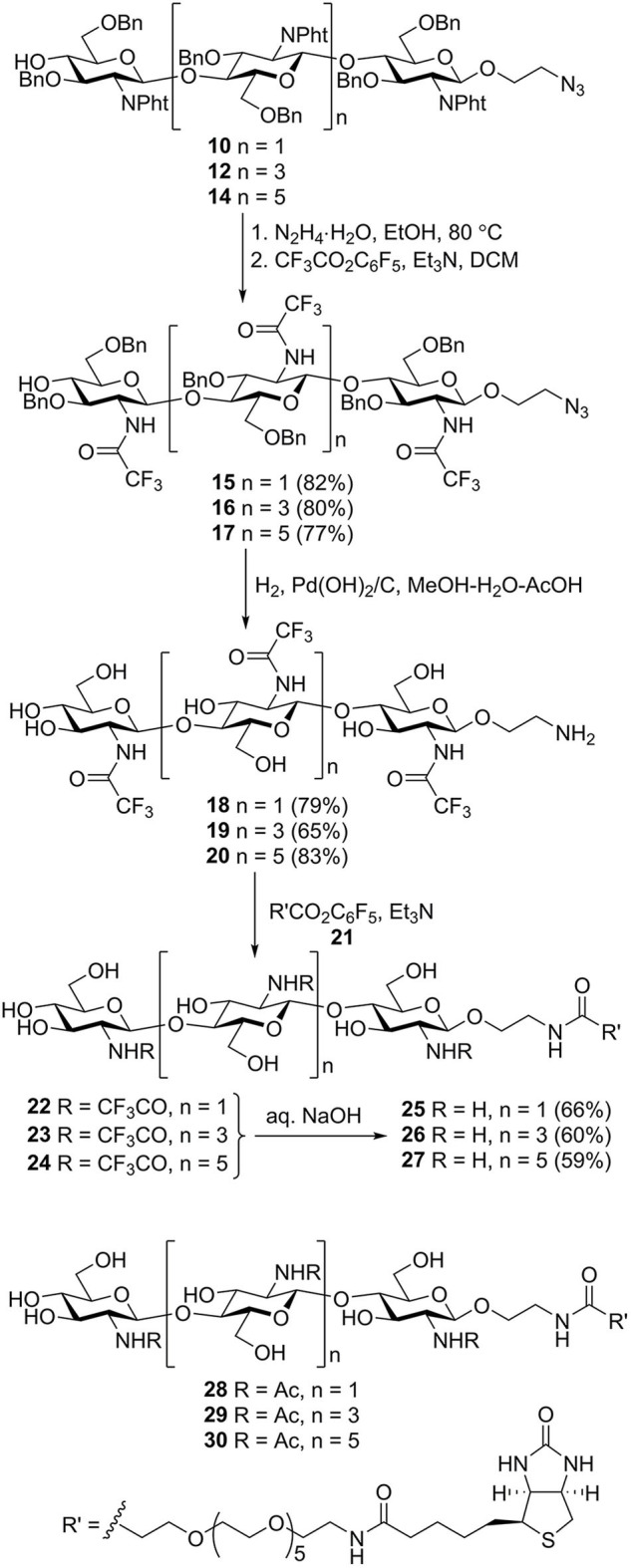
Transformation of the protected chito-oligomers in biotin-tagged chitosan oligosaccharides **25**–**27**. Structures of previously synthesized chito-oligosaccharides **28**–**30** used in this work.

*N*-phthaloyl groups in protected oligomers **10**, **12**, and **14** were removed by treatment with hydrazine hydrate, and free amino groups were protected again by trifluoroacetylation to give derivatives **15**–**17** (Scheme 3). These were subjected to simultaneous debenzylation and azide reduction by hydrogenolysis over Pd (OH)_2_/C in aqueous methanol in the presence of acetic acid (Yudina et al., 2016). ^1^H and ^13^C NMR data of obtained N-trifluoroacetylated 2-aminoethyl glycosides **18**–**20** unequivocally demonstrated that all glucosamine residues exhibited β-configuration and were connected by (1→4)-linkage. Thus, signals for H-1 in the ^1^H NMR spectra appeared as doublets with the coupling constant value *J*_1,2_ ~8 Hz. The signal location for C-1 at δ ~101.5 ppm in the ^13^C NMR spectra also confirmed β-configuration. A downfield location of signals for C-4 (δ ~80 ppm) of the internal glucosamine residues, compared with that of terminal unsubstituted glucosamine (δ ~71 ppm), confirmed the position of the glycoside bonds.

N-acylation of the spacer amino group in **18**–**20** with active ester of biotin **21** (Tsvetkov et al., 2012) containing the hydrophilic hexa (ethylene glycol) linker resulted in the formation of biotinylated products **22**–**24**. Removal of the N-trifluoroacetyl groups by mild alkaline hydrolysis produced the requisite biotin-tagged chitosan oligomers **25**–**27**. The presence of signals corresponding to oligosaccharide, biotin, and linker moieties in the NMR spectra of **25**–**27** confirmed their structure.

### Immunobiology

#### Effect of Chitin- and Chitosan-Derived Oligosaccharides on RAW 264.7 Proliferation

**Figure 1:**
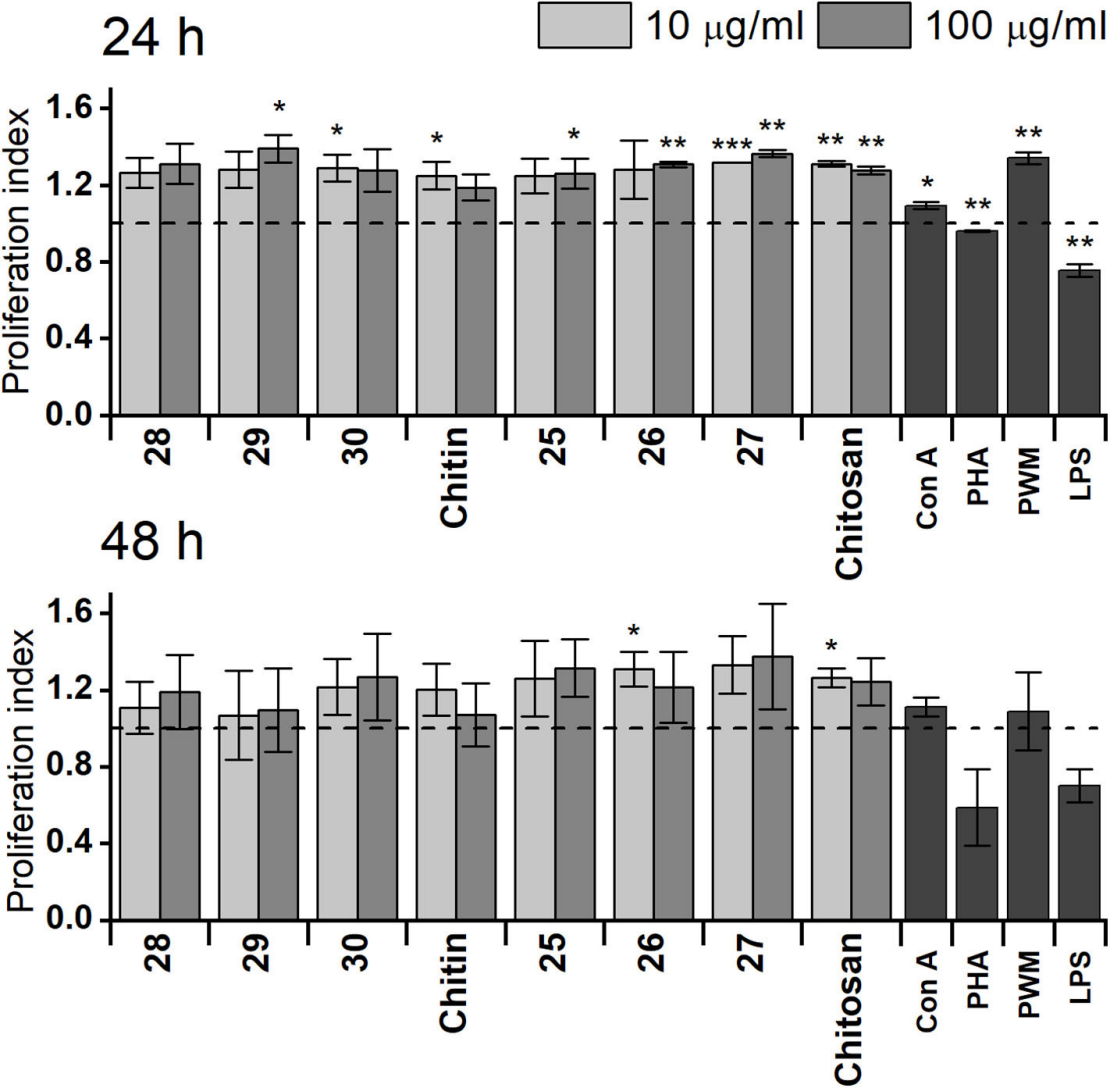
Proliferation of RAW 264.7 macrophages after exposure for 24 or 48 h with 10 or 100 μg/ml of chitin trisaccharide (**28**), pentasaccharide (**29**), heptasaccharide (**30**), and chitin, as well as chitosan trisaccharide (**25**), pentasaccharide (**26**), heptasaccharide (**27**), and chitosan. Untreated RAW 264.7 cells were used as the negative control. Concanavaline A (Con A, 10 μg/ml), phytohemagglutinin (PHA, 10 μg/ml), pokeweed mitogen (PWM, 1 μg/ml), and lipopolysaccharide (LPS, 10 μg/ml) were used as positive controls. Horizontal dashed line represents baseline. Results are expressed as the mean stimulation indexes (average relative light units in the presence of antigens/average relative light units obtained without antigen). All data are presented as mean stimulation indexes ± SD. Tests were carried out in triplicate. Statistical significance of differences between untreated and stimulated cells using one-way ANOVA and *post-hoc* Bonferroni test is expressed as: ***P < 0.001, ** 0.001 < *P* < 0.01, * 0.01 < *P* < 0.05.

The capability of synthetically prepared chitin or chitosan oligosaccharides to affect proliferation of RAW 264.7 cells was monitored by ATP bioluminescence as a cell viability marker (Figure 1) compared with that of natural chitin isolated from *C. albicans* serotype A or prepared chitosan. Obtained results after 24 h treatment revealed higher capability of chitosan oligosaccharides **25**, **26**, and **27** to increase RAW 264.7 cell proliferation than that of chitin oligosaccharides **28**, **29**, and **30**. Additionally, stimulatory activity of prepared chitosan was higher than that of natural chitin. Stimulation with longer chitosan oligosaccharides (**27**, 10 μg/ml: *P* = 2.5 × 10^8^, 100 μg/ml: *P* = 0.001) for 24 h induced significantly more pronounced RAW 264.7 cell proliferation than that with shorter chitosan oligosaccharides (**26**: 10 μg/ml: *P* = 0.12, 100 μg/ml: *P* = 0.0012, **25**: 10 μg/ml: *P* = 0.061, 100 μg/ml: *P* = 0.043), which was even greater than that of prepared chitosan (10 μg/ml: *P* = 0.0012, 100 μg/ml: *P* = 0.0026). Chitosan heptasaccharide **27** was the most effective inducer of RAW 264.7 cell proliferation (10 μg/ml: 1.32-fold and 100 μg/ml: 1.36-fold increase compared with the control). After 48 h treatment, no significant difference was observed between prepared chitin or chitosan oligosaccharides in their capability to increase proliferation of RAW 264.7 macrophages. The increase in macrophage proliferation induced by chitin or chitosan oligosaccharides was higher than (Con A, PHA, and LPS) or comparable with (PWM) the proliferation induced by mitogens.

#### Effect of Chitin- and Chitosan-Derived Oligosaccharides on RAW 264.7 Phagocytic Activity

RAW 264.7 macrophage cells exposed to chitin- and chitosan-derived oligosaccharides **28**–**30** and **25**–**27**, as well as natural *Candida* chitin and chitosan, were subjected to bacterial phagocytosis and respiratory burst analysis (Tables 1, 2). In parallel, phagocytosis of cells exposed to cell mitogens was used as positive control (Table 3). The influence of applied concentrations of oligosaccharides on phagocytic activity favored mainly the higher (100 μg/ml) concentration. After 24 h exposure, the highest phagocytic activity was observed for heptasaccharides **30** and **27** (1.7-fold increases compared with the control). After 48 h exposure, heptasaccharide **30** remained the most effective inducer of phagocytic activity of the chitin-derived oligosaccharides; however, the higher concentration of all tested chitosan-derived oligosaccharides showed comparable inductions of increased phagocytic activity.

**Table 1:** Evaluation of phagocytosis, respiratory burst, and metabolic activity of RAW 264.7 cells following exposure to chitin and chitin-derived oligosaccharides.

**Formulae**	**Dose (μg/ml)**	**Cell bound and internalized SPA (%)**	**Internalized SPA (%)**	**Membrane attached SPA (%)**	**Respiratory burst (%)**	**Metabolic activity (%)**
**RAW 264.7 24 h exposition**						
**28**	10	50.17 ± 0.08^***^	33.95 ± 0.16^**^	16.22 ± 0.26^*^	46.25 ± 0.02^***^	21.77 ± 0.04^***^
	100	68.72 ± 0.15^***^	38.15 ± 0.14^***^	30.57 ± 0.17^***^	68.59 ± 0.11^***^	26.55 ± 0.10^***^
**29**	10	63.17 ± 0.14^***^	42.92 ± 0.10^***^	20.25 ± 0.13^**^	58.51 ± 0.11^***^	27.76 ± 0.08^***^
	100	73.49 ± 0.01^***^	43.07 ± 0.21^***^	30.42 ± 0.21^***^	62.39 ± 0.12^***^	31.72 ± 0.13^***^
**30**	10	65.54 ± 0.15^***^	49.29 ± 0.14^***^	16.25 ± 0.23^**^	66.11 ± 0.17^***^	37.00 ± 0.15^***^
	100	80.52 ± 0.11^***^	37.81 ± 0.06^***^	42.71 ± 0.13^***^	53.49 ± 0.19^***^	43.90 ± 0.07^***^
Chitin	10	40.68 ± 0.08^***^	33.41 ± 0.14^**^	7.27 ± 0.10^***^	60.18 ± 0.24^***^	28.78 ± 0.09^***^
	100	67.62 ± 0.09^***^	44.14 ± 0.12^***^	23.45 ± 0.10^***^	63.44 ± 0.02^***^	35.37 ± 0.02^***^
Control		47.62 ± 0.04	29.65 ± 0.14	17.97 ± 0.06	39.62 ± 0.26	23.64 ± 0.01
**RAW 264.7 48 h exposition**						
**28**	10	46.24 ± 0.18^***^	21.37 ± 0.07^***^	24.87 ± 0.12^**^	53.2 ± 0.08	21.47 ± 0.06^***^
	100	74.24 ± 0.51^***^	37.56 ± 0.03^***^	36.68 ± 0.12^***^	93.98 ± 0.08^***^	28.15 ± 0.12^*^
**29**	10	43.41 ± 0.26^***^	16.29 ± 0.26^***^	27.12 ± 0.14*ns*	42.92 ± 0.29^***^	8.10 ± 0.01^***^
	100	66.83 ± 0.12^***^	16.70 ± 0.32^***^	50.13 ± 0.18^***^	69.43 ± 0.20^***^	26.68 ± 0.05^***^
**30**	10	74.19 ± 0.48^***^	5.70 ± 0.24^***^	68.49 ± 0.26^***^	77.34 ± 0.35^***^	16.87 ± 0.09^***^
	100	84.30 ± 0.04^***^	6.30 ± 0.10^***^	78.00 ± 0.03^***^	90.93 ± 0.15^***^	17.90 ± 0.04^***^
Chitin	10	64.06 ± 0.23^***^	5.70 ± 0.05^***^	58.36 ± 0.18^***^	44.93 ± 0.11^***^	10.47 ± 0.02^***^
	100	78.83 ± 0.25^***^	7.41 ± 0.03^***^	71.00 ± 0.05^***^	54.25 ± 0.12^*^	23.96 ± 0.12^***^
Control		54.14 ± 0.15	27.18 ± 0.13	26.96 ± 0.26	53.43 ± 0.13	28.76 ± 0.04

Phagocytic activity, oxidative burst, and metabolic activity were expressed as percentage of cells actually undergoing these processes in a population of 10,000 cells. All data are presented as mean ± SD. Tests were carried out in triplicate. Statistical significance of differences between untreated and stimulated cells using one-way ANOVA and post-hoc Bonferroni tests is expressed as:

*** *P < 0.001*,

** *0.001 < P < 0.01*,

* *0.01 < P < 0.05*.

**Table 2:** Evaluation of phagocytosis, respiratory burst, and metabolic activity of RAW 264.7 cells following exposure to chitosan and chitosan-derived oligosaccharides.

**Formulae**	**Dose (μg/ml)**	**Cell bound and internalized SPA(%)**	**Internalized SPA (%)**	**Membrane attached SPA (%)**	**Respiratory burst (%)**	**Metabolic activity (%)**
**RAW 264.7 24 h exposition**						
**25**	10	54.19 ± 0.01^***^	52.44 ± 0.01^***^	1.75 ± 0.04^***^	68.06 ± 0.33^***^	26.2 ± 0.03^***^
	100	58.13 ± 0.01^***^	42.61 ± 0.21^***^	15.52 ± 0.04^***^	62.56 ± 0.02^***^	25.86 ± 0.03^***^
**26**	10	54.01 ± 0.12^***^	36.77 ± 0.05^***^	17.24 ± 0.31^*^	69.92 ± 0.06^***^	28.13 ± 0.08^***^
	100	51.12 ± 0.13^***^	30.68 ± 0.07^*^	20.44 ± 0.09^***^	62.39 ± 0.12^***^	31.72 ± 0.08^***^
**27**	10	70.28 ± 0.15^***^	42.54 ± 0.06^***^	27.75 ± 0.11^***^	49.31 ± 0.10^***^	25.19 ± 0.07^***^
	100	80.52 ± 0.11^***^	32.91 ± 0.21^**^	42.71 ± 0.24^***^	66.92 ± 0.01^***^	25.71 ± 0.07^***^
Chitosan	10	60.12 ± 0.11^***^	38.91 ± 0.24^***^	21.21 ± 0.16^**^	58.74 ± 0.01^***^	24.11 ± 0.09^*^
	100	66.12 ± 0.21^***^	36.42 ± 0.32^***^	29.77 ± 0.13^**^	63.82 ± 0.02^***^	35.16 ± 0.23^***^
Control		47.62 ± 0.04	29.65 ± 0.14	17.97 ± 0.06	39.65 ± 0.26	23.64 ± 0.01
**RAW 264.7 48 h exposition**						
**25**	10	74.24 ± 0.26^***^	69.23 ± 0.24^***^	5.01 ± 0.15^***^	65.8 ± 0.14^***^	37.55 ± 0.26^***^
	100	89.30 ± 0.32^***^	68.2 ± 0.26^***^	21.10 ± 0.11^**^	81.3 ± 0.05^***^	22.36 ± 0.08^***^
**26**	10	60.64 ± 0.40^***^	57.22 ± 0.40^***^	3.42 ± 0.32^***^	71.60 ± 0.12^***^	37.46 ± 0.23^***^
	100	72.05 ± 0.26^***^	46.45 ± 0.10^***^	26.6 ± 0.23	93.33 ± 0.26^***^	24.60 ± 0.12^***^
**27**	10	46.30 ± 0.26^***^	59.35 ± 0.06^***^	13.05 ± 0.24^***^	89.06 ± 0.05^***^	16.60 ± 0.09^***^
	100	70.01 ± 0.34^***^	57.72 ± 0.09^***^	12.29 ± 0.17^***^	70.18 ± 0.15^***^	26.01 ± 0.05^***^
Chitosan	10	62.28 ± 0.37^***^	19.95 ± 0.23^***^	42.33 ± 0.27^***^	68.35 ± 0.03^***^	15.90 ± 0.23^***^
	100	78.30 ± 0.15^***^	17.21 ± 0.05^***^	61.09 ± 0.11^***^	83.98 ± 0.23^***^	17.21 ± 0.11^***^
Control		54.14 ± 0.15	27.18 ± 0.13	26.96 ± 0.26	53.43 ± 0.13	28.76 ± 0.04

Phagocytic activity, oxidative burst, and metabolic activity were expressed as percentage of cells actually undergoing these processes in a population of 10,000 cells. All data are presented as mean ± SD. Tests were carried out in triplicate. Statistical significance of differences between untreated and stimulated cells using one-way ANOVA and post-hoc Bonferroni tests is expressed as:

*** *P < 0.001*,

** *0.001 < P < 0.01*,

* *0.01 < P < 0.05*.

**Table 3:** Evaluation of phagocytosis, respiratory burst, and metabolic activity of RAW 264.7 cells following exposure to cell mitogens.

**Formulae**	**Cell bound and internalized SPA (%)**	**Internalized SPA (%)**	**Membrane attached SPA (%)**	**Respiratory burst (%)**	**Metabolic activity (%)**
**24 h exposition**					
ConA	68.88 ± 0.14^***^	49.45 ± 0.06^***^	19.43 ± 0.16^**^	53.63 ± 0.02^***^	24.84 ± 0.06^**^
PWM	62.20 ± 0.16^***^	43.51 ± 0.13^***^	18.69 ± 0.24	57.62 ± 0.03^***^	26.44 ± 0.06^***^
PHA	48.08 ± 0.05^*^	40.51 ± 0.20^***^	7.56 ± 0.16^***^	67.46 ± 0.04^***^	22.80 ± 0.02^***^
LPS	54.88 ± 0.26^***^	43.17 ± 0.22^***^	11.71 ± 0.26^***^	70.37 ± 0.22^***^	30.14 ± 0.13^***^
**48 h exposition**					
ConA	52.37 ± 0.24^*^	38.23 ± 0.11^***^	14.14 ± 0.13^***^	41.39 ± 0.12	22.35 ± 0.16^***^
PWM	52.38 ± 0.24^*^	32.25 ± 0.20^**^	20.13 ± 0.19^**^	46.35 ± 0.15^***^	21.33 ± 0.12^***^
PHA	35.16 ± 0.25^***^	30.90 ± 0.15^***^	4.26 ± 0.17^***^	47.22 ± 0.21^***^	30.20 ± 0.12^**^
LPS	44.23 ± 0.19^***^	35.88 ± 0.11^***^	8.35 ± 0.27^***^	63.23 ± 0.13^***^	38.79 ± 0.26^***^

Phagocytic activity, oxidative burst, and metabolic activity were expressed as percentage of cells actually undergoing these processes in a population of 10,000 cells. All data are presented as mean ± SD. Tests were carried out in triplicate. Statistical significance of differences between untreated and stimulated cells using one-way ANOVA and post-hoc Bonferroni tests is expressed as:

*** *P < 0.001*,

** *0.001 < P < 0.01*,

* *0.01 < P < 0.05*.

Although increased phagocytic activity was induced by longer oligosaccharides (**30**−24 h and 48 h, **27**−24 h) and higher concentration (100 μg/ml), SPA-FITC particles remained mostly attached to the cell membrane. Higher internalization was induced by shorter oligosaccharides and/or lower concentration (10 μg/ml). Exposure of RAW 264.7 cells to chitosan-derived oligosaccharides for 48 h increased internalization of SPA-FITC particles for all tested oligosaccharides (**25**, **26**, and **27**) and for both concentrations. Exposure to all tested chitin- and chitosan-derived oligosaccharides enhanced the respiratory burst of RAW 264.7 macrophages after 24 h (from 1.2- to 1.8-fold increases). The 48 h treatment demonstrated that the higher concentration of chitin-derived oligosaccharides more effectively induced the respiratory burst of RAW 264.7 macrophages than the lower concentration.

#### Cytokine Responses of RAW 264.7 Macrophages *in vitro* to Chitin- and Chitosan-Derived Oligosaccharides

**Figure 2:**
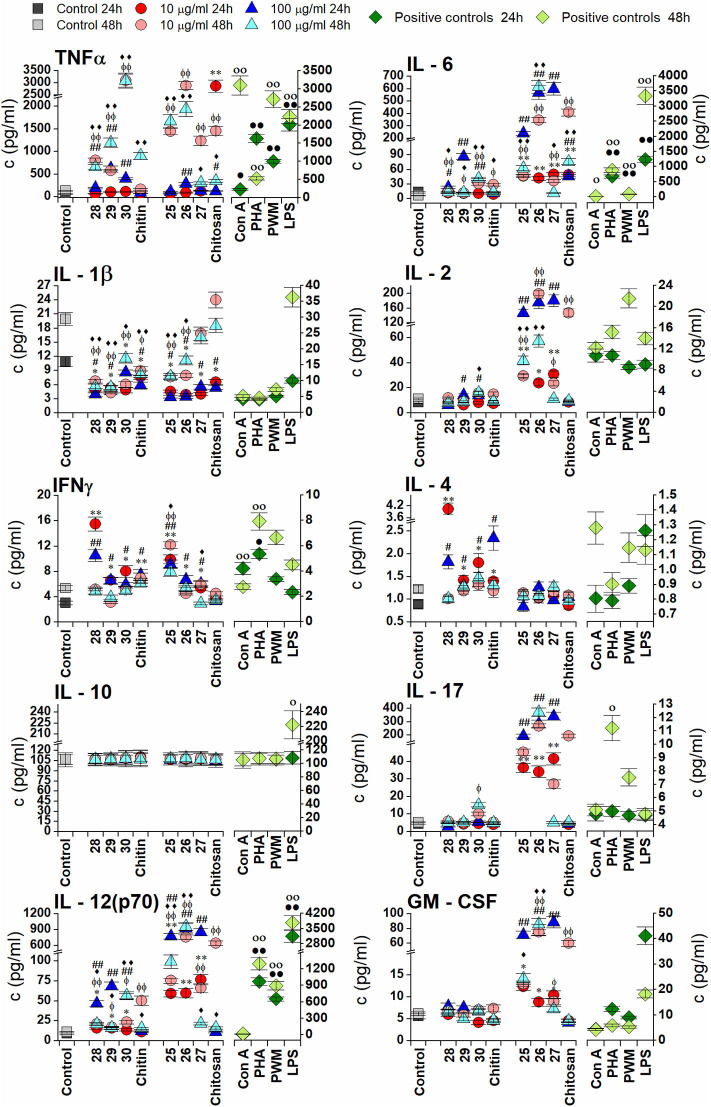
Effect of chitin oligosaccharide and chitosan oligosaccharide glycoconjugates on RAW 264.7 macrophagescytokines production. Concentrations of cytokines (pg/ml) in media were determined after stimulation of RAW264.7 macrophages for 24 or 48 h with 10 μg/ml or 100 μg/ml of chitin oligosaccharide glycoconjugates: (GNAc)_3_ - Spacer - Biotin **(1a)**, (GNAc)_5_ - Spacer - Biotin **(2a)**, (GNAc)_7_ - Spacer - Biotin **(3a)**; Chitin; chitosan oligosaccharide glycoconjugates: (GNH2)_3_ - Spacer - Biotin **(1b)**, (GNH2)_5_ - Spacer - Biotin **(2b)**, (GNH2)_7_ - Spacer - Biotin **(3b)** and Chitosan. Negative control represents untreated RAW 264.7 cells (Control). Concanavaline A (Con A, 10 μg/ml) and phytohemagglutinin (PHA, 10 μg/ml) Pokeweed mitogen, (PWM, 1 μg/ml) and lipopolysaccharide (LPS, 10 μg/m)were used as positive controls. All data are presented as Mean ± SD. Tests were carried out in triplicate. The statistical significance of differences between untreated cells and stimulated cells are expressed; 24 h treatment with 10 μg/ml of oligosaccharide glycoconjugate: ***P < 0.001, **0.001 < *P* < 0.01, *0.01 < *P* < 0.05, 24 h treatment with 100 μg/ml of oligosaccharide glycoconjugate: ^*###*^*P* < 0.001, ^*##*^0.001 < *P* < 0.01, ^#^0.01 < *P* < 0.05, 48 h treatment with 10 μg/ml of oligosaccharide glycoconjugate: ^Φ*ΦΦ*^ *P* < 0.001, ^ΦΦ^0.001 < *P* < 0.01, ^Φ^0.01 < *P* < 0.05 and 48 h treatment with 100 μg/ml of oligosaccharide glycoconjugate: ^♦♦♦^*P* < 0.001, ^♦♦^0.001 < *P* < 0.01, ^♦^0.01 < *P* < 0.05. The statistical significance of differences between untreated cells and positive controls are expressed; 24 h treatment:^•••^*P* < 0.001,^••^0.001 < *P* < 0.01,^•^0.01 < *P* < 0.05, 48 h treatment: ^○○○^*P* < 0.001, ^○○^0.001 < *P* < 0.01, ^○^0.01 < *P* < 0.05.

Chitin- and chitosan-derived oligosaccharides were examined for their effect on the production of pro-inflammatory cytokines (TNFα, IL-6, IL-1β, IL-2, IL-17, and IL-12), anti-inflammatory cytokine IL-10, and hemopoietic growth factor GM-CSF in RAW 264.7 macrophages after exposure for 24 or 48 h (Figure 2). All tested oligosaccharides induced increases in TNFα and IL-6 production; however, chitin-derived oligosaccharides **28**, **29**, and **30** induced a more pronounced increase in TNFα production (24 h exposure with 100 μg/ml of **29**−10.0-fold, 48 h exposure with 10, and 100 μg/ml of **30**−23.2-fold). Chitosan-derived oligosaccharides induced a marked increase in IL-6 production (24 h exposure with 100 μg/ml of **26**−44.6-fold and **27**−46.8-fold; 48 h exposure with 10 μg/ml of **26**−57.0-fold and 100 μg/ml of **26**−101.0-fold).

Chitosan-derived oligosaccharides **25**, **26**, and **27** induced significantly increased production of IL-2 with maximal efficacy after 24 h exposure with 100 μg/ml (**25**−17.1-fold, *P* = 0.0028, **26**−20.6-fold, *P* = 0.0054, and **27**−21.1-fold, *P* = 0.004). Exposure of RAW 264.7 cells to the shortest chitin oligosaccharide **28** for 24 h induced significantly increased production of IL-4 (10 μg/ml−4.5-fold, *P* = 0.0045 and 100 μg/ml−2.1-fold, *P* = 0.018). Markedly increased IFNγ production was induced after 24 h exposure with the shortest oligosaccharides of both types (**28**: 10 μg/ml−5.1-fold, 100 μg/ml−3.5-fold and **25**: 10 μg/ml−3.3-fold, 100 μg/ml−3.0-fold). Stimulation of RAW 264.7 cells with chitin- or chitosan-derived oligosaccharides did not significantly increase IL-1β and IL-10 production (Figure 2).

Similar to IL-2 production, stimulation with chitosan-derived oligosaccharides **25**, **26**, and **27** significantly increased IL-17 production, induced markedly increased IL-12 production, and increased GM-CSF production compared with chitin-derived oligosaccharides **28**, **29**, and **30**. Production of IL-17 was most effectively induced after 24 h with 100 μg/ml of all chitosan oligosaccharides (**25**−42.2-fold, **26**−62.0-fold, and **27**−74.9-fold), and after 48 h for both concentrations of **26** (10 μg/ml−50.7-fold, 100 μg/ml−70.4-fold). The highest increases in IL-12 and GM-CSF production were observed after 24 h exposure to 100 μg/ml of chitosan-derived oligosaccharides (IL-12: **25**−84.7-fold, **26**−101.1-fold, and **27**−93.3-fold; GM-CSF: **25**−12.8-fold, **26**−14.0-fold, and **27**−15.8-fold). Significant cross-correlations were observed between IL-6, IL-17, IL-12, and GM-CSF production when chitosan-derived oligomers **25**, **26**, and **27** were applied at different concentrations for 48 h (Table 4).

**Table 4:** Pattern of cytokine dose-dependent cross-correlations.

**Cytokines**	**Dose 10 μg/ml**	**Dose 100 μg/ml**
IL-6 and IL-17	*R =* 0.999, *P =* 0.023	*R =* 0.999, *P =* 0.027
IL-6 and IL-12	*R =* 0.999, *P =* 0.012	*R =* 0.999, *P =* 0.001
IL-6 and GM-CSF	*R =* 0.999, *P =* 0.016	*R =* 0.999, *P =* 0.002
IL-17 and IL-12	*R =* 0.998, *P =* 0.035	*R =* 0.998, *P =* 0.029
IL-17 and GM-CSF	*R =* 0.999, *P =* 0.008	*R =* 0.999, *P =* 0.026
IL-12 and GM-CSF	*R =* 0.999, *P =* 0.028	*R =* 0.999, *P =* 0.003

## Discussion

In this study, an array of synthetically prepared chitin- and chitosan-derived oligosaccharides of different lengths was examined to elucidate their influence on the behavior of macrophage RAW 264.7 cells. To evaluate the relationship between the chito-oligosaccharide structure and immunobiological activity, functional tests based on the induction of various immune responses, including proliferation, cytotoxicity, immunological inflammation, and polarization of T-helper responses (Th1, Th2, Th17, or Treg), were applied following RAW 264.7 cell exposure to natural *Candida* chitin and chitosan and synthetic oligosaccharide fragments **28**–**30** and **25**–**27**.

Proliferation results after exposure for 24 and 48 h demonstrated good biocompatibility without cytotoxic effects for all tested formulas, even at the higher concentration (Figure 1). Chitosan-derived structures more effectively promoted and accelerated macrophage cell proliferation compared with chitin derivatives (Figure 1), which concurred with results from other studies. Yang et al. (2019) revealed good cytocompatibility of chito-oligosaccharides after pretreatment of RAW 264.7 cells for 24 h, even at 100 μg/ml concentration. Active stimulation and enhancement of cell proliferation was reported with chitosan and chitosan-derived structures in different cell systems, such as human skin fibroblasts and keratinocytes (Howling et al., 2001), neuron-like PC12 cells (Alhosseini et al., 2012), Saos-2 cells (Isikli et al., 2012), and L929 fibroblasts (Tangsadthakun et al., 2007). Chang et al. (2019) revealed significantly increased mitogen-induced proliferation of splenocytes and Peyer's patch lymphocytes after *in vivo* administration of chitosan hydrolytic products in mice. Hoseini et al. (2016) determined that chitin and chitosan microparticles (<40 μm) subcutaneously injected into Balb/c mice induced cell proliferation.

Generally, macrophages are essential members of the phagocyte cell system and are essential to cellular innate immune responses, as they are programmed to recognize, engulf, and destroy immune complexes, foreign particles, bacteria, mycobacteria, apoptotic cells, etc. They initiate the process of phagocytosis, accompanied by the release of cytokines and chemokines, which contribute to inflammatory regulation (Artis and Spits, 2015). Macrophages are polarized based on their activation to the pro-inflammatory M1 phenotype (classic activation) and anti-inflammatory M2 phenotype (alternative activation). Various phagocytosis patterns were observed with exposure to natural *Candida* chitin, chitosan, and chitin- and chitosan-derived oligosaccharides **28**–**30** and **25**–**27**, especially those concerning ingestion and internalization of bacteria particles, together with subsequent respiratory burst induction, which supported the impact of oligosaccharide size on macrophage immune enhancement. Harish Prashanth and Tharanathan (2007) revealed greater immunopotentiation activity of chito-oligosaccharides with chain lengths greater than six, compared with smaller chito-oligosaccharides. Fuchs et al. (2018) observed that immune recognition of defined chitin oligomers depends on a minimum number of 6–7 *N*-acetyl-d-glucosamine subunits in both human and murine cells. Further, mannose receptors have sensed RAW 264.7 cell interaction with chitosan oligomers comprising 3–10 saccharide (*N*-acetyl-d-glucosamine or glucosamine) residues (Han et al., 2005).

Enhanced proliferation and neutral red phagocytosis by RAW 264.7 macrophages has been observed with chitosan oligosaccharides derived from hydrolyzed chitosan with 3–8 degrees of polymerization (Zhang et al., 2014). This study also revealed potent immune-stimulating properties of chito-oligosaccharides by activating TLR4 on macrophages. Davis et al. (2018) reported different macrophage activation by non-phagocytosable particles larger than a macrophage, which induced M2 activation, whereas phagocytosable chitin microparticles (1–10 μm diameters) induced M1 macrophage activation. Conversely, chitosan microparticles (1–10 μm) induced poor M1 activation. These results are indicative of acetyl group significance in the phagocytosis-dependent role of M1 activation. Bueter et al. (2011, 2014) suggested the potential role of deacetylated polysaccharide chitosan, contrary to chitin, in the activation of the NLRP3 inflammasome in a phagocytosis-dependent manner.

Cytokines are crucial modulators of immune responses through a complex network of target immune cell interactions and are engaged in various immune system processes, such as activation, proliferation, signalization, polarization, phagocytosis, and inflammation. Generally, they are efficient multipurpose indicators of character and intensity of immunocompetent cell reactivity. Chitin, chitosan, and related oligosaccharides are reportedly effective immunomodulators and cytokine production triggers (Da Silva et al., 2009; Mori et al., 2012; Alvarez, 2014; Xia et al., 2015; Davydova et al., 2016; Elieh Ali Komi et al., 2018). Classification of cytokines by immune response (Turner et al., 2014) revealed the intense dose- and structure-dependent stimulation of cytokines IL-2, IL-4, and GM-CSF engaged in adaptive immunity; IL-1β, IL-6, TNFα, IL-17, and IFNγ linked to pro-inflammatory signaling; and IL-12 and IL-10 with anti-inflammatory signaling for chitosan- and chitin-derived oligosaccharides **25**–**27** and **28**–**30** (Figure 2). Exposure of RAW 264.7 cells to chitin- and chitosan-related compounds exerted pro-Th1 and pro-Th17 responses over pro-Th2 polarization, based on cytokine release values of Th1 (IFNγ) > Th2 (IL-4) and Th17 (IL-17) > Th2 (IL-4).The most significant increases in IL-6, TNFα, IL-2, IL-17, IL-12 (p70), IL-10, and GM-CSF were observed with exposure to chitosan-derived oligosaccharides **25**–**27** and *Candida* chitosan. On the contrary, exposure to chitin-derived oligosaccharides **28**–**30** and *Candida*-derived chitin resulted in time- and concentration-dependent increases only in IL-10, IL-4, and TNFα.

Chitin, chitosan, and their derivatives may be potentially employed in immunomodulating adjuvant formulas based on their ability to affect macrophage polarization to pro-inflammatory M1 phenotype induced by inflammatory cytokines, such as TNFα and IFNγ, polarization to anti-inflammatory M2 phenotype induced by anti-inflammatory cytokines, such as IL-10, IL-4, and IL-13, as well as the release of relevant cytokine patterns. Research by Da Silva et al. (2010) demonstrated that chitin is a potent adjuvant that augments Th2, Th1, and Th17 responses *in vivo* and *in vitro*. Mori et al. (2012) revealed that a combination of chitosan and TLR9 agonist CpG as an adjuvant activated the NLRP3 inflammasome and enhanced secretion of IL-12 and the other key Th1 and Th17 cell-polarizing cytokines. Further, Jesus et al. (2018) characterized adjuvant activity of poly-ε-caprolactone/chitosan nanoparticles and mastocyte activation accompanied by IFN-γ and IL-17 release. Particularly of interest is the inhibition of LPS-induced inflammatory cytokines TNF-α, IL-1, and IL-6 in RAW 264.7 macrophage cells by either chitosan nanoparticles (Ma et al., 2016) or chitosan oligosaccharides (Yoon et al., 2007). Chang et al. (2019) demonstrated in RAW 264.7 macrophage cells that 156 and 72 kDa chitosans significantly inhibited the production of TNFα and IL-6, whereas 7.1 kDa chitosan and chito-oligosaccharides significantly induced their production.

Evidently, chitosan particle size played an important role, with small particles eliciting the greatest activity. This concurs with a similar pattern regarding size-dependent production of macrophage TNF and IL-10 observed by Da Silva et al. (2009). Their study revealed that large chitin fragments were inert, whereas intermediate-sized chitin (40–70 μm) and small chitin (<40 μm, largely 2–10 μm) stimulated TNF expansion. In contrast, only small chitin induced IL-10 expansion. Shibata et al. (1997) observed the induction of IL-12, TNFα, and IFNγ in mouse splenocytes with phagocytosable-size chitin particles (1–10 μm), whereas the release of these cytokines was not observed with larger particles (50–100 μm). Co-formulation of chitosan with IL-12 has been suggested as an effective antitumor immunotherapy (Heffernan et al., 2011; Smith et al., 2015). Additionally, antitumor efficacy has been enhanced by co-delivery of doxorubicin and IL-2 using chitosan-based nanoparticles (Wu et al., 2017).

## Conclusions

Biotinylated chitosan oligomers comprising 3, 5, and 7 glucosamine residues were efficiently synthesized. Oligosaccharide chain elongation was accomplished using a disaccharide thioglycoside donor bearing a temporary *tert*-butyldimethylsilyl group at O-4′. The disaccharide donor was prepared in a straightforward manner by orthogonal glycosylation of a thioglycoside acceptor with 4-*O*-TBDMS trichloroacetimidate. In contrast to glycosylation with 4-*O*-acyl-donors that was accompanied by intensive aglycon transfer, application of the silyl-protected donor almost completely obviated this side reaction. *In vitro* immunobiological evaluation of chitin- and chitosan-derived oligosaccharides (**28**–**30** and **25**–**27**, respectively) in RAW 264.7 cells demonstrated effective immunomodulation with respect to induction of cytokine release, cell proliferation, phagocytosis, and respiratory burst. Macrophage reactivity was accompanied by significant inductive concentration- and structure-dependent Th1 and Th17 polarization, including increased Th1 cytokine production for IL-2, IL-12 (p70), TNFα, GM-CSF, and Th17 cytokine IL-17, which was greater with exposure to chitosan- rather than chitin-derived oligosaccharides. Tested oligomers triggered significant cell release of anti-inflammatory IL-10 and were efficient inducers of internalization and phagocytosis of *S. aureus* particles by RAW 264.7 cells. Moreover, the absence of antiproliferative/cytotoxic effects, even following prolonged 48 h exposure, is a promising result for further studies with these oligosaccharides. Synthetically prepared chitin- and chitosan-derived oligosaccharides are suitable for use *in vitro* and prospectively *in vivo* for further immunobiological and immunotoxicological studies, as potential antigens for *in vitro* diagnostics of candidosis, and for anti-fungal therapy monitoring.

## Data Availability Statement

All datasets generated for this study are included in the article/**Supplementary Material**.

## Author Contributions

EP, LP, and NN contributed to the conception and design of the study, performed the immunobiological research, analyzed the data, acquired funding, and prepared the original draft. PF performed the modification and characterization of chitin and chitosan. YT performed the chemical syntheses, analyzed the data, and prepared the original draft. All authors contributed to manuscript revision and read and approved the submitted version.

## Conflict of Interest

The authors declare that the research was conducted in the absence of any commercial or financial relationships that could be construed as a potential conflict of interest.

## References

[B1] Alhosseini S. N. Moztarzadeh F. Mozafari M. Asgari S. Dodel M. Samadikuchaksaraei A.. (2012). Synthesis and characterization of electrospun polyvinyl alcohol nanofibrous scaffolds modified by blending with chitosan for neural tissue engineering. Int. J. Nanomed. 7, 25–34. 10.2147/IJN.S25376 22275820 PMC3260948

[B2] Alvarez F. J. (2014). The effect of chitin size, shape, source and purification method on immune recognition. Molecules 19, 4433–4451. 10.3390/molecules19044433 24727416 PMC6271096

[B3] Artis D. Spits H. (2015). The biology of innate lymphoid cells. Nature 517, 293–301. 10.1038/nature14189 25592534

[B4] Baker L. G. Specht C. A. Donlin M. J. Lodge J. K. (2007). Chitosan, the deacetylated form of chitin, is necessary for cell wall integrity in *Cryptococcus neoformans*. Eukaryot. Cell 6, 855–867. 10.1128/EC.00399-06 17400891 PMC1899242

[B5] Baker L. G. Specht C. A. Lodge J. K. (2011). Cell wall chitosan is necessary for virulence in the opportunistic pathogen *Cryptococcus neoformans*. Eukaryot. Cell 10, 1264–1268. 10.1128/EC.05138-11 21784998 PMC3187048

[B6] Barry C. S. Cocinero E. J. Çarçabal P. Gamblin D. P. E Stanca-Kaposta C.. (2013). ‘Naked’ and hydrated conformers of the conserved core pentasaccharide of N-linked glycoproteins and its building blocks. J. Am. Chem. Soc. 135, 16895–16903. 10.1021/ja4056678 24127839 PMC3901393

[B7] Bastiaens L. Soetemans L. D'Hondt E. Elst K. (2019). Sources of Chitin and chitosan and their isolation, in Chitin and Chitosan: Properties and Applications, eds. Broek L. A. M. v. d. Boeriu C. G. (Chichester: John Wiley & Sons, Ltd), 1–34. 10.1002/9781119450467.ch1

[B8] Bowman S. M. Free S. J. (2006). The structure and synthesis of the fungal cell wall. BioEssays, 28, 799–808. 10.1002/bies.20441 16927300

[B9] Bueter C. L. Lee C. K. Rathinam V. A. Healy G. J. Taron C. H. Specht C. A.. (2011). Chitosan but not chitin activates the inflammasome by a mechanism dependent upon phagocytosis. J. Biol. Chem. 286, 35447–35455. 10.1074/jbc.M111.274936 21862582 PMC3195641

[B10] Bueter C. L. Lee C. K. Wang J. P. Ostroff G. R. Specht C. A. Levitz S. M. (2014). Spectrum and mechanisms of inflammasome activation by chitosan. J. immunol. 192, 5943–5951. 10.4049/jimmunol.1301695 24829412 PMC4063524

[B11] Bueter C. L. Specht C. A. Levitz S. M. (2013). Innate sensing of chitin and chitosan. PLoS pathog. 9:e1003080. 10.1371/journal.ppat.1003080 23326227 PMC3542151

[B12] Cartmell J. Paszkiewicz E. Dziadek S. Tam P. H. Luu T. Sarkar S. (2015). Synthesis of antifungal vaccines by conjugation of β-1,2 trimannosides with T-cell peptides and covalent anchoring of neoglycopeptide to tetanus toxoid. Carbohydr. Res. 403, 123–134. 10.1016/j.carres.2014.06.024 25126994

[B13] Chang S. H. Lin Y. Y. Wu G. J. Huang C. H. Tsai G. J. (2019). Effect of chitosan molecular weight on anti-inflammatory activity in the RAW 264.7 macrophage model. Int. J. Biol. Macromol. 131, 167–175. 10.1016/j.ijbiomac.2019.02.066 30771390

[B14] Colombo C. Pitirollo O. Lay L. (2018). Recent advances in the synthesis of glycoconjugates for vaccine development. Molecules 23:1712. 10.3390/molecules23071712 30011851 PMC6099631

[B15] Costello C. Bundle D. R. (2012). Synthesis of three trisaccharide congeners to investigate frame shifting of β1,2-mannan homo-oligomers in an antibody binding site. Carbohydr. Res. 357, 7–15. 10.1016/j.carres.2012.03.019 22658565

[B16] Cunha L. Rodrigues S. Rosa da Costa A. M. Faleiro L. Buttini F. Grenha A. (2019). Inhalable chitosan microparticles for simultaneous delivery of isoniazid and rifabutin in lung tuberculosis treatment. Drug Dev. Ind. Pharm. 45, 1313–1320. 10.1080/03639045.2019.1608231 30990096

[B17] Czechowska-Biskup R. Jarosinska D. Rokita B. Ulanski P. Rosiak J. M. (2012). Determination of degree of deacetylation of chitosan - comparision of methods. Prog. Chem. Appl. Chitin Deriv. 17, 5–20.

[B18] Da Silva C. A. Chalouni C. Williams A. Hartl D. Lee C. G. Elias J. A. (2009). Chitin is a size-dependent regulator of macrophage TNF and IL-10 production. J. Immunol. 182, 3573–3582. 10.4049/jimmunol.0802113 19265136

[B19] Da Silva C. A. Pochard P. Lee C. G. Elias J. A. (2010). Chitin particles are multifaceted immune adjuvants. Am. J. Respir. Crit. Care Med. 182, 1482–1491. 10.1164/rccm.200912-1877OC 20656945 PMC3029935

[B20] Dang A. T. Johnson M. A. Bundle D. R. (2012). Synthesis of a Candida albicans tetrasaccharide spanning the β1,2-mannan phosphodiester α-mannan junction. Org. Biomol. Chem. 10, 8348–8360. 10.1039/c2ob26355f 22996034

[B21] Davis S. Cirone A. M. Menzie J. Russell F. Dorey C. K. Shibata Y.. (2018). Phagocytosis-mediated M1 activation by chitin but not by chitosan. Am. J. Physiol. Cell Physiol. 315, C62–C72. 10.1152/ajpcell.00268.2017 29719169 PMC6087726

[B22] Davydova V. N. Kalitnik A. A. Markov P. A. Volod'ko A. V. Popov S. V. Ermak I. M. (2016). Cytokine-inducing and anti-inflammatory activity of chitosan and its low-molecular derivative. Appl. Biochem. Microbiol. 52, 460–466. 10.1134/S0003683816050070 29513410

[B23] de Carvalho F. G. Magalhaes T. C. Teixeira N. M. Gondim B. L. C. Carlo H. L. Dos Santos R. L.. (2019). Synthesis and characterization of TPP/chitosan nanoparticles: colloidal mechanism of reaction and antifungal effect on *C. albicans* biofilm formation. Mater. Sci. Eng. 104:109885. 10.1016/j.msec.2019.109885 31500048

[B24] Elieh Ali Komi D. Sharma L. Dela Cruz C. S. (2018). Chitin and Its Effects on Inflammatory and Immune Responses. Clin. Rev. Allergy Immunol. 54, 213–223. 10.1007/s12016-017-8600-0 28251581 PMC5680136

[B25] Erwig L. P. Gow N. A. R. (2016). Interactions of fungal pathogens with phagocytes. Nat. Rev. Microbiol. 14, 163–176. 10.1038/nrmicro.2015.21 26853116

[B26] Felt O. Buri P. Gurny R. (1998). Chitosan: a unique polysaccharide for drug delivery. Drug Dev. Iind. Pharm. 24, 979–993. 10.3109/03639049809089942 9876553

[B27] Ferreira C. Silva S. van Voorst F. Aguiar C. Kielland-Brandt M. C. Brandt A.. (2006). Absence of Gup1p in *Saccharomyces cerevisiae* results in defective cell wall composition, assembly, stability and morphology. FEMS Yeast Res. 6, 1027–1038. 10.1111/j.1567-1364.2006.00110.x 17042752

[B28] Free S. J. (2013). Fungal cell wall organization and biosynthesis. Adv. Genet. 81, 33–82. 10.1016/B978-0-12-407677-8.00002-6 23419716

[B29] Fuchs K. Cardona Gloria Y. Wolz O. O. Herster F. Sharma L. Dillen C. A.. (2018). The fungal ligand chitin directly binds TLR2 and triggers inflammation dependent on oligomer size. EMBO Rep. 19:e46065. 10.15252/embr.201846065 30337494 PMC6280652

[B30] Geoghegan I. A. Gurr S. J. (2016). Chitosan mediates germling adhesion in *Magnaporthe oryzae* and is required for surface sensing and germling morphogenesis. PLoS Pathog. 12:e1005703. 10.1371/journal.ppat.1005703 27315248 PMC4912089

[B31] Gow N. A. R. Hube B. (2012). Importance of the Candida albicans cell wall during commensalism and infection. Curr. Opin. Microbiol. 15, 406–412. 10.1016/j.mib.2012.04.005 22609181

[B32] Gow N. A. R. Latgé J. P. Munro C. A. (2017). The fungal cell wall: structure, biosynthesis, and function. Microbiol. Spectrum 5:FUNK-0035-2016. 10.1128/9781555819583.ch12 28513415 PMC11687499

[B33] Han Y. Zhao L. Yu Z. Feng J. Yu Q. (2005). Role of mannose receptor in oligochitosan-mediated stimulation of macrophage function. Int. Immunopharmacol. 5, 15336–11542. 10.1016/j.intimp.2005.04.015 16023605

[B34] Harish Prashanth K. V. Tharanathan R. N. (2007). Chitin/chitosan: modifications and their unlimited application potential—an overview. Trends Food Sci. Technol. 18, 117–131. 10.1016/j.tifs.2006.10.022

[B35] Heffernan M. J. Zaharoff D. A. Fallon J. K. Schlom J. Greiner J. W. (2011). *In vivo* efficacy of a chitosan/IL-12 adjuvant system for protein-based vaccines. Biomaterials 32, 926–932. 10.1016/j.biomaterials.2010.09.058 20965561 PMC2992965

[B36] Hoseini M. H. Moradi M. Alimohammadian M. H. Shahgoli V. K. Darabi H. Rostami A. (2016). Immunotherapeutic effects of chitin in comparison with chitosan against *Leishmania major* infection. Parasitol. Int. 65, 99–104. 10.1016/j.parint.2015.10.007 26518128

[B37] Howling G. I. Dettmar P. W. Goddard P. A. Hampson F. C. Dornish M. Wood E. J. (2001). The effect of chitin and chitosan on the proliferation of human skin fibroblasts and keratinocytes *in vitro*. Biomaterials 22, 2959–2966. 10.1016/S0142-9612(01)00042-4 11575470

[B38] Isikli C. Hasirci V. Hasirci N. (2012). Development of porous chitosan-gelatin/hydroxyapatite composite scaffolds for hard tissue-engineering applications. J. Tissue Eng. Regener. Med. 6, 135–143. 10.1002/term.406 21351375

[B39] Jesus S. Soares E. Borchard G. Borges O. (2018). Adjuvant Activity of Poly-ε-caprolactone/chitosan nanoparticles characterized by mast cell activation and IFN-γ and IL-17 production. Mol. Pharmaceutics 15, 72–82. 10.1021/acs.molpharmaceut.7b00730 29160080

[B40] Karelin A. A. Tsvetkov Y. E. Paulovičová E. Paulovičová L. Nifantiev N. E. (2016). A blockwise approach to the synthesis of (1→2)-linked oligosaccharides corresponding to fragments of the acid-stable β-mannan from the Candida albicans cell wall. Eur. J. Org. Chem. 2016, 1173–1181. 10.1002/ejoc.201501464

[B41] Kato Y. Onishi H. Machida Y. (2005). Contribution of chitosan and its derivatives to cancer chemotherapy. In vivo 19, 301–310. 15796190

[B42] Kazakova E. D. Yashunsky D. V. Krylov V. B. Bouchara J. P. Cornet M Valsecchi I.. (2020). Biotinylatedoligo-α-(1→4)-D-galactosamines and their N-acetylated derivatives:α-stereoselective synthesis and immunology application. J. Am. Chem. Soc. 142, 1175–1179. 10.1021/jacs.9b11703 31913631

[B43] Khoushab F. Yamabhai M. (2010). Chitin research revisited. Marine drugs 8, 1988–2012. 10.3390/md8071988 20714419 PMC2920538

[B44] Kim S. Y. Shon D. H. Lee K. H. (2000). Enzyme-linked immunosorbent assay for detection of chitooligosaccharides. Biosci. Biotechnol. Biochem. 64, 696–701 10.1271/bbb.64.696 10830479

[B45] Kochetkov N. K. Nifant'ev N. E. Backinowsky L. V. (1987). Synthesis of the capsular polysaccharide of *Streptococcus pneumoniae* type 14. Tetrahedron 43, 3109–3121. 10.1016/S0040-4020(01)86852-6 3580006

[B46] Kogiso M. Nishiyama A. Shinohara T. Nakamura M. Mizoguchi E. Misawa Y.. (2011). Chitin particles induce size-dependent but carbohydrate-independent innate eosinophilia. J. Leukocyte Biol. 90, 167–176. 10.1189/jlb.1110624 21447645 PMC3114598

[B47] Komarova B. S. Orekhova M. V. Tsvetkov Y. E. Beau R. Aimanianda V. Latg,é J. P.. (2015). Synthesis of a pentasaccharide and neoglycoconjugates related to fungal α-(1→3)-glucan and their use in the generation of antibodies to trace *Aspergillus fumigatus* cell wall. Chem. Eur. J. 21, 1029–1035. 10.1002/chem.201404770 25376936

[B48] Komarova B. S. Wong S. S. W. Orekhova M. V. Tsvetkov Y. E. Krylov V. B. Beauvais A.. (2018). Chemical synthesis and application of biotinylated oligo-α-(1→3)-d-glucosides to study the antibody and cytokine response against the cell wall α-(1→3)-d-glucan of *Aspergillus fumigatus*. J. Org. Chem. 83, 12965–12976. 10.1021/acs.joc.8b01142 30277398 PMC6461050

[B49] Krylov V. B. Nifantiev N. E. (2020). Synthetic oligosaccharides mimicking fungal cell wall polysaccharides. Curr. Top. Microbiol. Immunol. 425, 1–16. 10.1007/82_2019_187 31875266

[B50] Krylov V. B. Petruk M. I. Glushko N. I. Khaldeeva E. V. Mokeeva V. L. Bilanenko E. N. (2018a). Carbohydrate specificity of antibodies against phytopathogenic fungi of the *Aspergillus* genus. Appl. Biochem. Microbiol. 54, 522–527. 10.1134/S0003683818050095

[B51] Krylov V. B. Petruk M. I. Grigoryev I. V. Lebedin Y. S. Glushko N. I. Khaldeeva E. V. (2018b). Study of the carbohydrate specificity of antibodies against *Aspergillus fumigatus* using the library of synthetic mycoantigens. Russ. J. Bioorg. Chem. 44, 80–89. 10.1134/S1068162017060073

[B52] Krylov V. B. Solovev A. S. Argunov D. A. Latgé J. P. Nifantiev N. E. (2019). Reinvestigation of carbohydrate specificity of EB-A2 monoclonal antibody used in the immune detection of *Aspergillus fumigatus* galactomannan. Heliyon 5:e01173. 10.1016/j.heliyon.2019.e01173 30766929 PMC6360342

[B53] Latgé J. P. (2010). Tasting the fungal cell wall. Cell. Microbiol. 12, 863–872. 10.1111/j.1462-5822.2010.01474.x 20482553

[B54] Lay L. Manzoni L. Schmidt R. R. (1998). Synthesis of *N*-acetylglucosamine containing Lewis A and Lewis X building blocks based on *N*-tetrachlorophthaloyl protection — synthesis of Lewis X pentasaccharide. Carbohydr. Res. 310, 157–171. 10.1016/S0008-6215(98)00148-7 9809410

[B55] Li Z. Gildersleeve J. C. (2006). Mechanistic studies and methods to prevent aglycon transfer of thioglycosides. J. Am. Chem. Soc. 128, 11612–11619. 10.1021/ja063247q 16939286

[B56] Li Z. Gildersleeve J. C. (2007). An armed–disarmed approach for blocking aglycon transfer of thioglycosides. Tetrahedron Lett. 48, 559–562. 10.1016/j.tetlet.2006.11.126 19043616 PMC2587334

[B57] Ma L. Shen C. A. Gao L. Li D. W. Shang Y. R. Yin K.. (2016). Anti-inflammatory activity of chitosan nanoparticles carrying NF-κB/p65 antisense oligonucleotide in RAW264.7 macropghage stimulated by lipopolysaccharide. Colloids Surf. B 142, 297–306. 10.1016/j.colsurfb.2016.02.031 26970817

[B58] Matveev A. L. Krylov V. B. Emelyanova L. A. Solovev A. S. Khlusevich Y. A Baykov I. K.. (2018). Novel mouse monoclonal antibodies specifically recognize *Aspergillus fumigatus* galactomannan. PLoS ONE 13:e0193938. 10.1371/journal.pone.0193938 29518144 PMC5843280

[B59] Matveev A. L. Krylov V. B. Khlusevich Y. A. Baykov I. K. Yashunsky D. V Emelyanova L. A.. (2019). Novelmouse monoclonal antibodies specifically recognizing β-(1→3)-D-glucan antigen. PLoS ONE 14:e0215535. 10.1371/journal.pone.0215535 31022215 PMC6483564

[B60] Moran H. B. T. Turley J. L. Andersson M. Lavelle E. C. (2018). Immunomodulatory properties of chitosan polymers. Biomaterials 184, 1–9. 10.1016/j.biomaterials.2018.08.054 30195140

[B61] Mori A. Oleszycka E. Sharp F. A. Coleman M. Ozasa Y. Singh M.. (2012). The vaccine adjuvant alum inhibits IL-12 by promoting PI3 kinase signaling while chitosan does not inhibit IL-12 and enhances Th1 and Th17 responses. Eur. J. Immunol. 42, 2709–2719. 10.1002/eji.201242372 22777876

[B62] Panos I. Acosta N. Heras A. (2008). New drug delivery systems based on chitosan. Curr. Drug Discovery Technol. 5, 333–341. 10.2174/157016308786733528 19075614

[B63] Paulovičová E. Paulovičová L. Hrubiško M. Krylov V. B. Argunov D. A. Nifantiev N. E. (2017). Immunobiological activity of synthetically prepared immunodominant galactomannosides structurally mimicking Aspergillus galactomannan. Front. Immunol. 8:1273. 10.3389/fimmu.2017.01273 29081774 PMC5645502

[B64] Paulovičová E. Paulovičová L. Pilišiová R. Jančinová V. Yashunsky D. V. Karelin A. A.. (2016). The evaluation of β-(1→3)-nonaglucoside as an anti-Candida albicans immune response inducer. Cell. microbiol. 18, 1294–1307. 10.1111/cmi.12631 27310441

[B65] Paulovičová L. Paulovičová E. Bystrický S. (2014). Immunological basis of anti-Candida vaccines focused on synthetically prepared cell wall mannan-derived manno-oligomers. Microbiol. Immunol. 58, 545–551. 10.1111/1348-0421.12195 25154867

[B66] Paulovičová L. Paulovičová E. Karelin A. A. Tsvetkov Y. E. Nifantiev N. E. Bystrický S. (2013). Effect of branched α-oligomannoside structures on induction of anti-candida humoral immune response. Scand. J. Immunol. 77, 431–441. 10.1111/sji.12044 23488735

[B67] Paulovičová L. Paulovičová E. Karelin A. A. Tsvetkov Y. E. Nifantiev N. E. Bystrický S. (2015). Immune cell response to Candida cell wall mannan derived branched α-oligomannoside conjugates in mice. J. Microbiol. Immunol. Infect. 48, 9–19. 10.1016/j.jmii.2013.08.020 24239417

[B68] Rahmani S. Hakimi S. Esmaeily A. Samadi F. Y. Mortazavian E. Nazari M.. (2019). Novel chitosan based nanoparticles as gene delivery systems to cancerous and noncancerous cells. Int. J. Pharm. 560, 306–314. 10.1016/j.ijpharm.2019.02.016 30797073

[B69] Schubert M. Xue S. Ebel F. Vaggelas A. Krylov V. B. Nifantiev N. E.. (2019). Monoclonal antibody AP3 bindsgalactomannan antigens displayed by the pathogens *Aspergillus flavus, A. fumigatus*, and *A. parasiticus*. Front. Cell. Infect. Microbiol. 9:234. 10.3389/fcimb.2019.00234 31380292 PMC6646516

[B70] Shibata Y. Metzger W. J. Myrvik Q. N. (1997). Chitin particle-induced cell-mediated immunity is inhibited by soluble mannan: mannose receptor-mediated phagocytosis initiates IL-12 production. J. Immunol. 159, 2462–2467. 9278339

[B71] Smith S. G. Koppolu B. P. Ravindranathan S. Kurtz S. L. Yang L. Katz M. D.. (2015). Intravesical chitosan/interleukin-12 immunotherapy induces tumor-specific systemic immunity against murine bladder cancer. Cancer Immunol. Immunother. 64, 689–696. 10.1007/s00262-015-1672-x 25754122 PMC4458215

[B72] Tanaka H. Hamaya Y. Kotsuki H. (2017). A direct method for β-selective glycosylation with an N-acetylglucosamine donor armed by a 4-O-TBDMS protecting group. Molecules 22:429. 10.3390/molecules22030429 28282905 PMC6155425

[B73] Tangsadthakun C. Kanokpanont S. Sanchavanakit N. Pichyangkura R. Banaprasert T. Tabata Y.. (2007). The influence of molecular weight of chitosan on the physical and biological properties of collagen/chitosan scaffolds. J. Biomater. Sci., Polym. Ed. 18, 147–163. 10.1163/156856207779116694 17323850

[B74] Tavaria F. K. Costa E. M. Gens E. J. Malcata F. X. Pintado M. E. (2013). Influence of abiotic factors on the antimicrobial activity of chitosan. J. Dermatol. 40, 1014–1019. 10.1111/1346-8138.12315 24330167

[B75] Tsai G. J. Zhang S. L. Shieh P. L. (2004). Antimicrobial activity of a low-molecular-weight chitosan obtained from cellulase digestion of chitosan. J. Food Prot. 67, 396–398. 10.4315/0362-028X-67.2.396 14968977

[B76] Tsvetkov Y. E. Burg-Roderfeld M. Loers G. Ardá A. Sukhova E. V. Khatuntseva E. A.. (2012). Synthesis and molecular recognition studies of the HNK-1 trisaccharide and related oligosaccharides. The specificity of monoclonal anti-HNK-1 antibodies as assessed by surface plasmon resonance and STD NMR. J. Am. Chem. Soc. 134, 426–435. 10.1021/ja2083015 22087768

[B77] Turner M. D. Nedjai B. Hurst T. Pennington D. J. (2014). Cytokines and chemokines: At the crossroads of cell signalling and inflammatory disease. Biochim. Biophys. Acta Mol. Cell Res. 1843, 2563–2582. 10.1016/j.bbamcr.2014.05.014 24892271

[B78] Upadhya R. Baker L. G. Lam W. C. Specht C. A. Donlin M. J. Lodge J. K. (2018). *Cryptococcus neoformans* Cda1 and its chitin deacetylase activity are required for fungal pathogenesis. mBio 9, e02087–e02018. 10.1128/mBio.02087-18 30459196 PMC6247093

[B79] Wagener J. Malireddi R. K. Lenardon M. D. Koberle M. Vautier S. MacCallum D. M.. (2014). Fungal chitin dampens inflammation through IL-10 induction mediated by NOD2 and TLR9 activation. PLoS Pathogens 10:e1004050. 10.1371/journal.ppat.1004050 24722226 PMC3983064

[B80] Wong S. S. W. Krylov V. B. Argunov D. A. Karelin A. A. Bouchara J. P. Fontaine T.. (2020). Potential of chemicallysynthesized oligosaccharides to precisely define the carbohydrate moietiesof the fungal cell wall responsible for the human immune response. Theexample of the Aspergillus fumigatus cell wall galactomannan. mSphere e5:e00688–e00619. 10.1128/mSphere.00688-19 31915215 PMC6952192

[B81] Wu J. Tang C. Yin C. (2017). Co-delivery of doxorubicin and interleukin-2 via chitosan based nanoparticles for enhanced antitumor efficacy. Acta Biomater. 47, 81–90. 10.1016/j.actbio.2016.10.012 27729232

[B82] Xia Y. Fan Q. Hao D. Wu J. Ma G. Su Z. (2015). Chitosan-based mucosal adjuvants: Sunrise on the ocean. Vaccine 33, 5997–6010. 10.1016/j.vaccine.2015.07.101 26271831 PMC7185844

[B83] Xin H. Dziadek S. Bundle D. R. Cutler J. E. (2008). Synthetic glycopeptide vaccines combining β-mannan and peptide epitopes induce protection against candidiasis. Proc. Natl. Acad. Sci. U.S.A. 105, 13526–13531. 10.1073/pnas.0803195105 18725625 PMC2533223

[B84] Yang E. J. Kim J. G. Kim J. Y. Kim S. Lee N. Hyun C. G. (2010). Anti-inflammatory effect of chitosan oligosaccharides in RAW 264.7 cells. Cent. Eur. J. Biol. 5, 95–102. 10.2478/s11535-009-0066-5

[B85] Yang Y. Xing R. Liu S. Qin Y. Li K. Yu H.. (2019). Immunostimulatory Effects of Chitooligosaccharides on RAW 264.7 Mouse Macrophages via Regulation of the MAPK and PI3K/Akt Signaling Pathways. Marine Drugs 17:36. 10.3390/md17010036 30626153 PMC6357175

[B86] Yoon H. J. Moon M. E. Park H. S. Im S. Y. Kim Y. H. (2007). Chitosan oligosaccharide (COS) inhibits LPS-induced inflammatory effects in RAW 264.7 macrophage cells. Biochem. Biophys. Res. Commun. 358, 954–959. 10.1016/j.bbrc.2007.05.042 17512902

[B87] Yudina O. N. Tsvetkov Y. E. Nifantiev N. E. (2015). Synthesis of 2-aminoethyl glycosides of chitooligosaccharides. Russ. Chem. Bull. 64, 2932–2941. 10.1007/s11172-015-1250-6

[B88] Yudina O. N. Tsvetkov Y. E. Nifantiev N. E. (2016). Conditions of catalytic hydrogenolysis for the simultaneous reduction of azido group and debenzylation of chitooligosaccharides. Synthesis of biotinylated derivatives of chitooligosaccharides. Russ. Chem. Bull. 65, 2937–2942. 10.1007/s11172-016-1681-8

[B89] Zhang P. Liu W. Peng Y. Han B. Yang Y. (2014). Toll like receptor 4 (TLR4) mediates the stimulating activities of chitosan oligosaccharide on macrophages. Int. Immunopharmacol. 23, 254–261. 10.1016/j.intimp.2014.09.007 25237008

