# Supplementary material for: Synthesis of Biotin-Tagged Chitosan Oligosaccharides and Assessment of Their Immunomodulatory Activity
Source: Front Chem. 2020 Dec 1;8:554732. doi: 10.3389/fchem.2020.554732 (PMC7736555; doi:10.3389/fchem.2020.554732)
Supplement: Supplementary file 1 [file Table_1.DOCX]

Supplementary Material

**^1^H, ^13^C NMR and mass spectra of synthesized compounds**


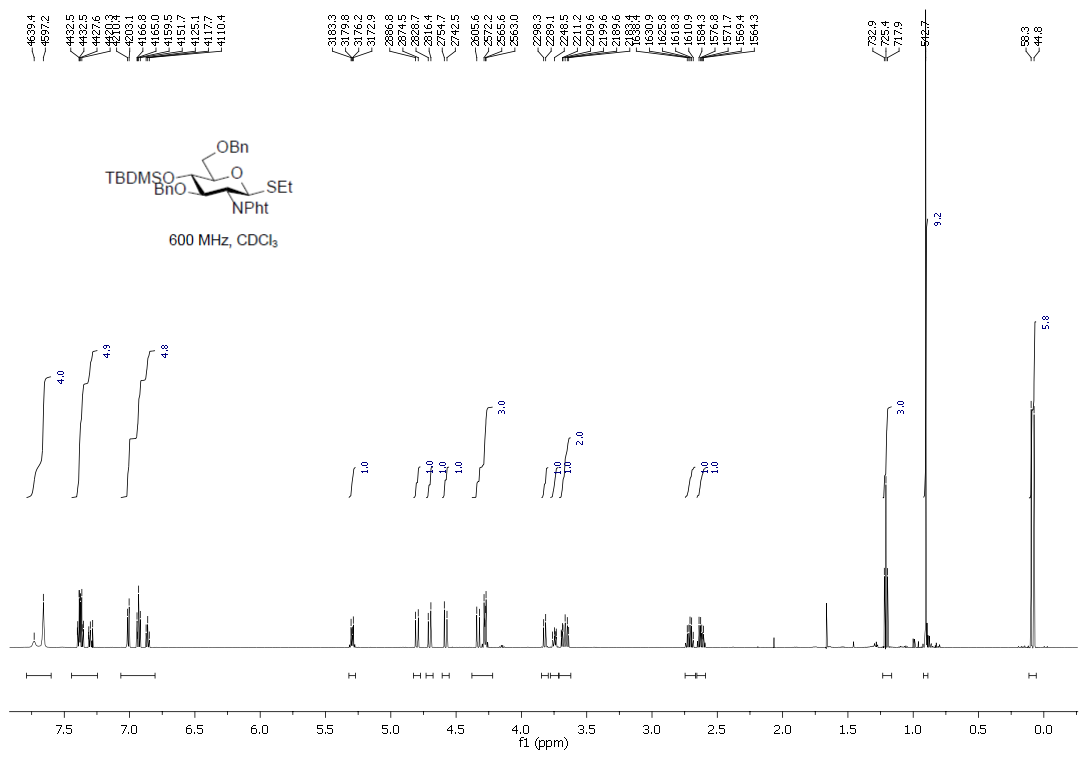


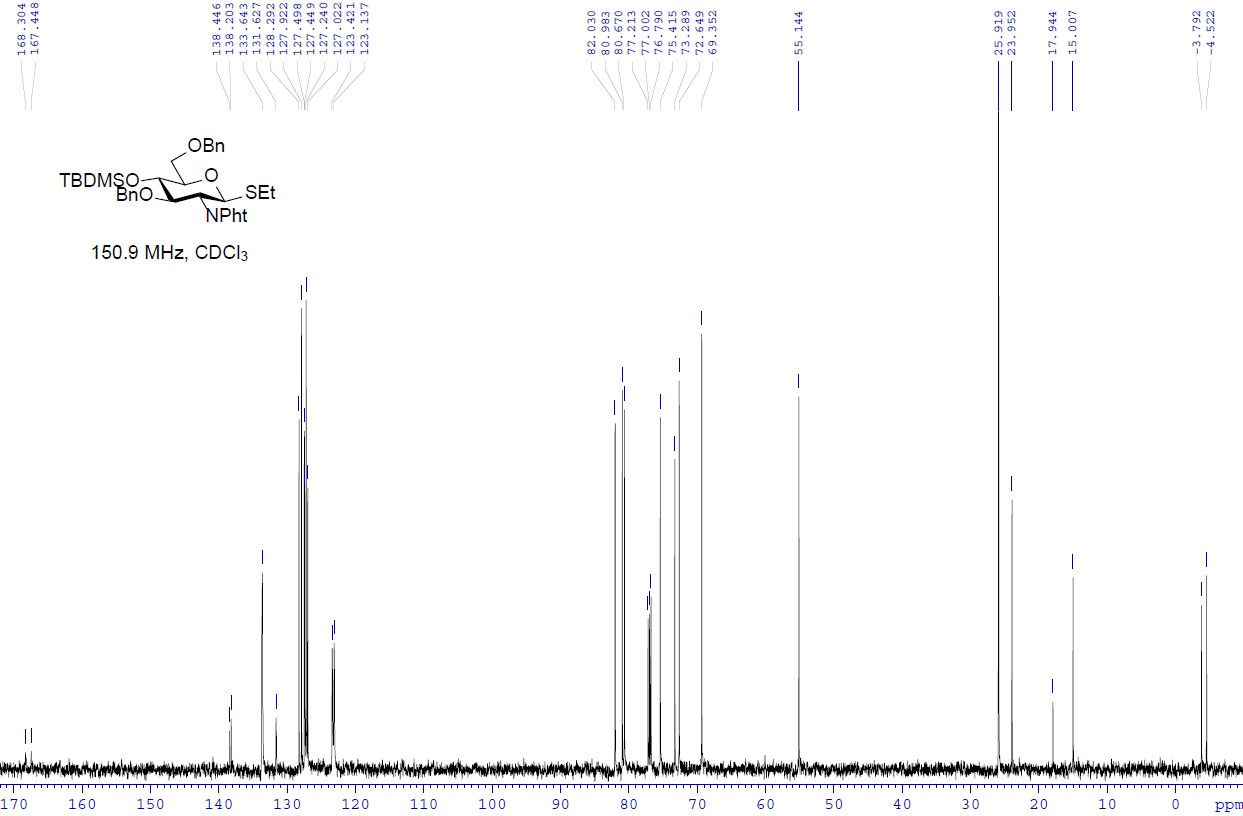


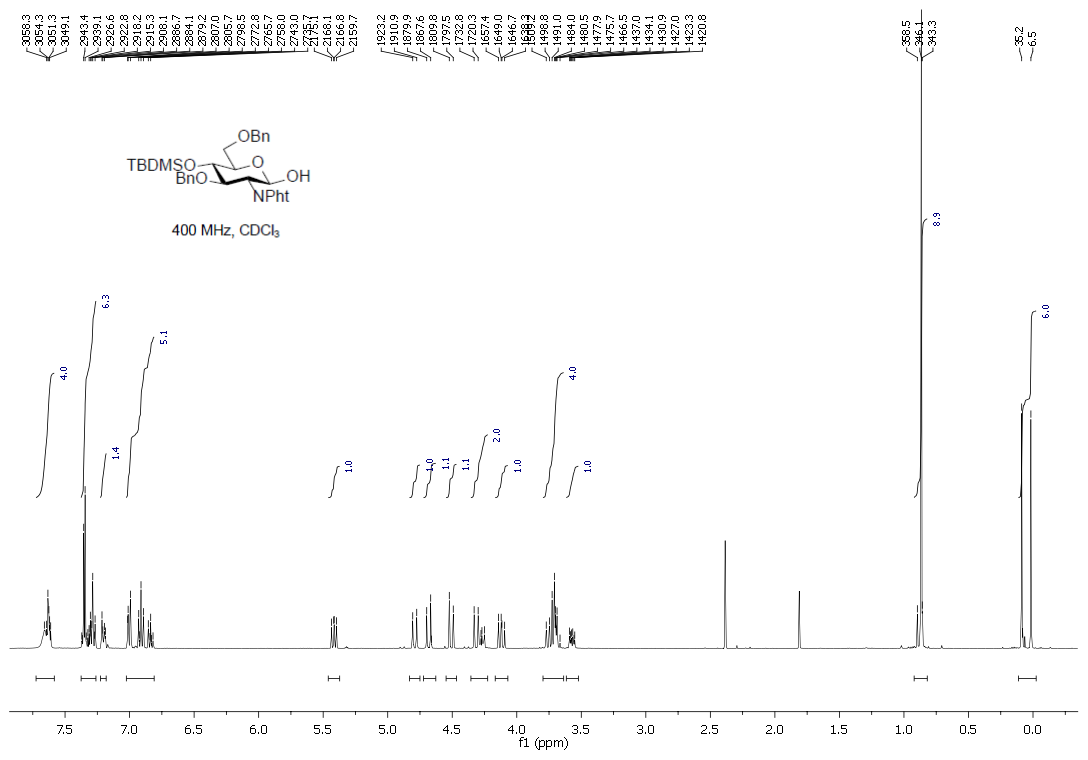


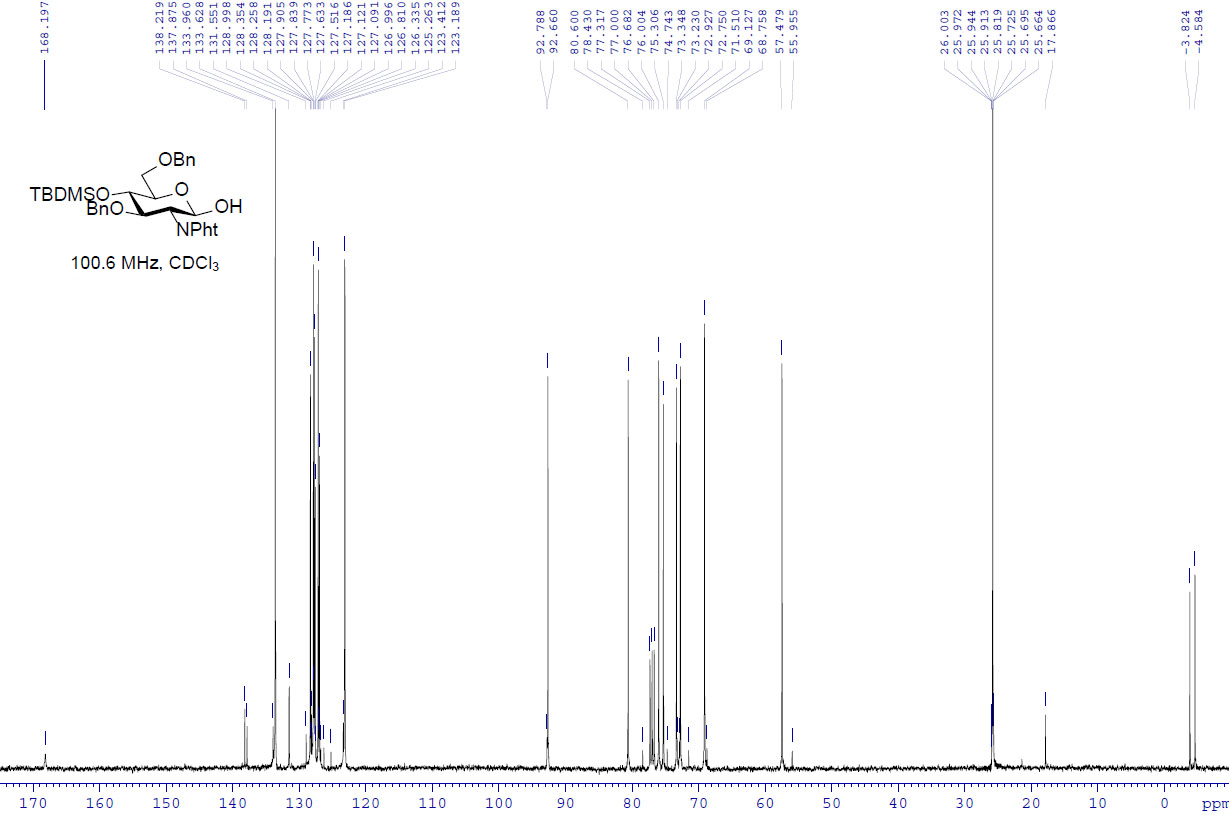


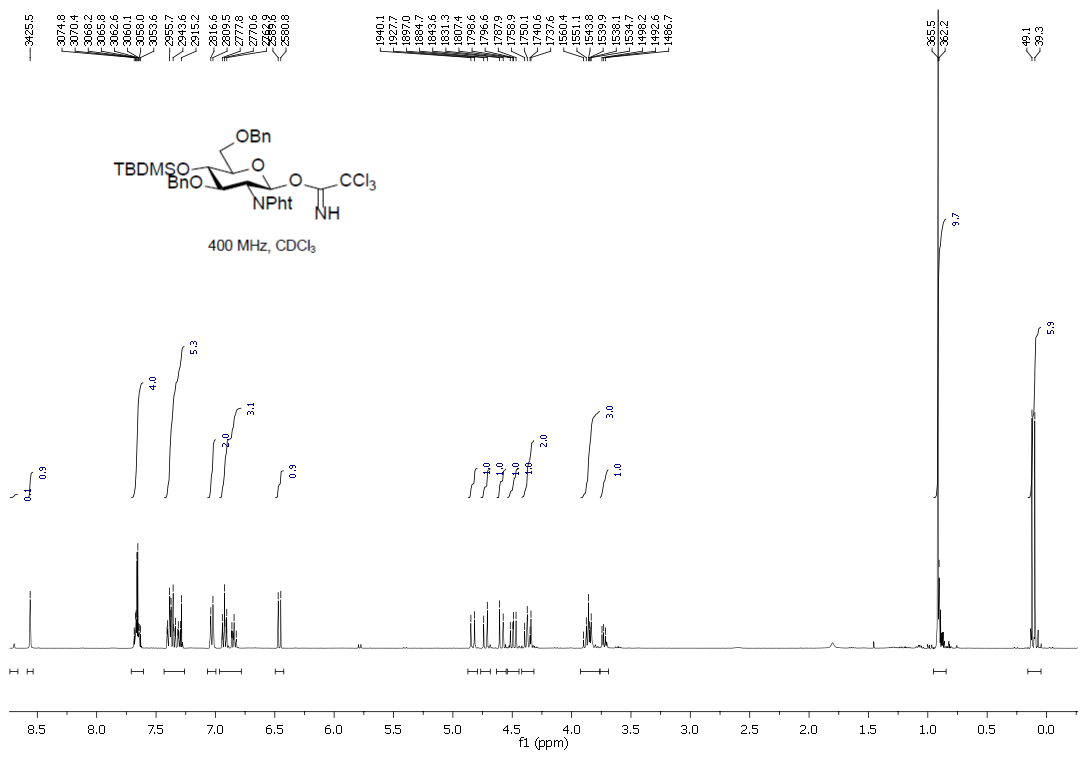


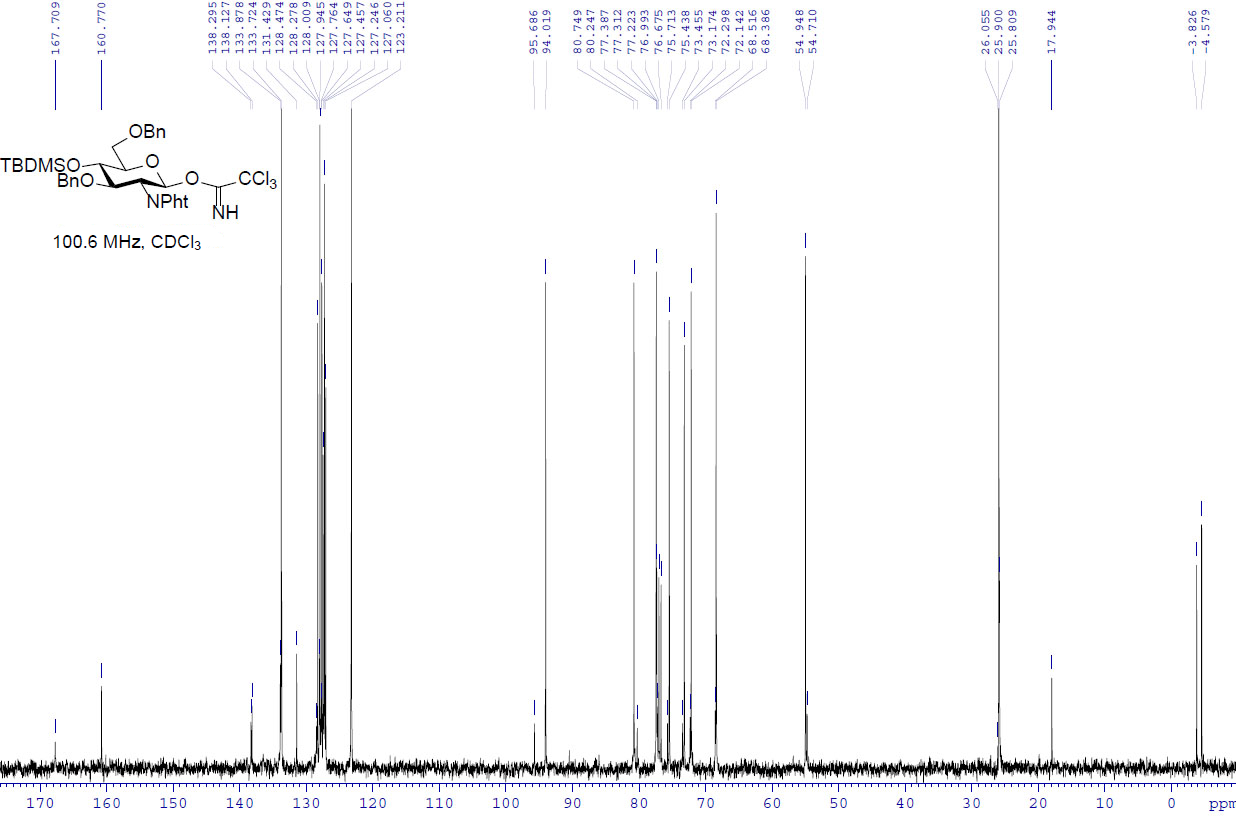


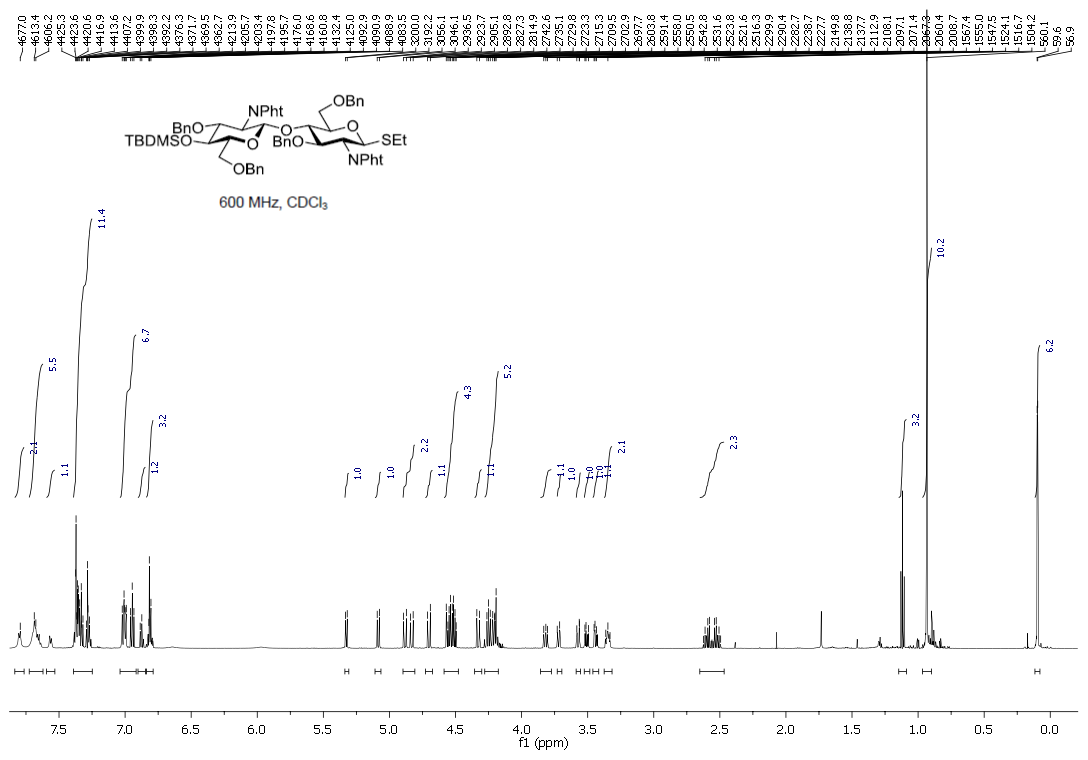


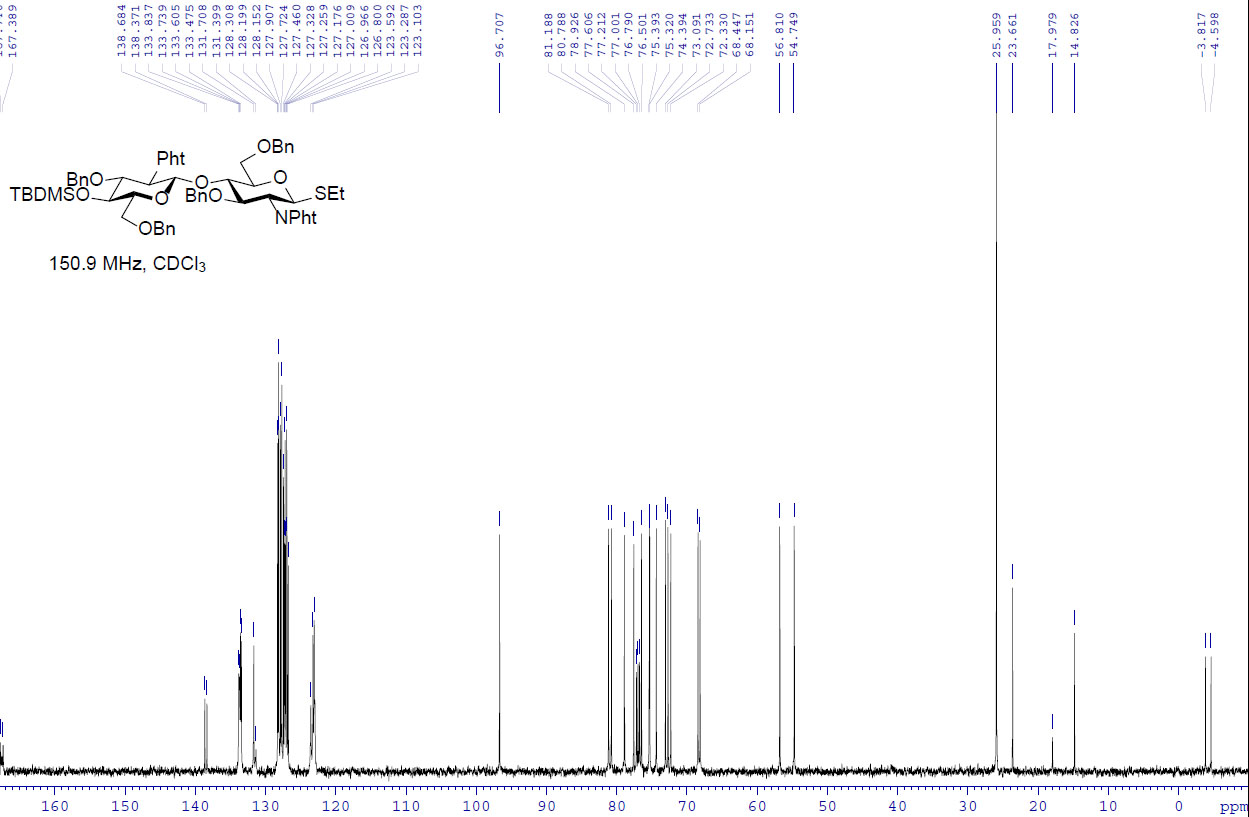


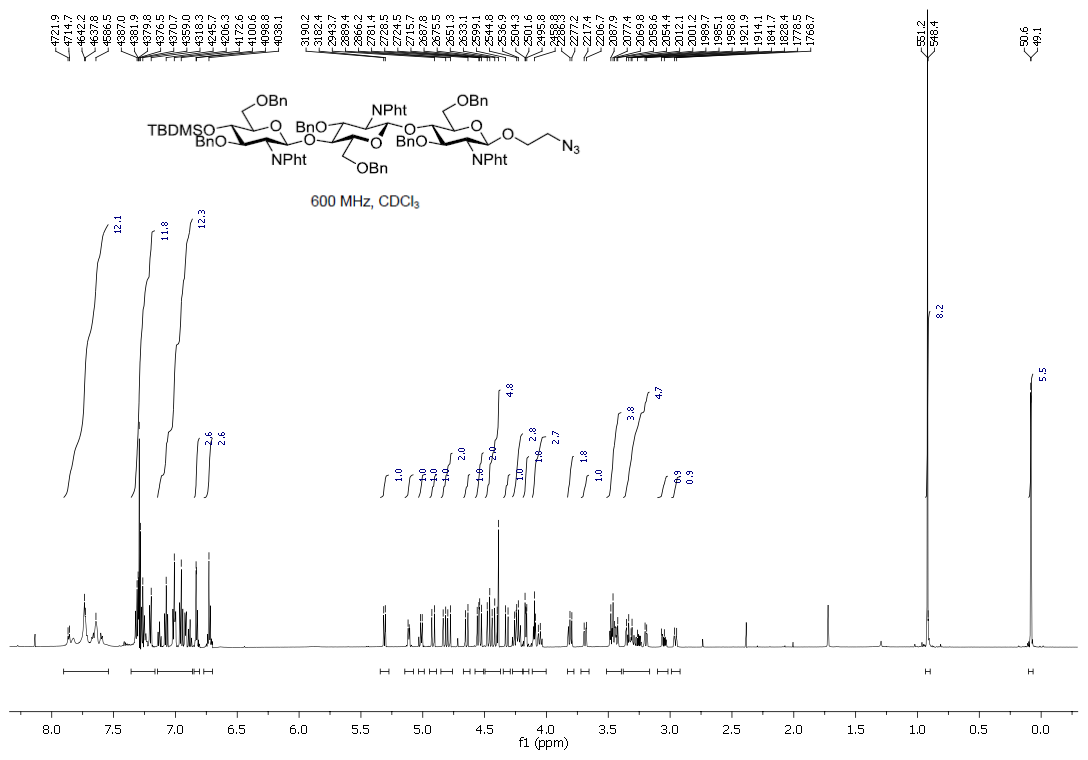


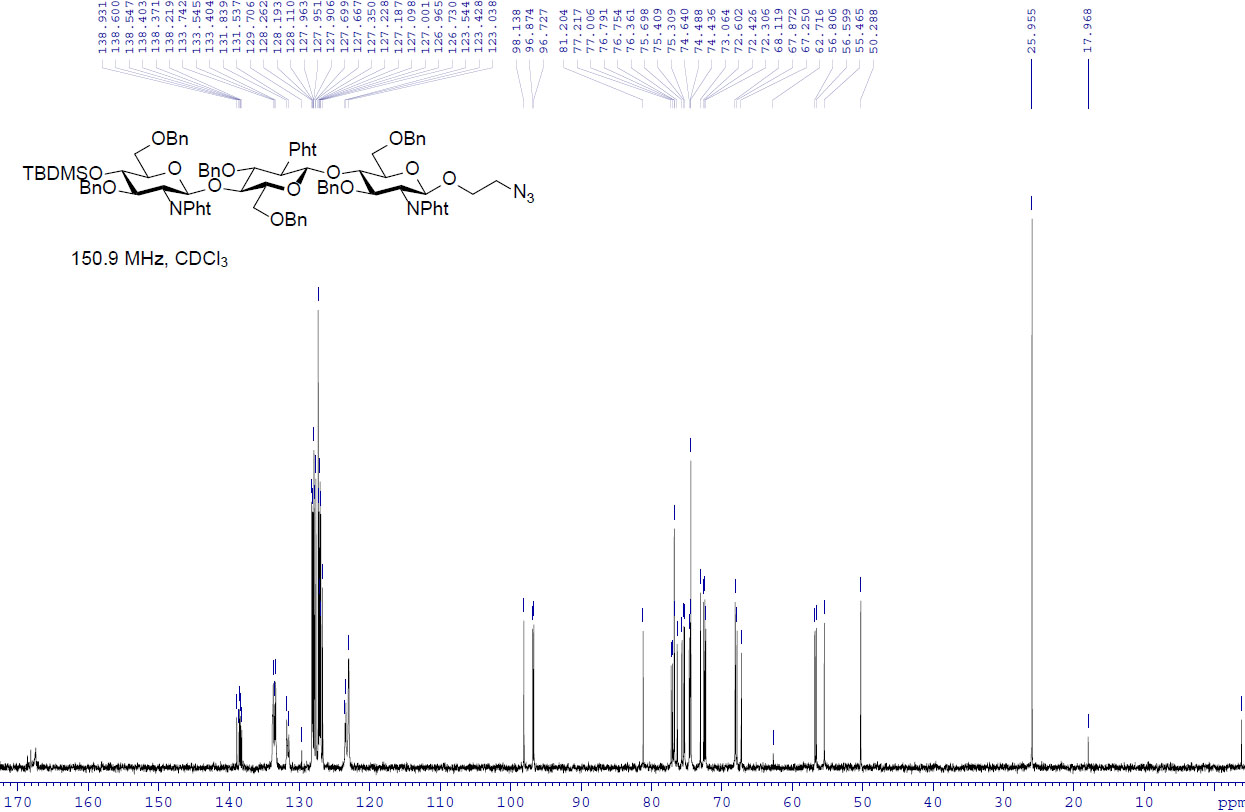


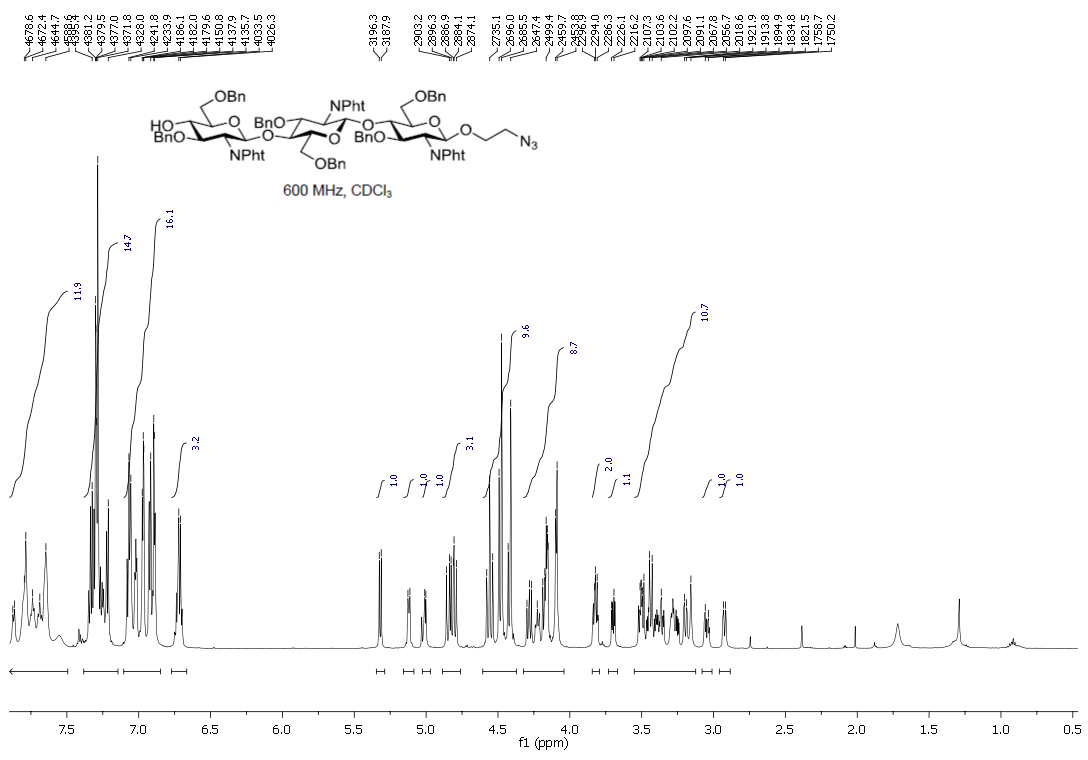


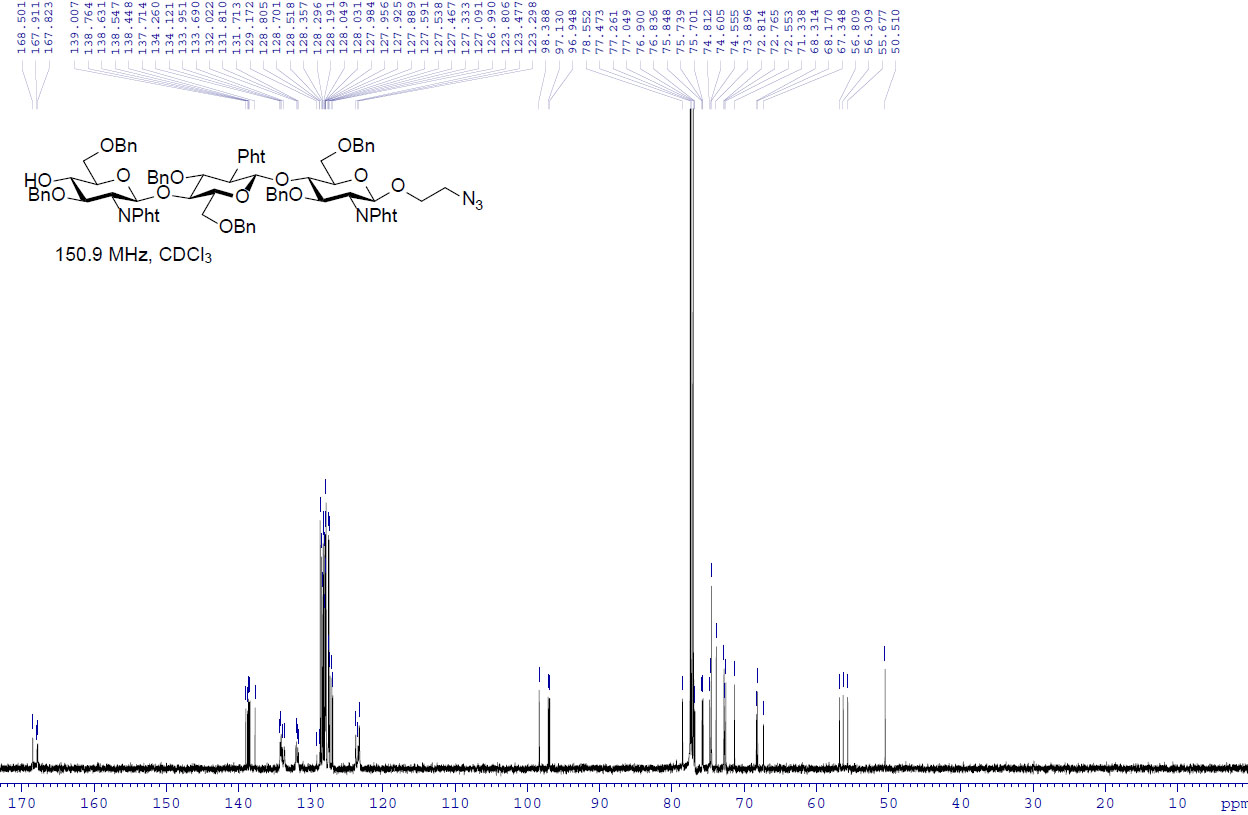


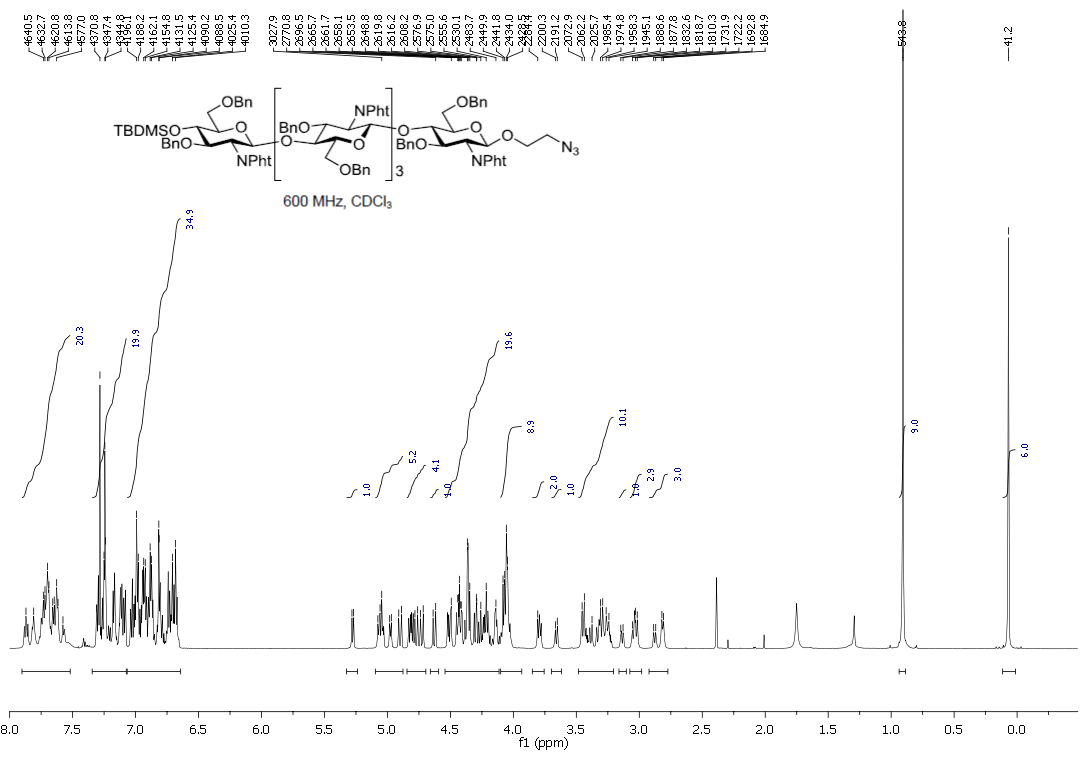


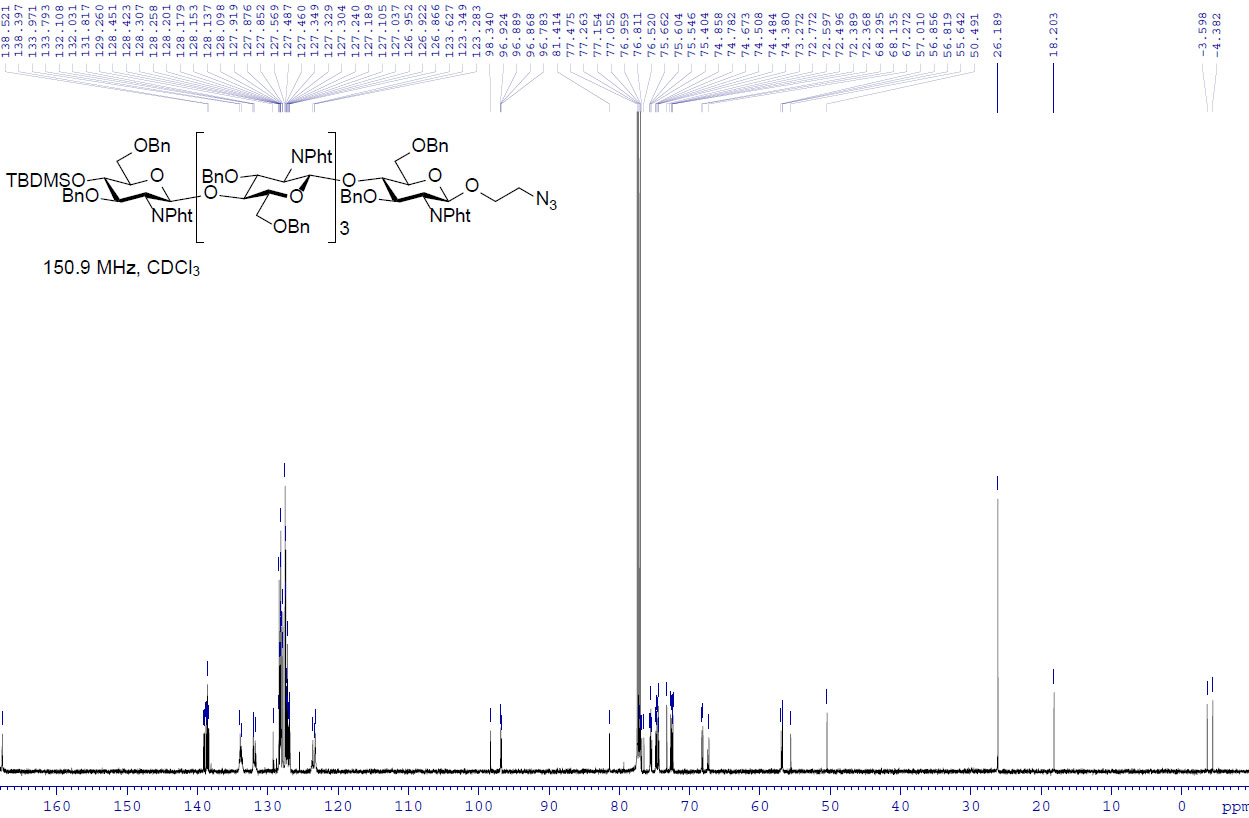


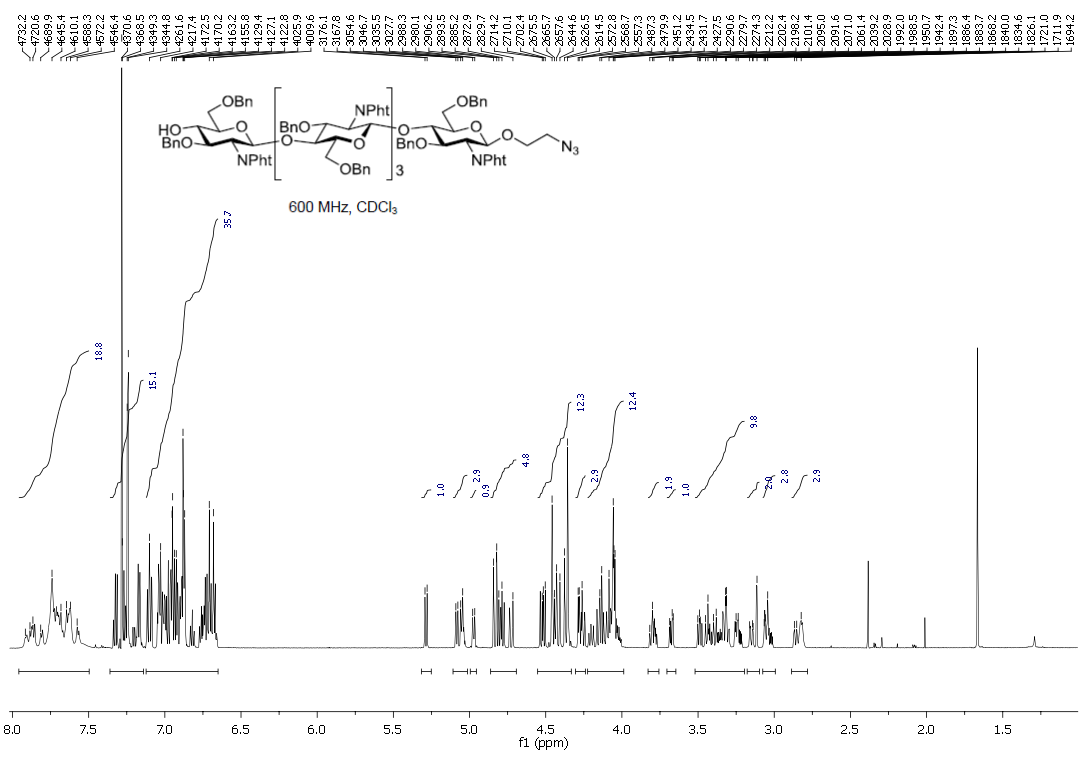


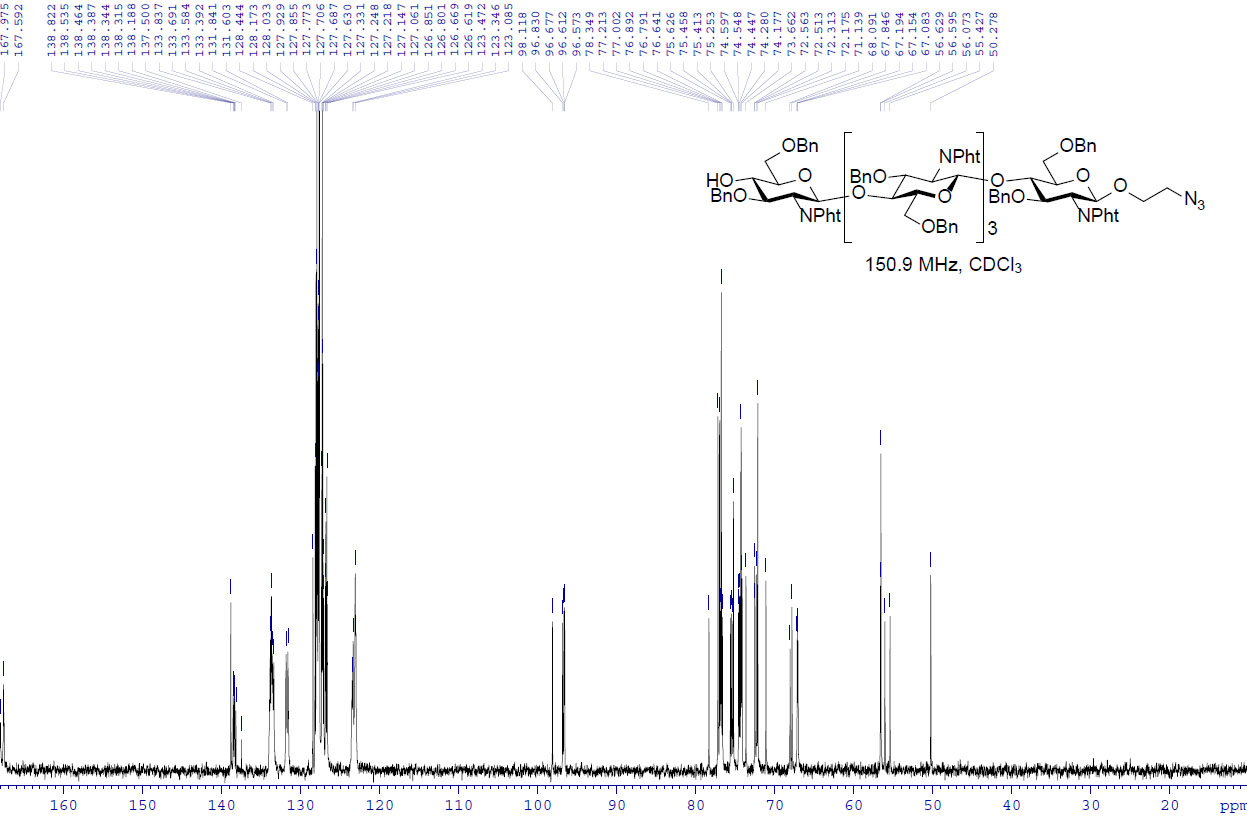


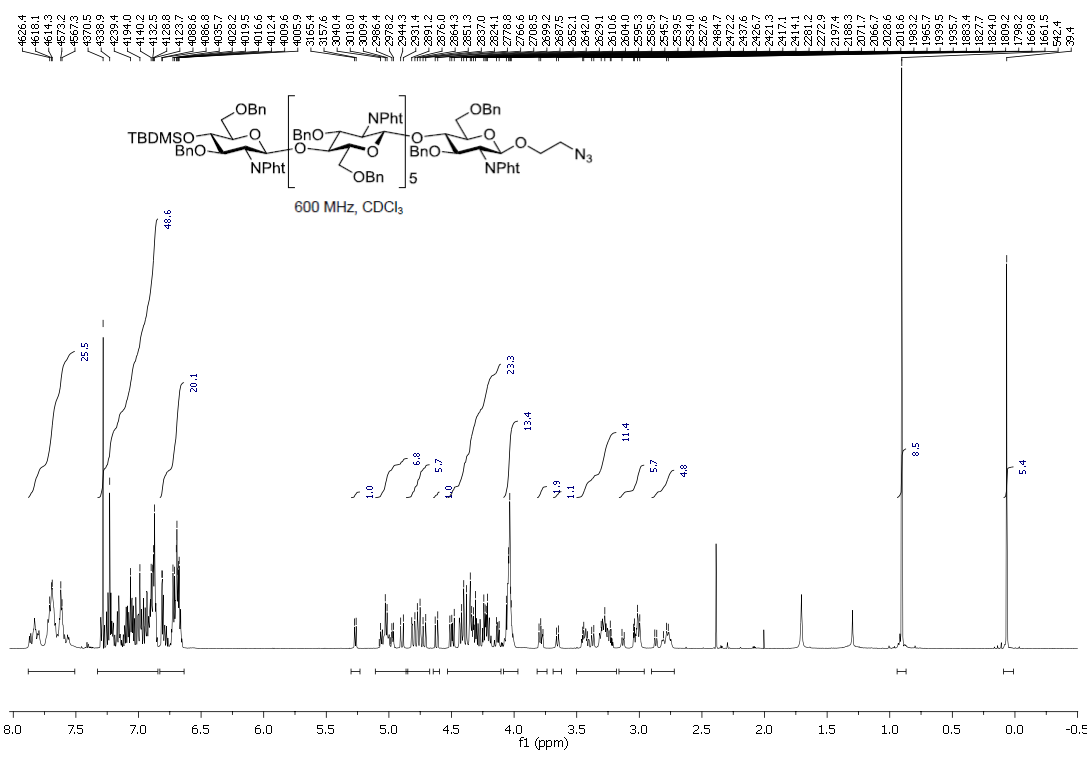


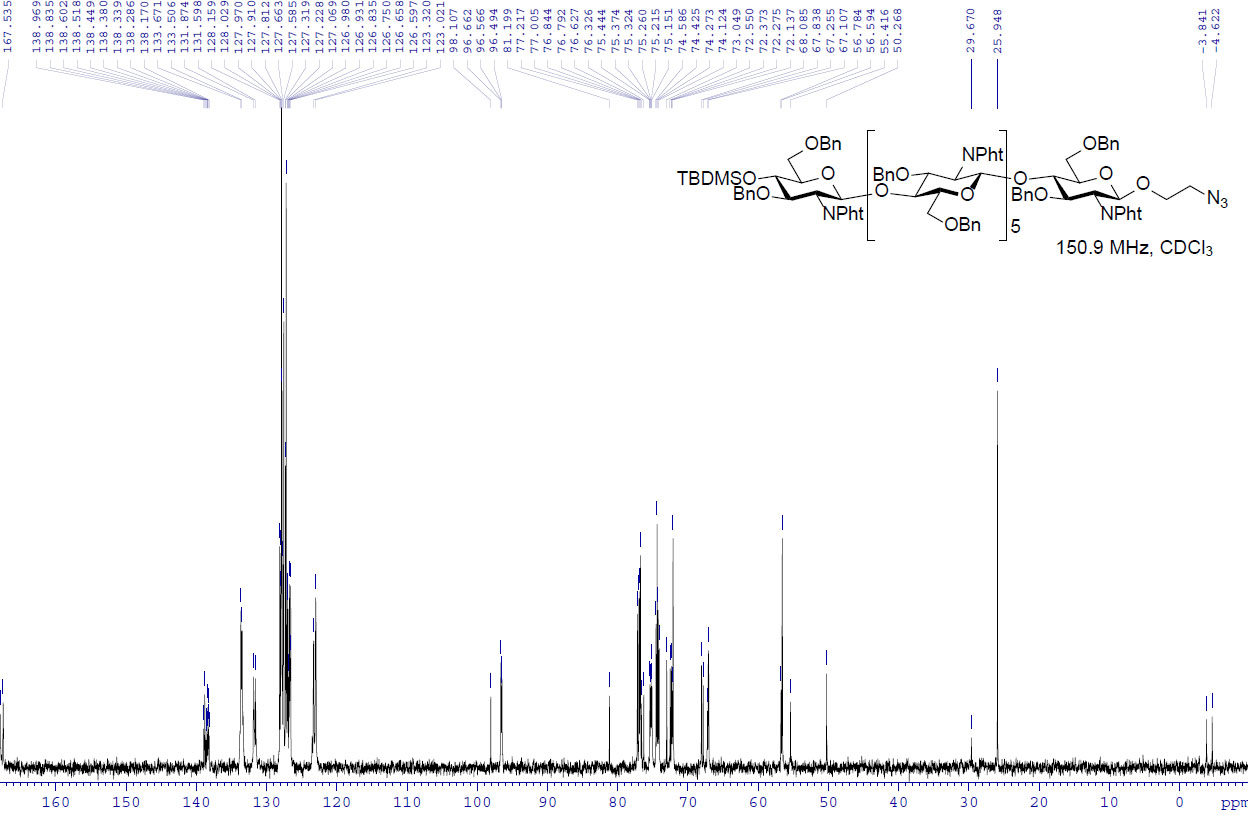


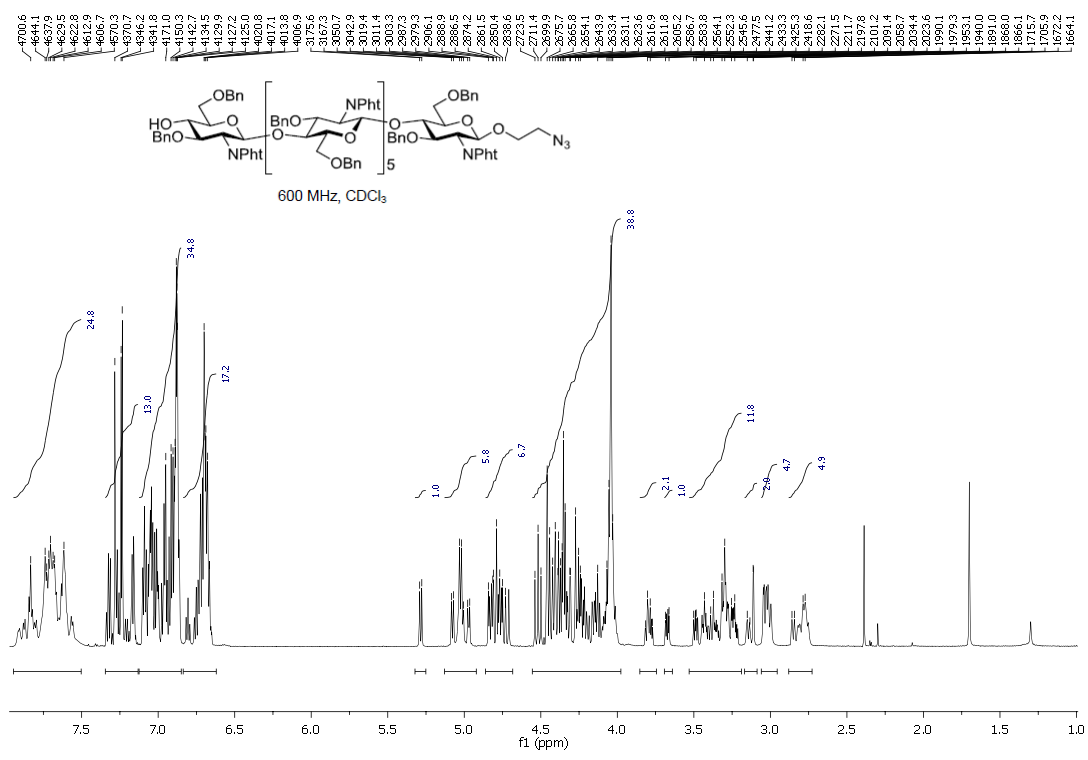


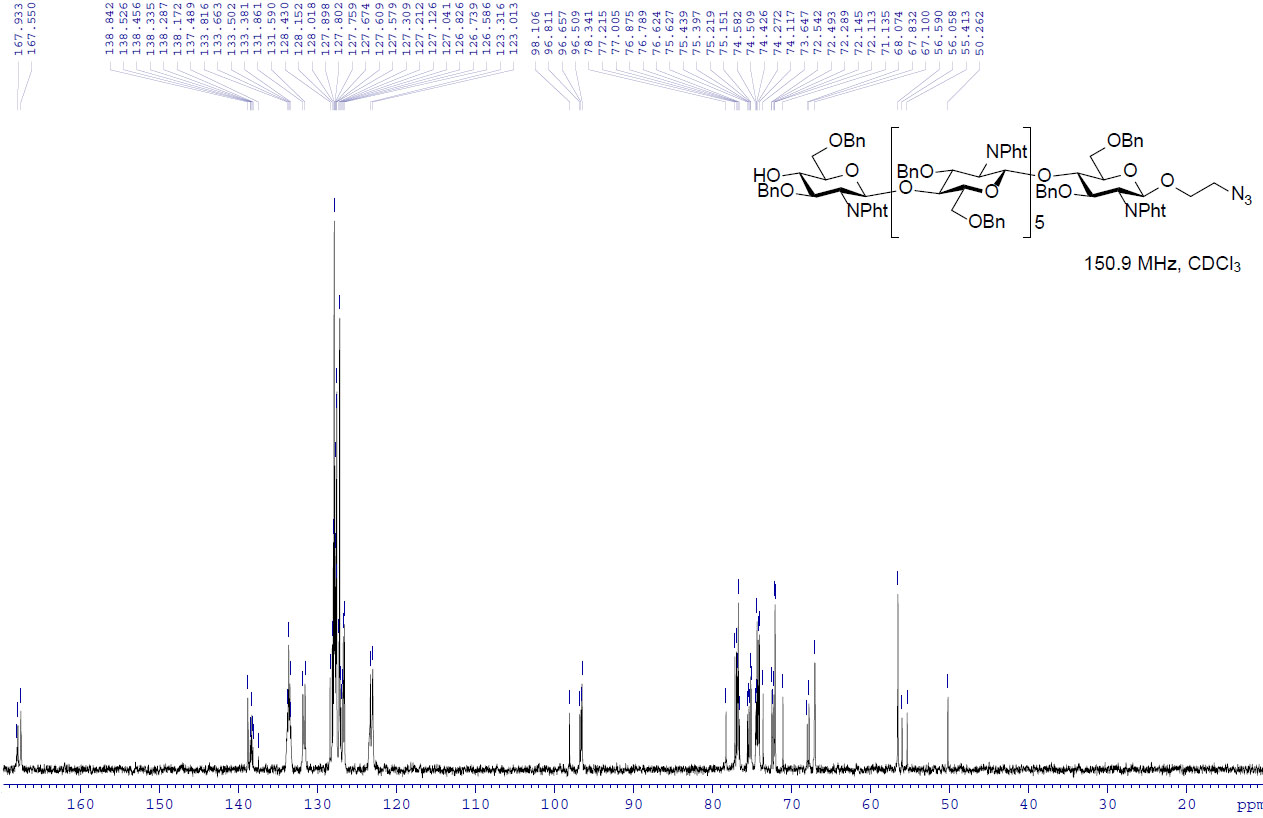


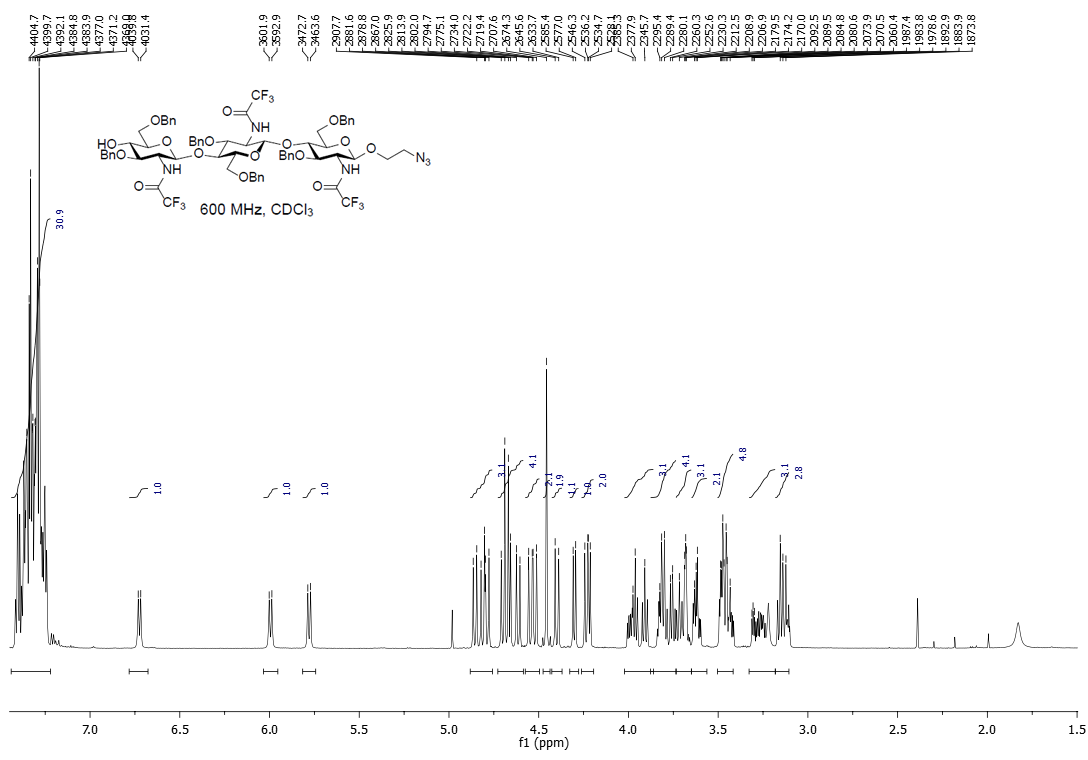


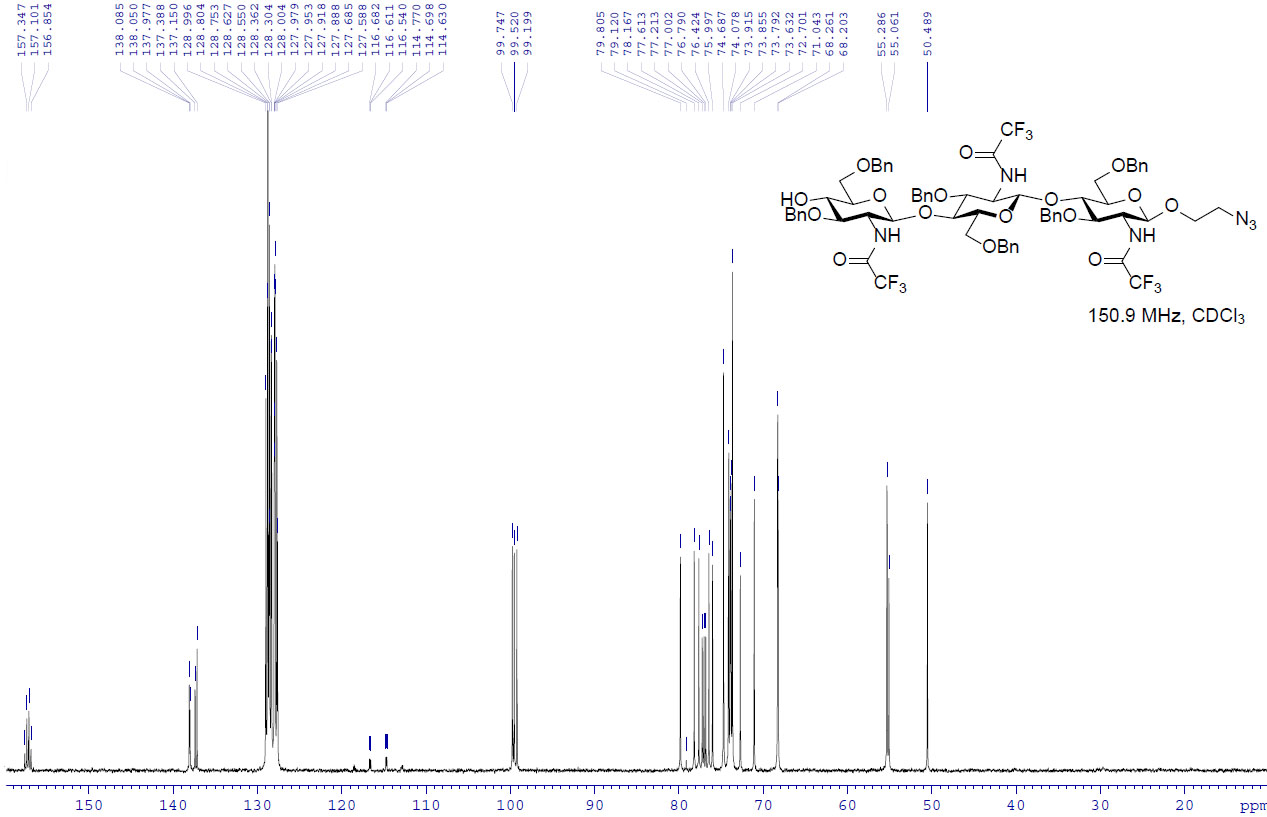


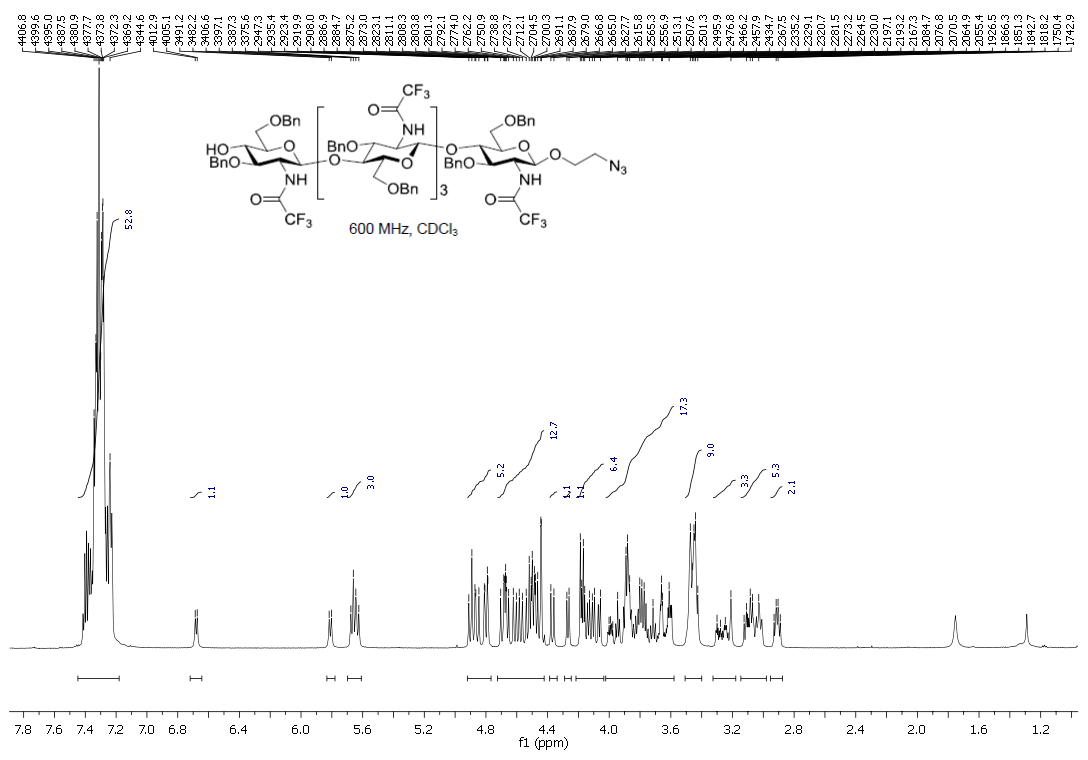


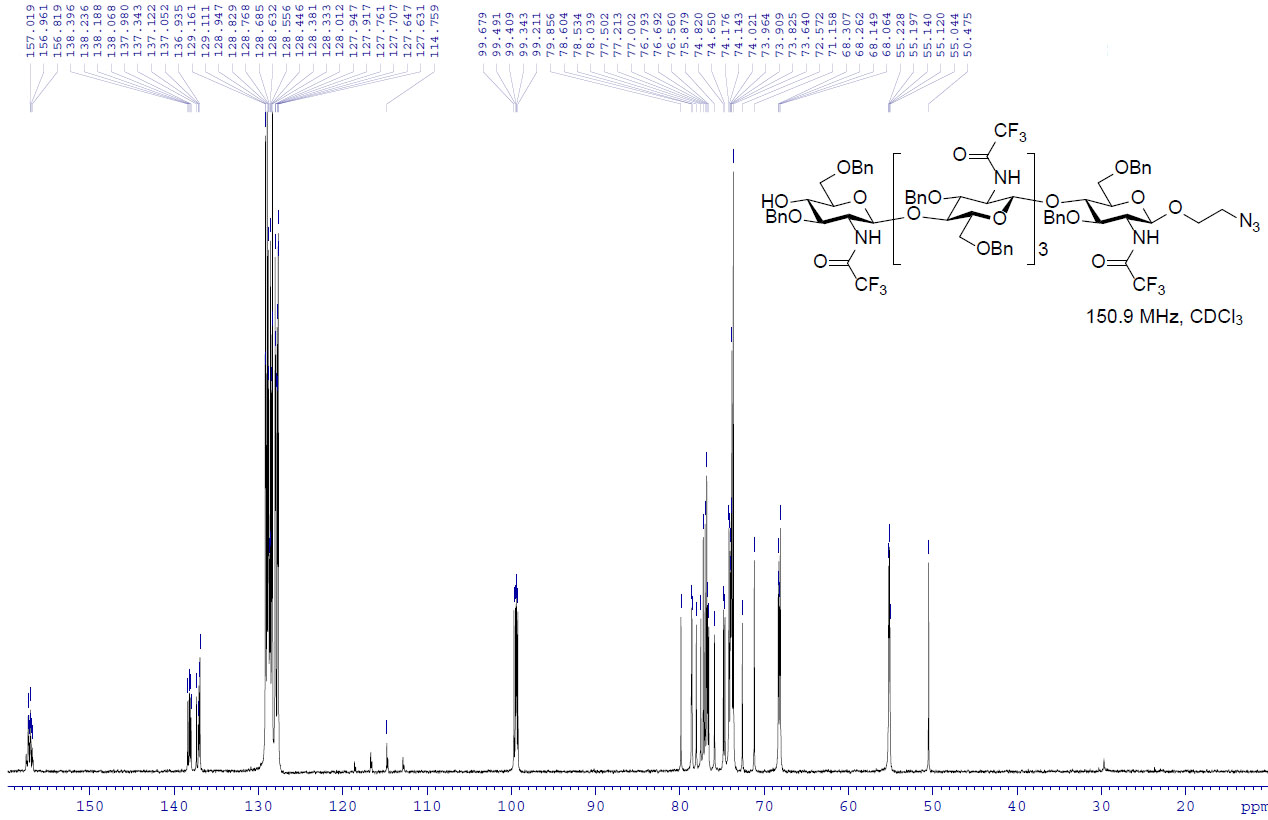


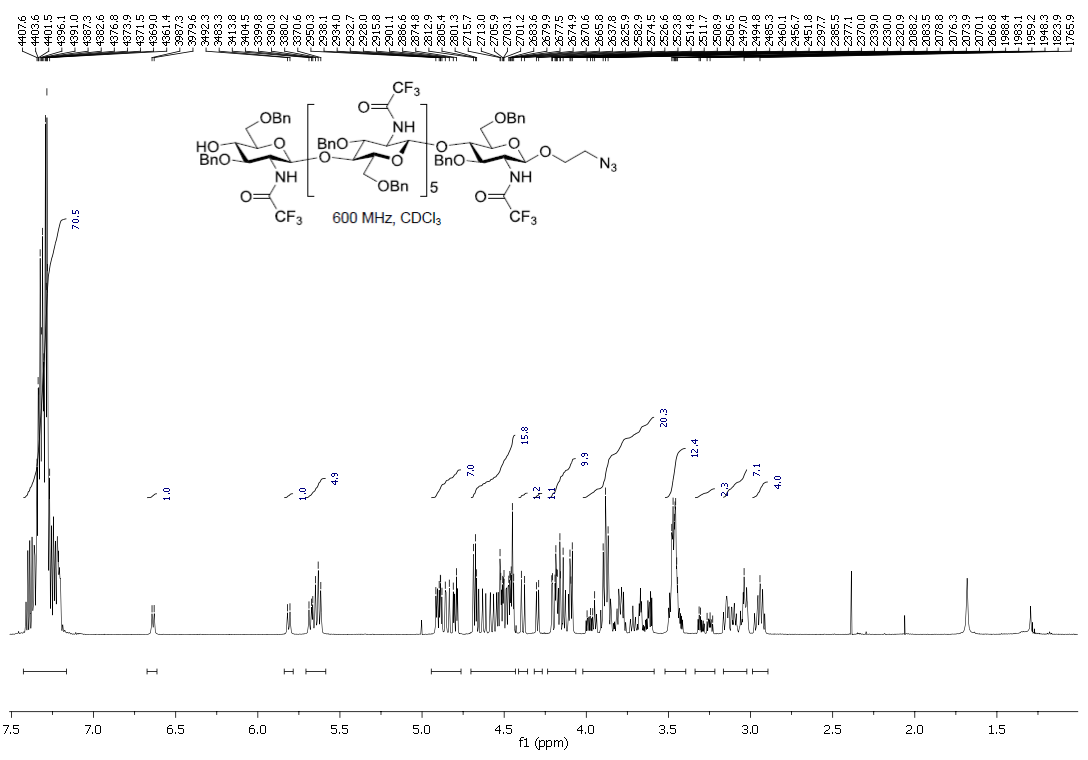


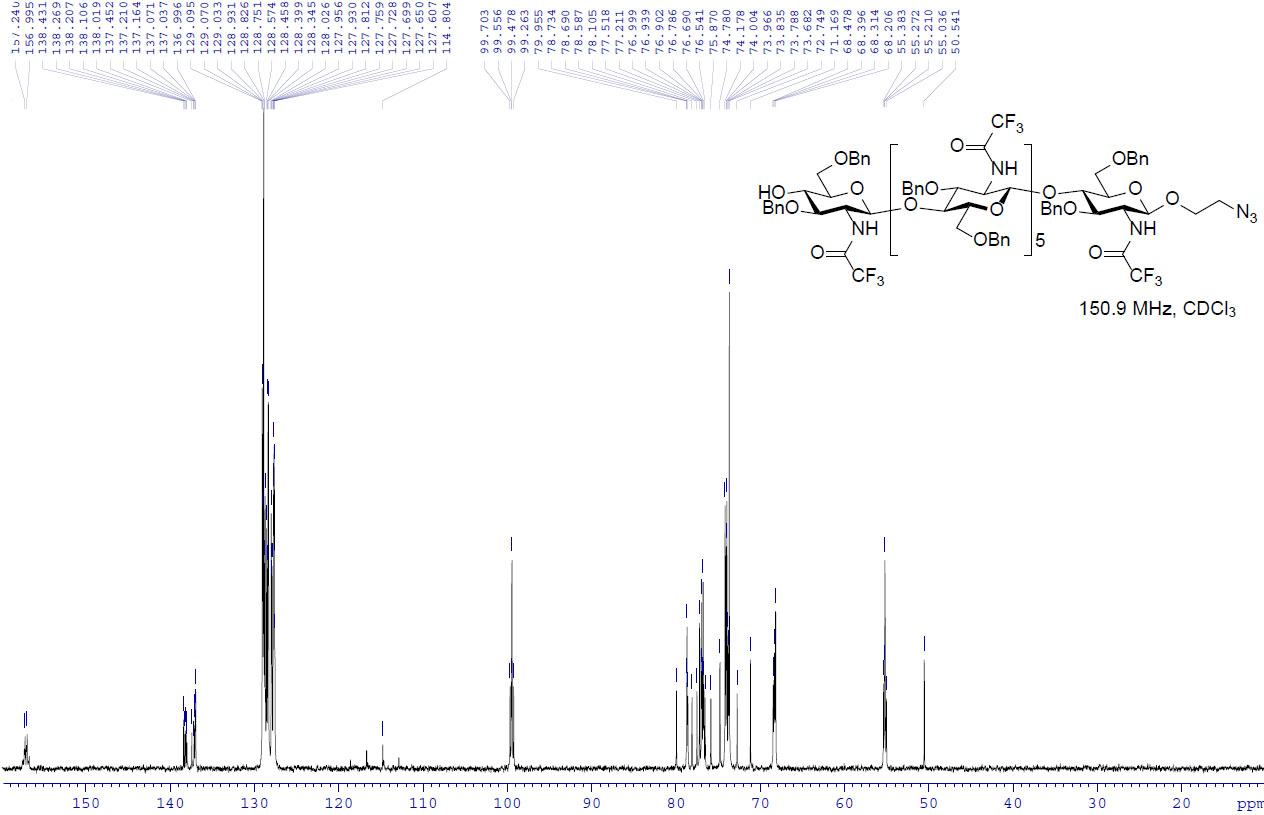


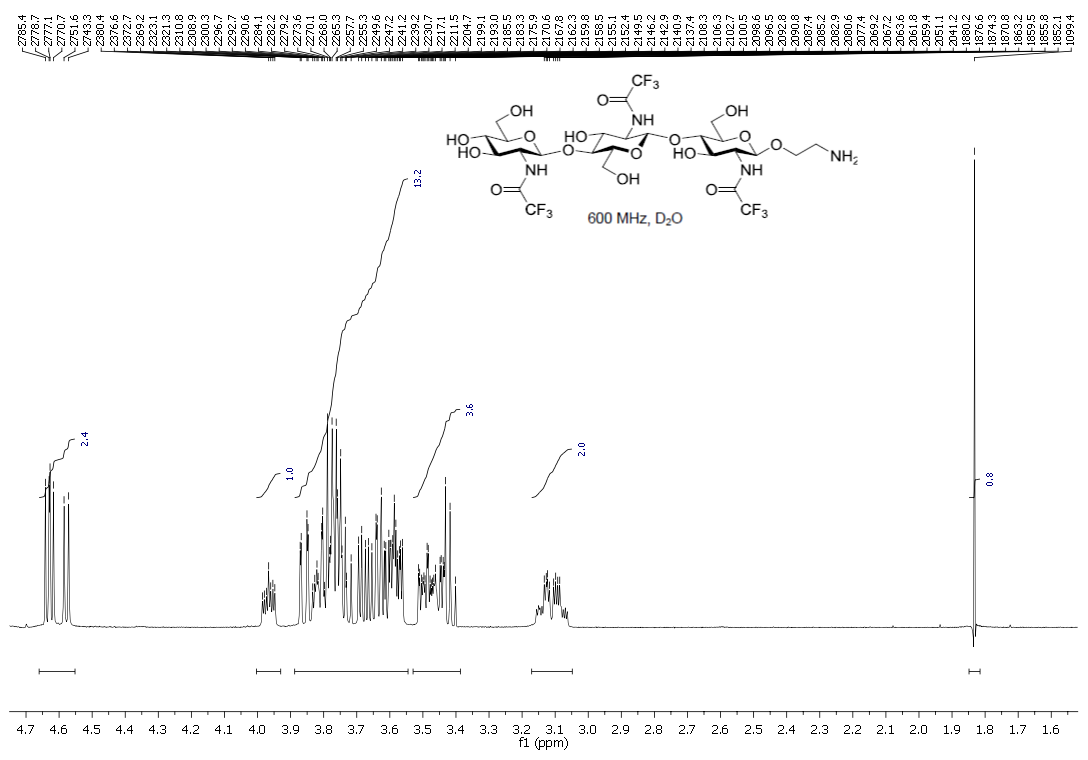


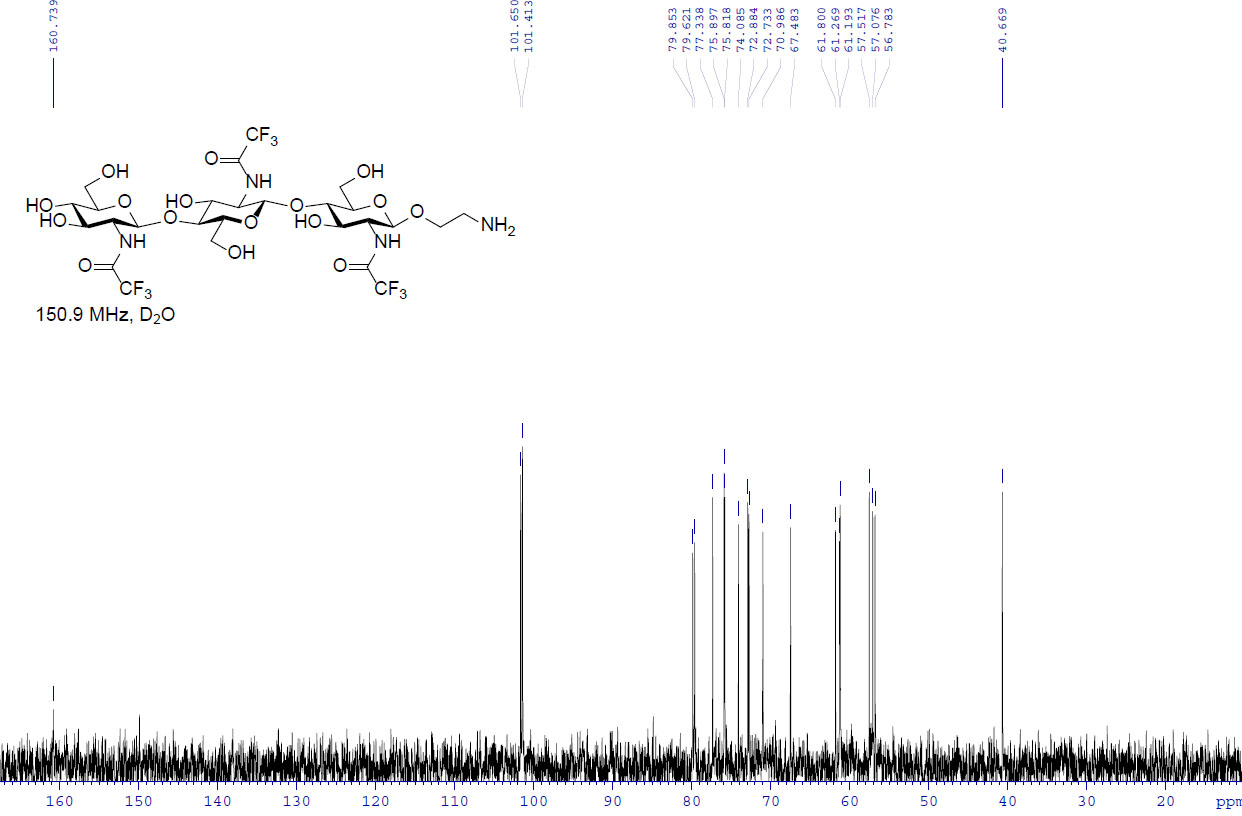


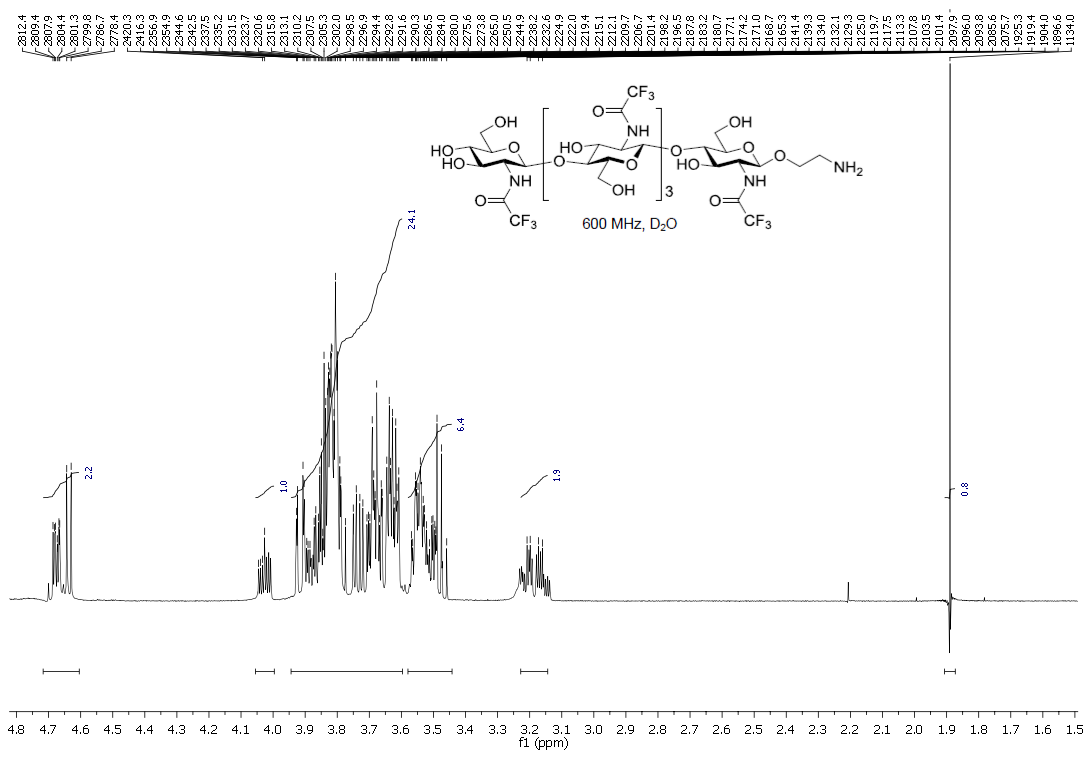


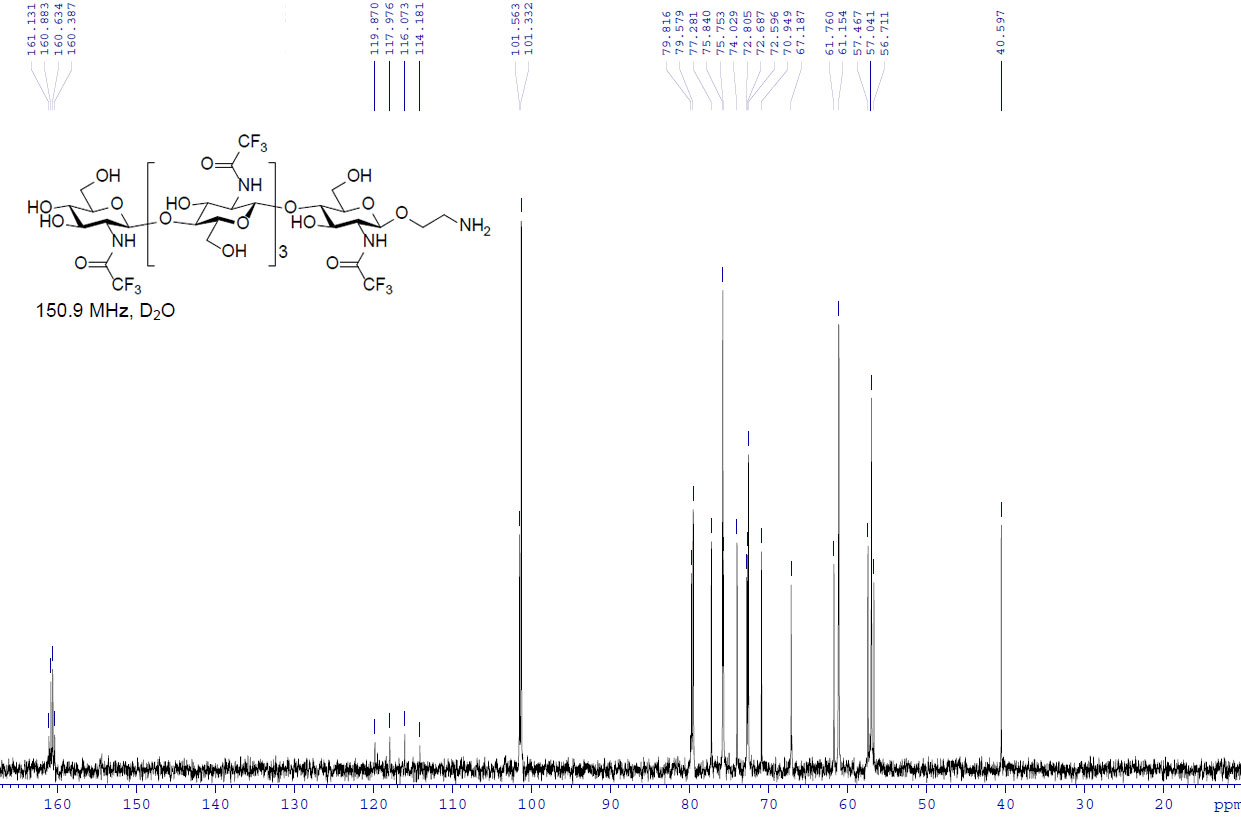


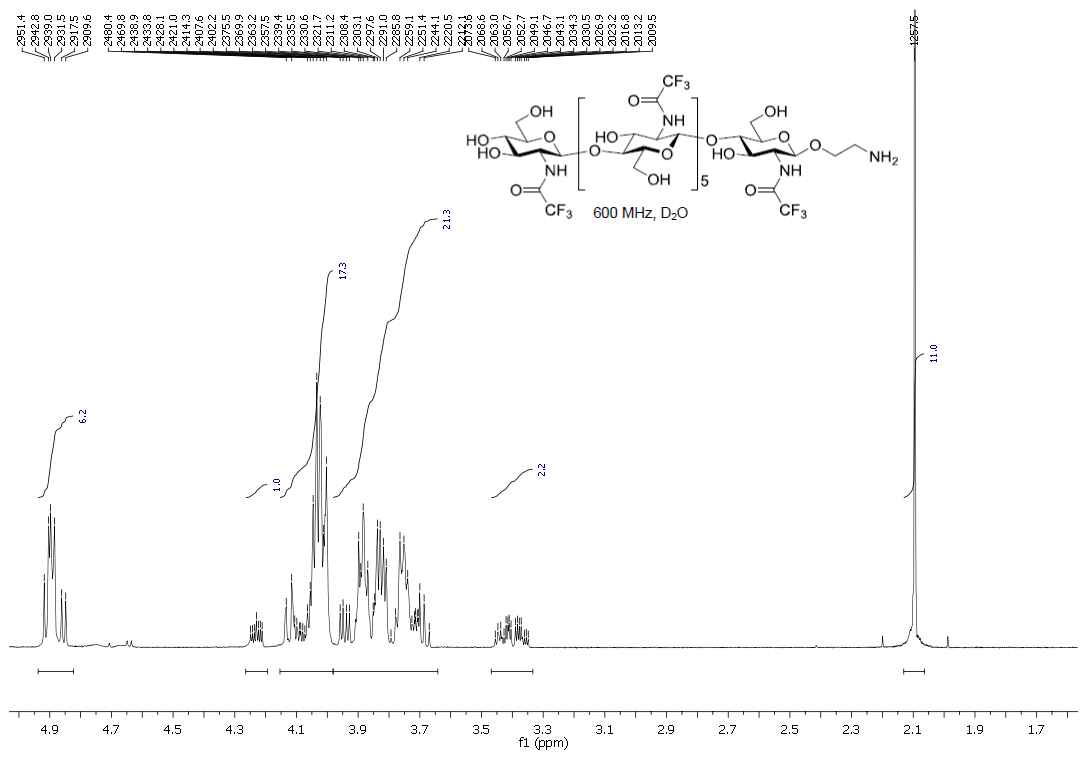


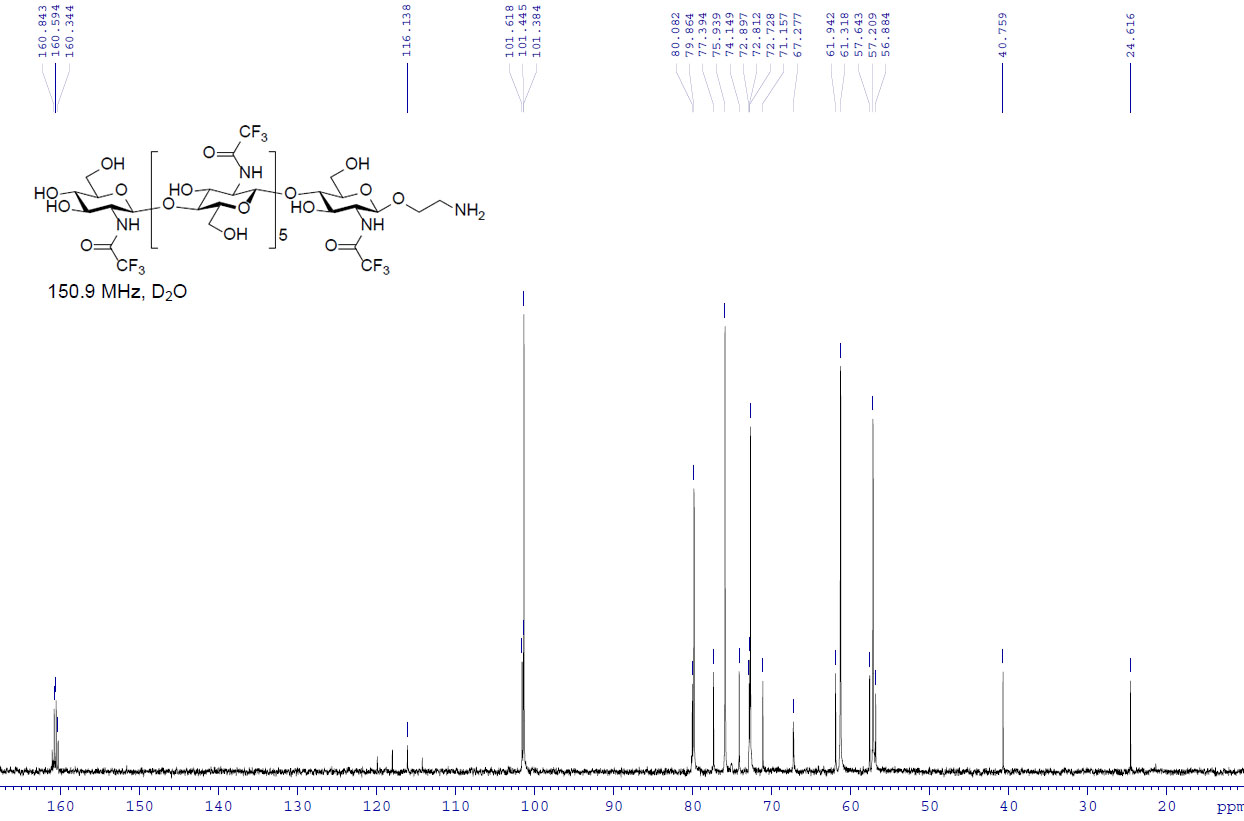


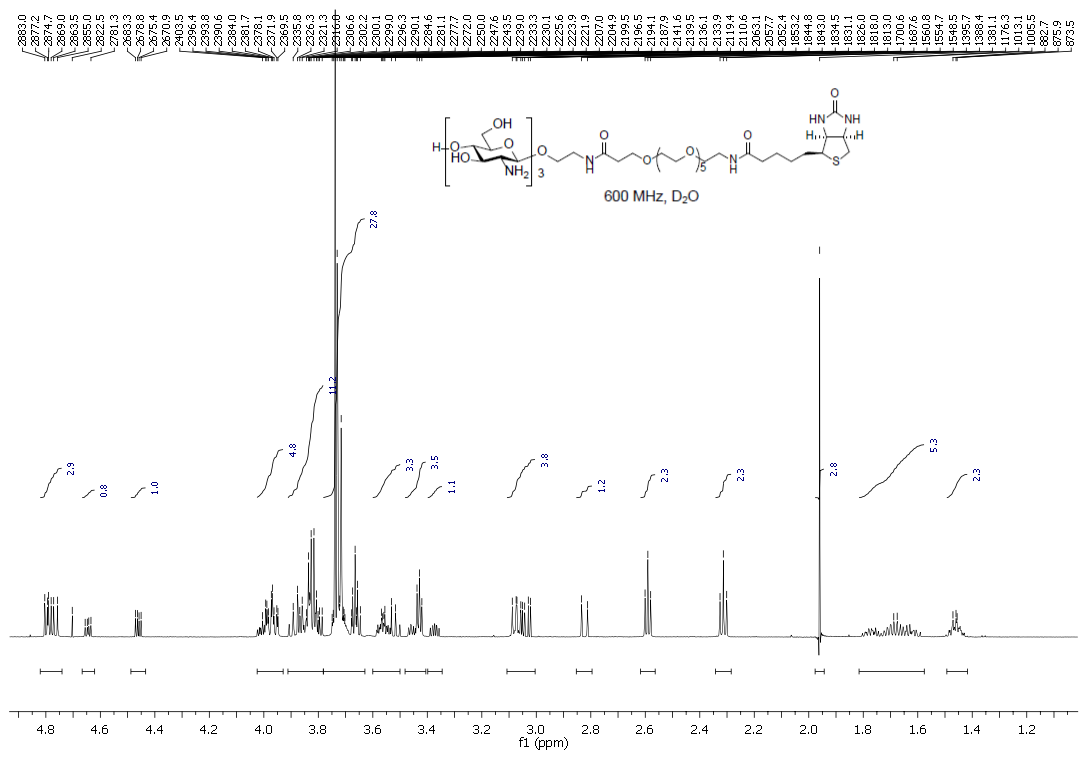


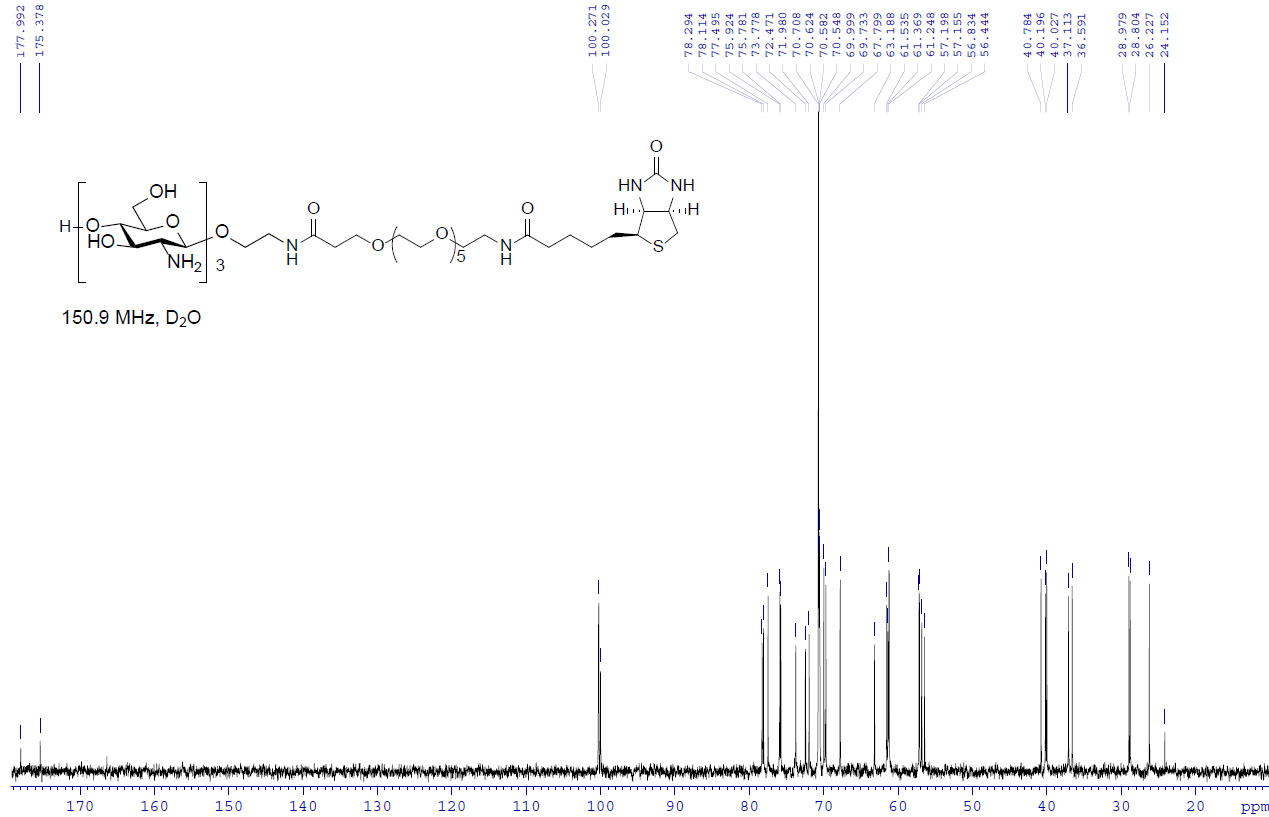


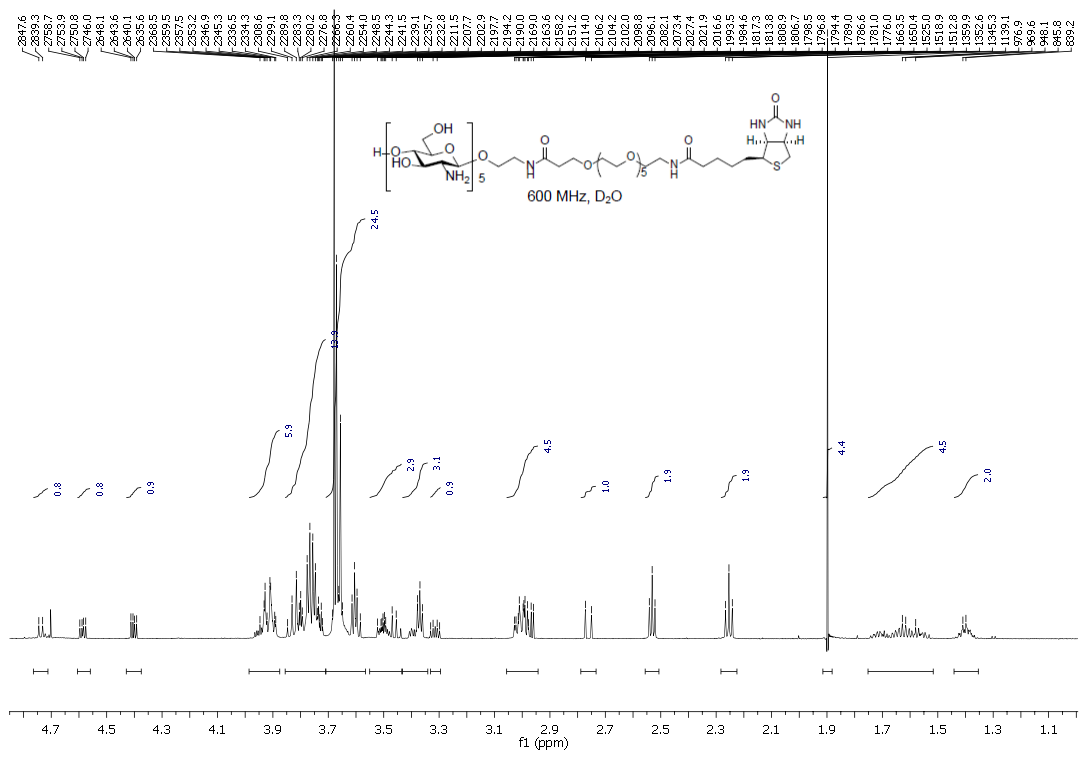


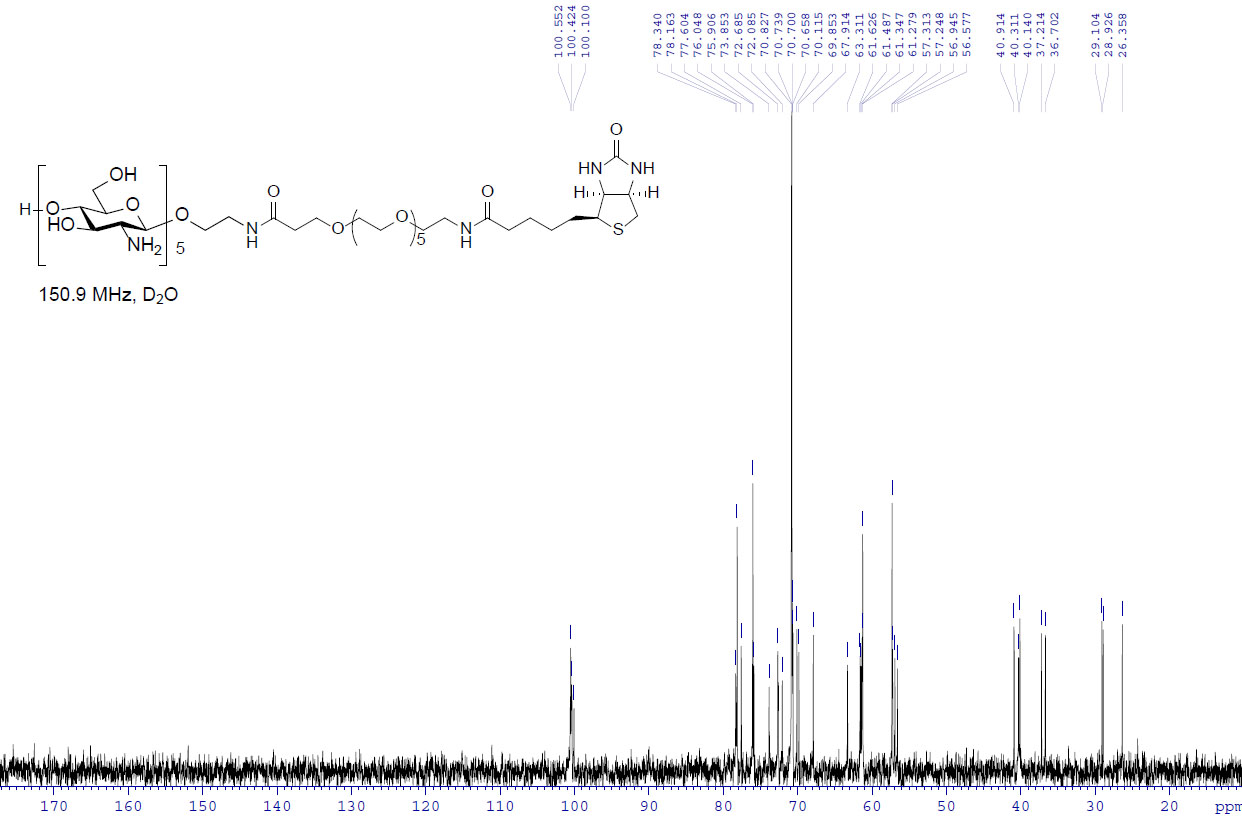


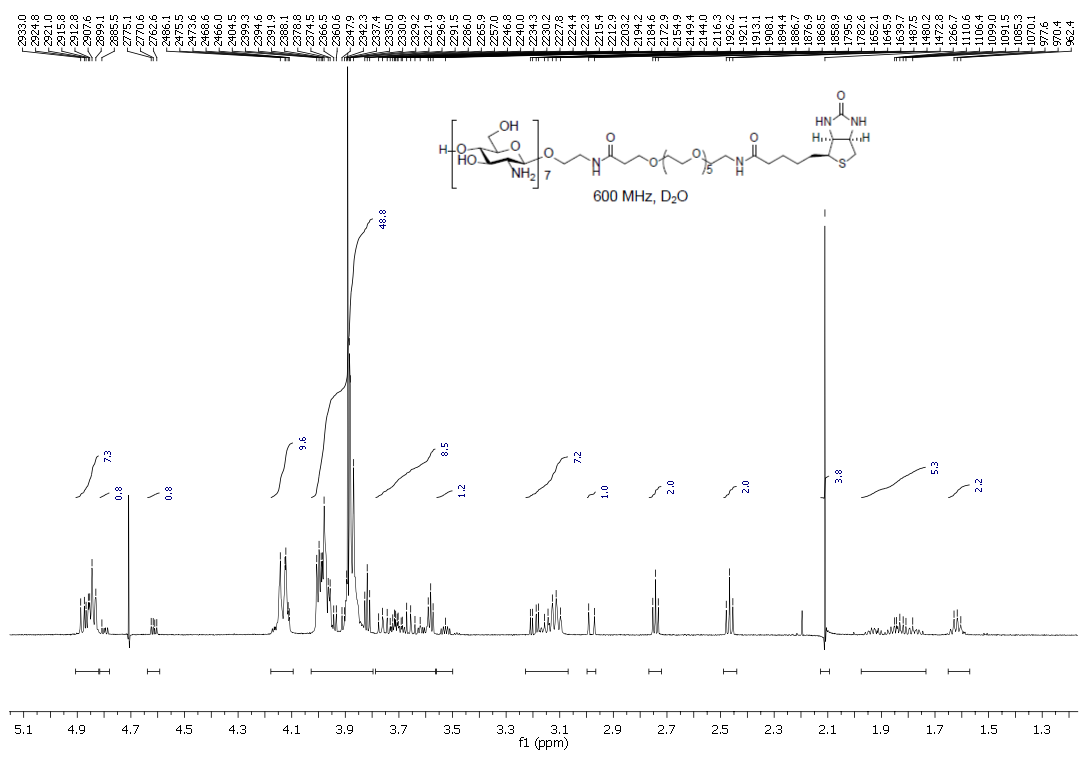


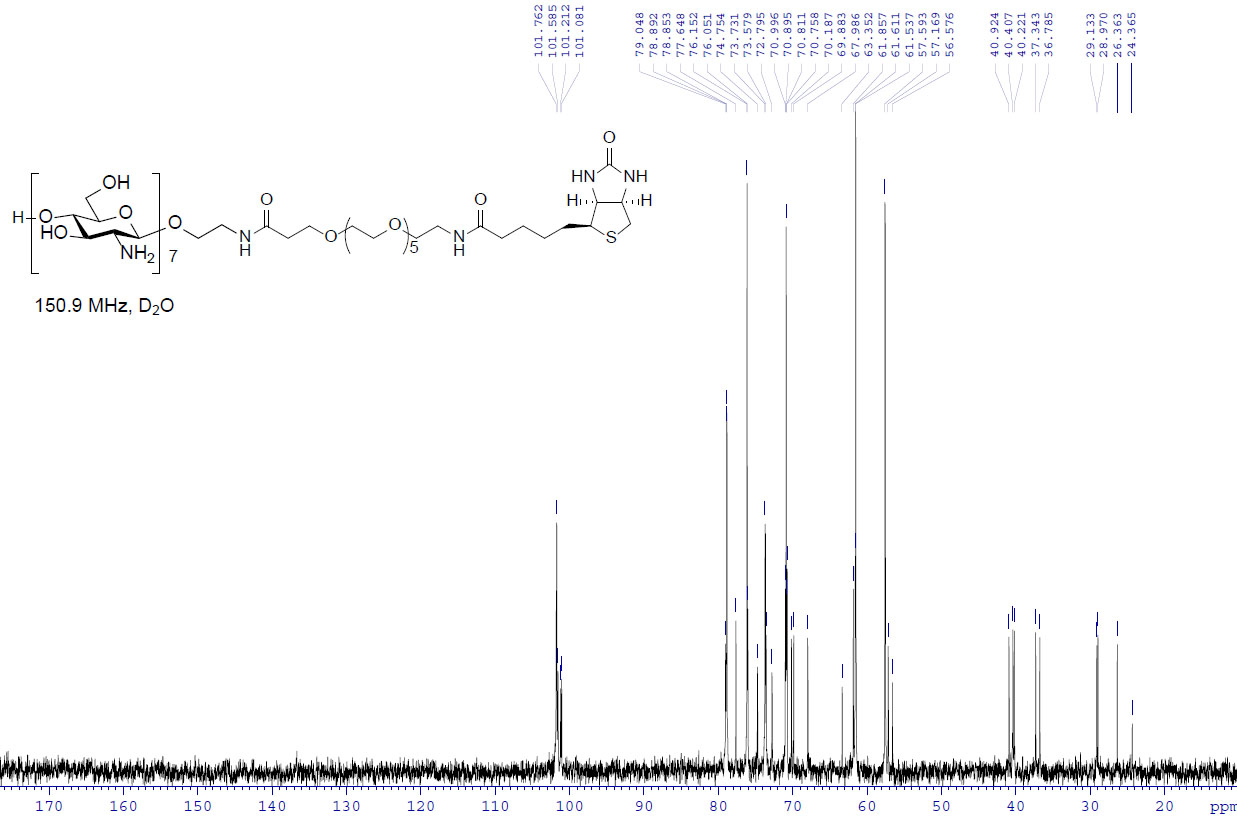


HR mass spectrum of compound **25**

**
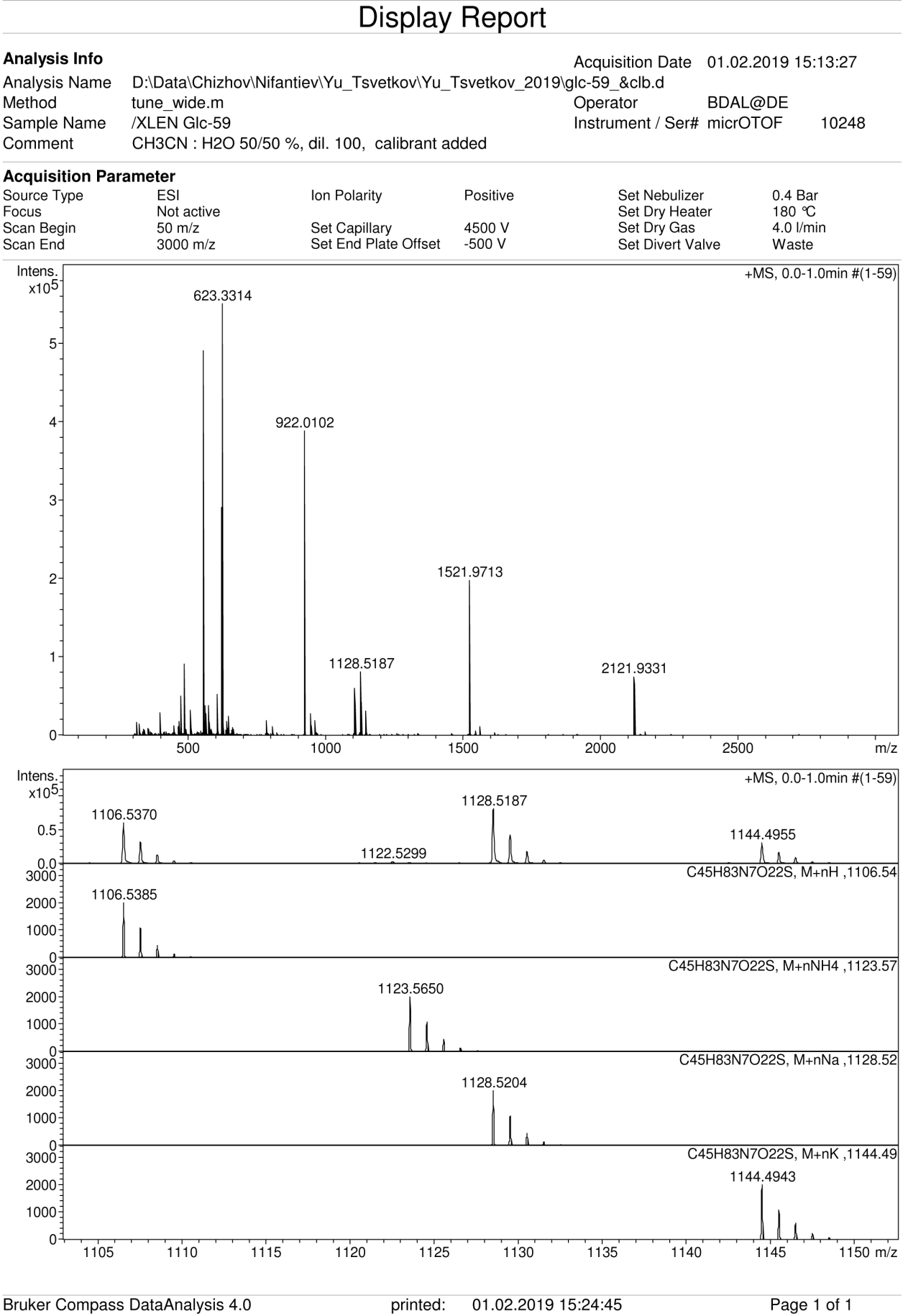
**

**
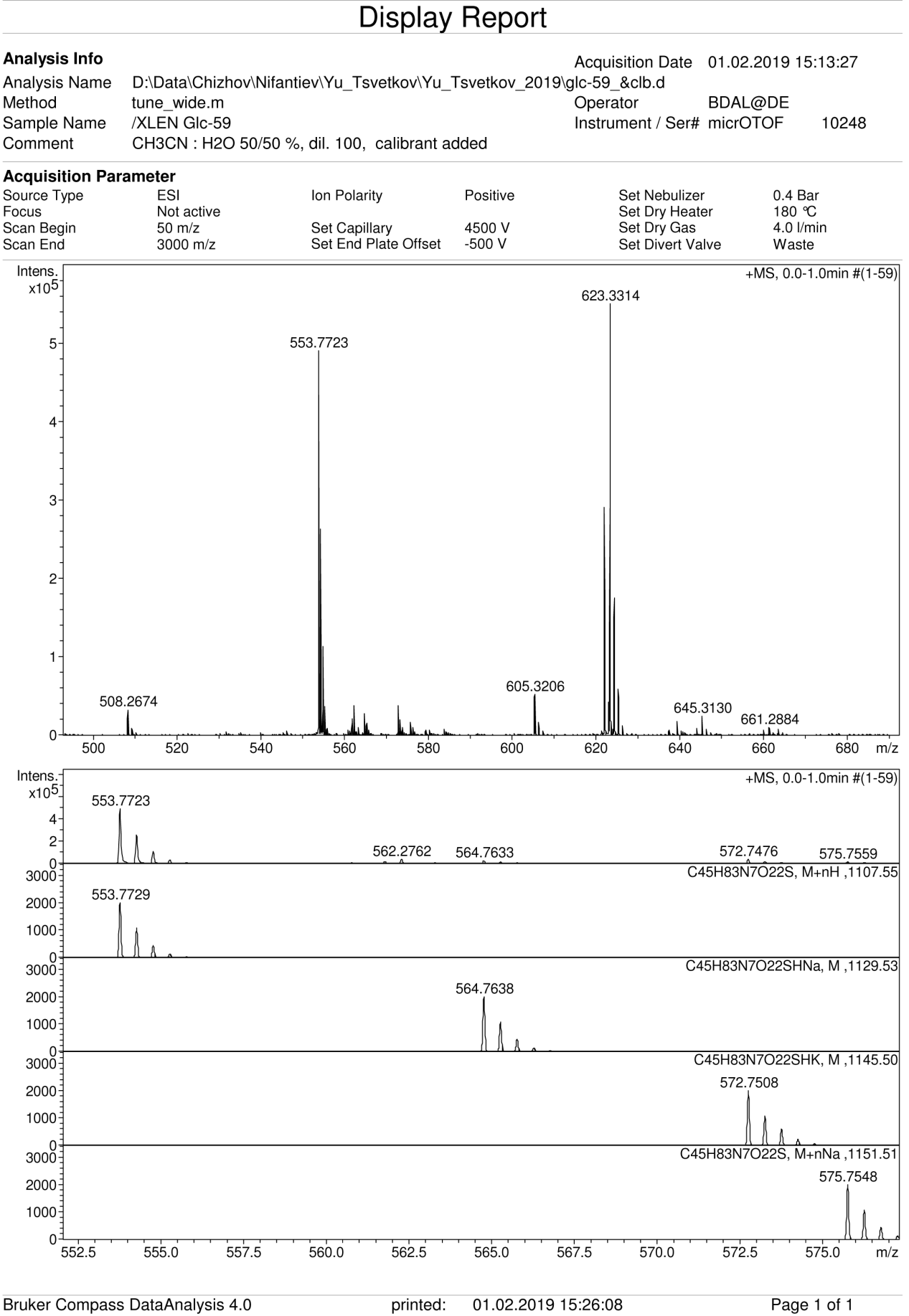
**

HR mass spectrum of compound **26**

**
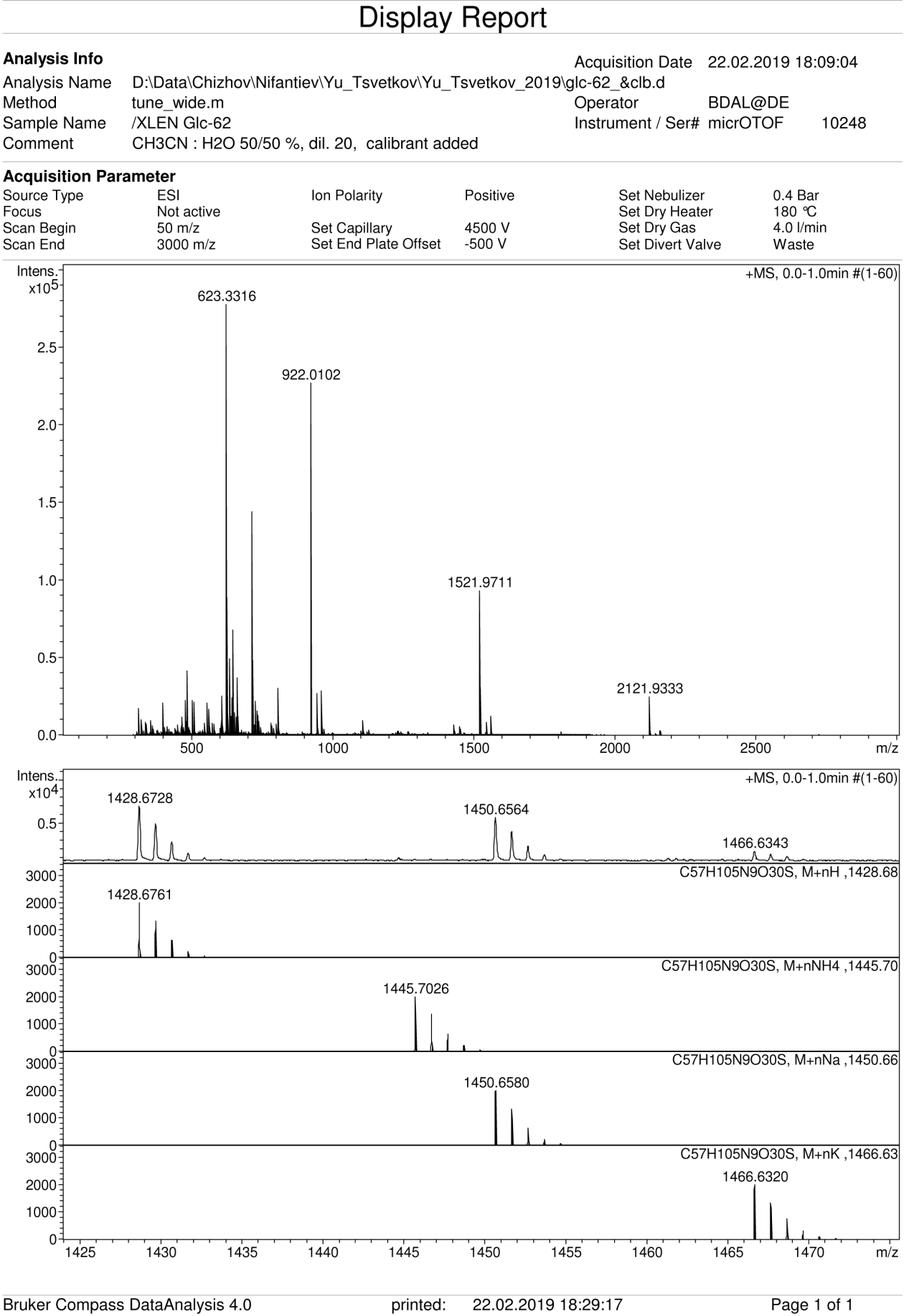
**

**
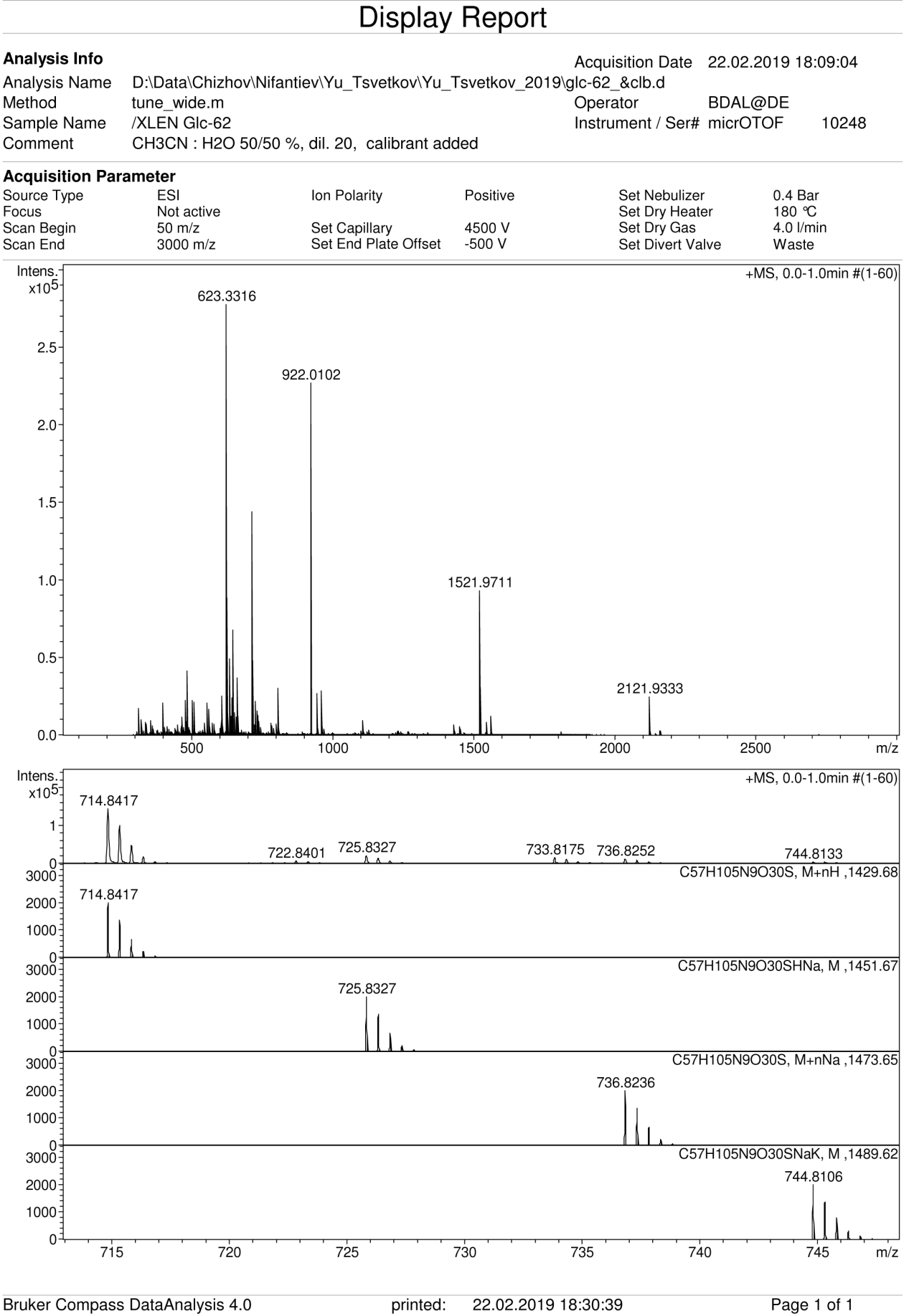
**

HR mass spectrum of compound **27**

**
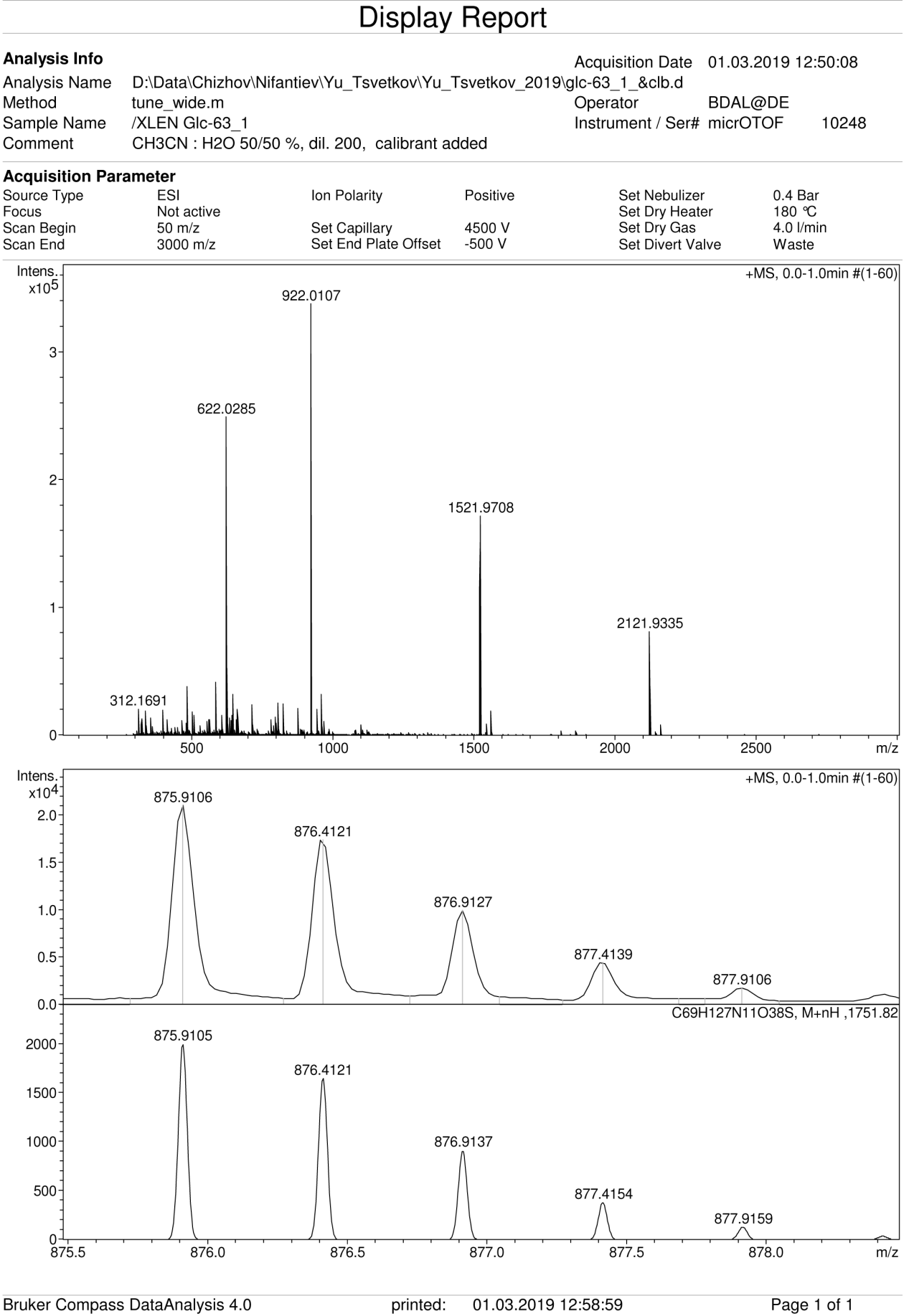
**

**Chitin and chitosan characterisation**

The amount of chitin isolated from *C*. *albicans* cells was approximately 6 % of the mass of the prepared glucan, compared with ≈ 3 % isolated from yeast and ≈ 11 % isolated from mycelium by Ruiz-Herrera et al. (2006). The FTIR spectra of chitin and chitosan confirmed their main structure, namely C==O stretching vibration (amide I) at 1652 cm^–1^; N—H bending and C—N stretching at 1557 cm^–1^ (amide II) and 1307 cm^–1^ (amide III), and N—H deformation (amide IV) at 1259 cm^–1^ were present in the chitin spectrum and absent in the chitosan spectrum (Figure S1) (Sun et al., 2018). According to divided amide I bands (1652 cm^–1^ and 1621 cm^–1^), the isolated chitin exhibited a highly ordered, allomorphic, crystalline form designated as α (Kaya et al., 2017). The molecular mass of the prepared chitosan was estimated from GPC-HPLC to be 80 kDa (peak), with a range of 65–150 kDa. We propose that the molecular mass of isolated chitin was the same or higher, but we did not perform any direct measurement. There are numerous estimation methods for degree of acetylation (DA) or deacetylation (Kasaai, 2009; Czechowska-Biskup et al., 2012). Quantification of individual peak absorbance at 1320 cm^–1^ vs. 1420 cm^–1^ (El Knidri et al., 2017) or 1655 cm^–1^ vs. 3450 cm^–1^ (Domszy and Roberts, 1985; Sabnis and Block, 1997) did not provide consistent results, but the second method indicated that chitin acetylation should be close to 100 %. According to Wagener et al. (2014), the DA of chitin from *C*. *albicans* should be 0.97. Measurement of *N*-acetylglucosamine by UV performed after acid hydrolysis of chitosan (Czechowska-Biskup et al., 2012), indicated (3.9 ± 0.5) % DA, while ^1^H NMR analysis of chitosan (Kasaai, 2009) gave 10.0 % DA (Figure S2).

**
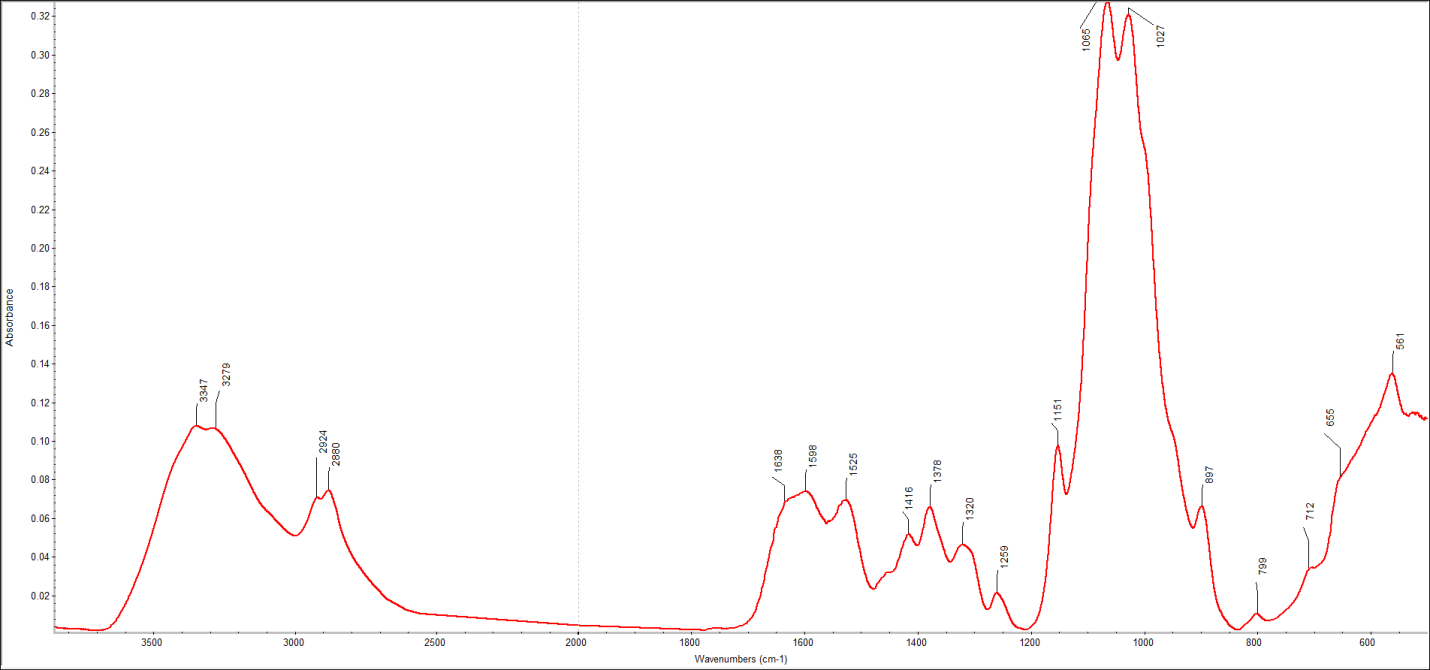
**

**A**

**
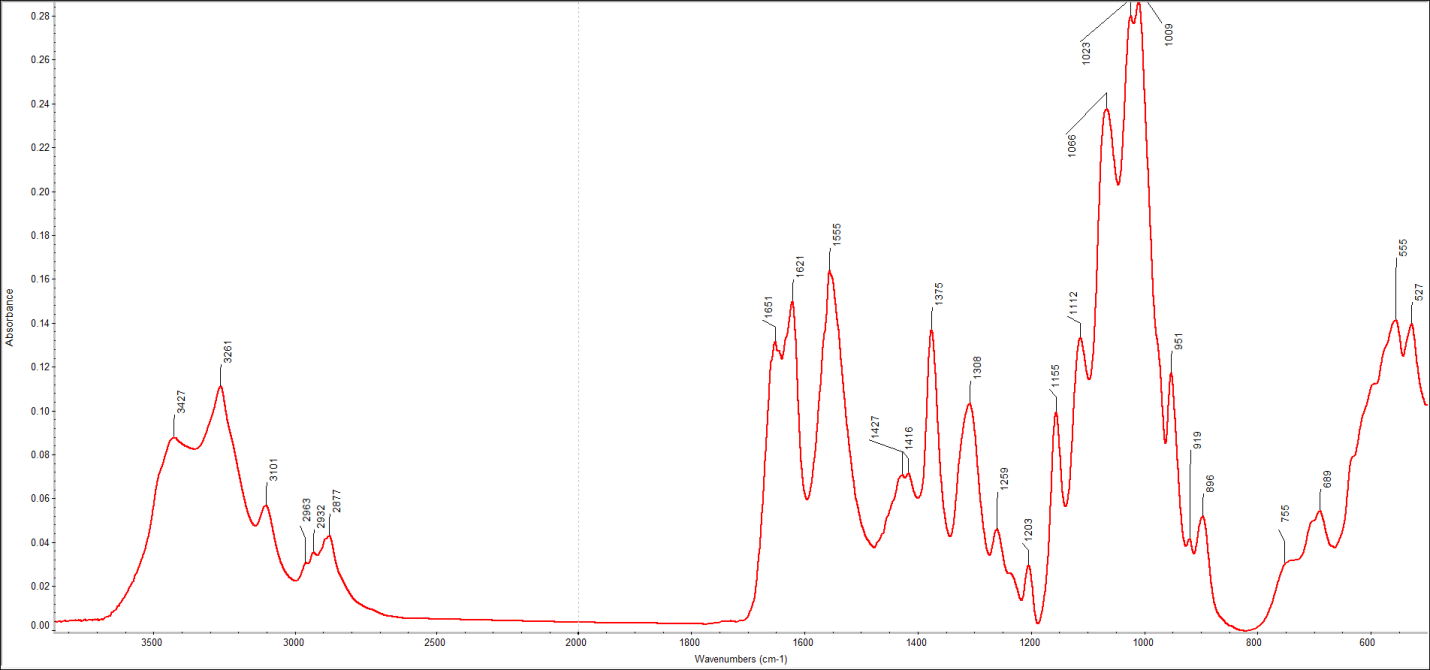
**

**B**

**Figure S1.** FTIR spectra of the prepared chitin (A) and chitosan (B).


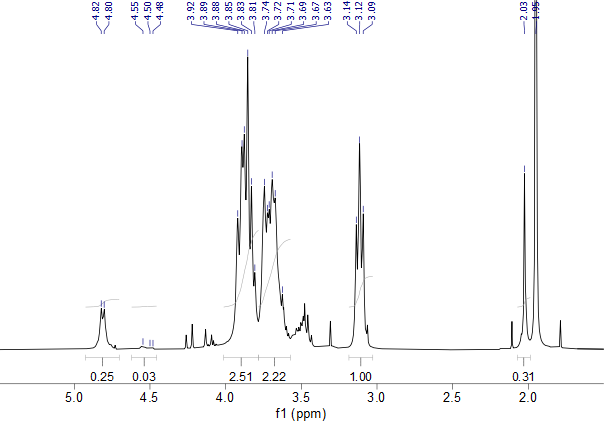


**Figure S2.** ^1^H NMR spectrum of the used chitosan sample.

**References**

Czechowska-Biskup, R., Jarosińska, D., Rokita, B., Ulański, P., and Rosiak, J. M. (2012). Determination of degree of deacetylation of chitosan - Comparision of methods. *Prog. Chem. Appl. Chitin Its Deriv.* 5–20.

Domszy, J. G., and Roberts, G. A. F. (1985). *Makromolekul. Chem*. 186(8), 1671–1677. doi: 10.1002/macp.1985.021860815

El Knidri, H., Belaabed, R., El Khalfaouy, R., Laajeb, A., Addaou, A., and Lahsini, A. (2017). Physicochemical characterization of chitin and chitosan producted from *Parapenaeus longirostris* shrimp shell wastes. *J. Mater. Environ. Sci.* 8(10), 3648–3653.

Kaya, M., Mujtaba, M., Ehrlich, H., Salaberria, A. M., Baran, T., Amemiya, C. T., et al. (2017). On chemistry of γ-chitin. *Carbohydr. Polym.* 176, 177–186. doi: 10.1016/j.carbpol.2017.08.076.

Kasaai, M. R. (2009). Various Methods for Determination of the Degree of N-Acetylation of Chitin and Chitosan: A Review. *J. Agric. Food Chem.* 57(5), 1667–1676. doi: 10.1021/jf803001m

Ruiz-Herrera, J., Victoria Elorza, M., Valentín, E., and Sentandreu, R. (2006). Molecular organization of the cell wall of *Candida albicans* and its relation to pathogenicity. *FEMS Yeast Res.* 6(1), 14–29. doi: 10.1111/j.1567-1364.2005.00017.x

Sabnis, S., and Block, L. H. (1997). Improved infrared spectroscopic method for the analysis of degree of N-deacetylation of chitosan. *Polym. Bull.* 39(1), 67–71. doi: 10.1007/s002890050121

Sun, C., Fu, D., Jin, L., Chen, M., Zheng, X., and Yu, T. (2018). Chitin isolated from yeast cell wall induces the resistance of tomato fruit to Botrytis cinerea. *Carbohydr. Polym*. 199, 341–352. doi:10.1016/j.carbpol.2018.07.045

Wagener, J., Malireddi, R. K. S., Lenardon, M. D., Köberle, M., Vautier, S., MacCallum, D. M., et al. (2014). Fungal chitin dampens inflammation through IL-10 induction mediated by NOD2 and TLR9 activation. *PLoS Pathogens* 10(4), e1004050. doi: 10.1371/journal.ppat.1004050.
